# Multi-day recalibration-free electrophysiology interface based on carbon nanotube thin film dry electrodes

**DOI:** 10.1038/s41467-026-78169-6

**Published:** 2026-10-13

**Authors:** Yuhan Wen, Hanna Hedwig Schlegel, David Fox, Canran Wang, Avetik Harutyunyan, Rui Gao, Wei Gao, Qiang Zhang, Preeya Khanna, Ana Claudia Arias

**Affiliations:** 1 Department of Electrical Engineering and Computer Sciences, University of California Berkeley, Berkeley, CA USA; 2 Honda Research Institute USA, Inc., San Jose, CA USA; 3 Andrew and Peggy Cherng Department of Medical Engineering, California Institute of Technology, Pasadena, CA USA; 4 Department of Naval Architecture and Ocean Engineering, Shanghai Jiao Tong University, Shanghai, China; 5 Department of Neuroscience, University of California Berkeley, Berkeley, CA USA

**Keywords:** Electrical and electronic engineering, Carbon nanotubes and fullerenes, Biomedical engineering, Motor control

## Abstract

Electrophysiological signals provide crucial and reliable information for decoding behavioral and cognitive human states. However, existing electrode interfaces are susceptible to artifacts or drift over time, thereby requiring frequent machine-learning model retraining. Here we present an end-to-end design of dry electrodes based on carbon nanotube (CNT) thin films, synthesized by a roll-to-roll floating catalyst chemical vapor deposition process. By balancing structural and electrical properties, we achieved low electrode-skin impedance, high signal fidelity, minimal motion artifacts, high breathability, and biocompatibility, outperforming the gold standard wet electrodes. In a multichannel myoelectric interface demonstration, these CNT electrodes maintained stable signals and consistent task performance across 14 days using only minutes of training data and no further model retraining. To our knowledge, this is the first demonstration of a multi-day recalibration-free dry electrode system under naturalistic conditions, achieved by stable electrode–skin interface, rather than ongoing algorithmic adaptation.

## Abstract (web summary)

Long-term wearable electrophysiology requires high signal fidelity and stable skin–electrode interfaces. Here, the authors develop breathable carbon nanotube dry electrodes that outperform wet electrodes and sustain multichannel EMG control for 14 days without decoder re-training.

## Introduction

While the human skin provides a unique electrical interface for decoding the physiological, cognitive, and behavioral human states^1–4^, it remains to be fully leveraged in wearable systems due to sensor limitations in providing low-noise, stable signals over extended wear^5,6^. Advancements in machine learning frameworks can now fuse multiple biosignals, such as electrocardiography (ECG) for cardiac health^7,8^, electromyography (EMG) for muscle activities^9,10^, electroencephalography (EEG) for mental states^11,12^, and electrooculography (EOG) for eye tracking^13–15^ into high-dimensional representations of user state^16–19^. However, these representations cannot be monitored over long durations without frequent recalibration^20–22^.

A critical barrier to long-term stable recording is the electrode-skin interface^23,24^. Wet electrodes are the gold standard for signal quality, but often degrade with gel dehydration, cause skin irritation, and yield inconsistent signals within hours^25^. Dry electrodes reduce skin irritation and remain stable over time at the cost of higher impedance and noise levels^6^. They are more susceptible to motion artifacts, thermal drifts, and electromagnetic interference^26,27^, take up to 50 minutes to stabilize^28^, and experience greater session-to-session inconsistency^29^. As a result of these challenges (Table S1), machine learning (ML) models trained under initial conditions often degrade over time and require periodic recalibration or retraining. This is impractical for edge and embedded wearable systems, where computational resources, power, and memory are limited, and frequent calibration compromises user experience^30,31^.

Several strategies have been studied to improve electrode performance. Tattoo-style dry electrodes, such as ultrathin metal films^32–35^ and nanomaterial networks^36–38^, leverage conformability to improve impedance and contact quality, but face trade-offs among conductivity, mechanical stability, and manufacturing scalability^39–41^. Poly(3,4-ethylenedioxythiophene) (PEDOT) has emerged as a leading material due to its mixed ionic–electronic conductivity^42,43^, but often degrades under oxidative conditions, such as sweat and elevated temperatures, and is prone to mechanical failure from repeated strain^44–46^. Other approaches use micromachining and nanofabrication to create 3D structures that increase the effective contact area and improve skin coupling, but add fabrication complexity^39–43^. Moreover, long-term stability is often evaluated by the continued ability to record signals, maintain low contact impedance or a high signal-to-noise ratio (SNR), or preserve waveform features. While important, these metrics do not necessarily ensure stable long-term downstream performance in high-channel-count ML systems^44^, which remains a gap in existing dry electrode studies.

**Fig. 1:**
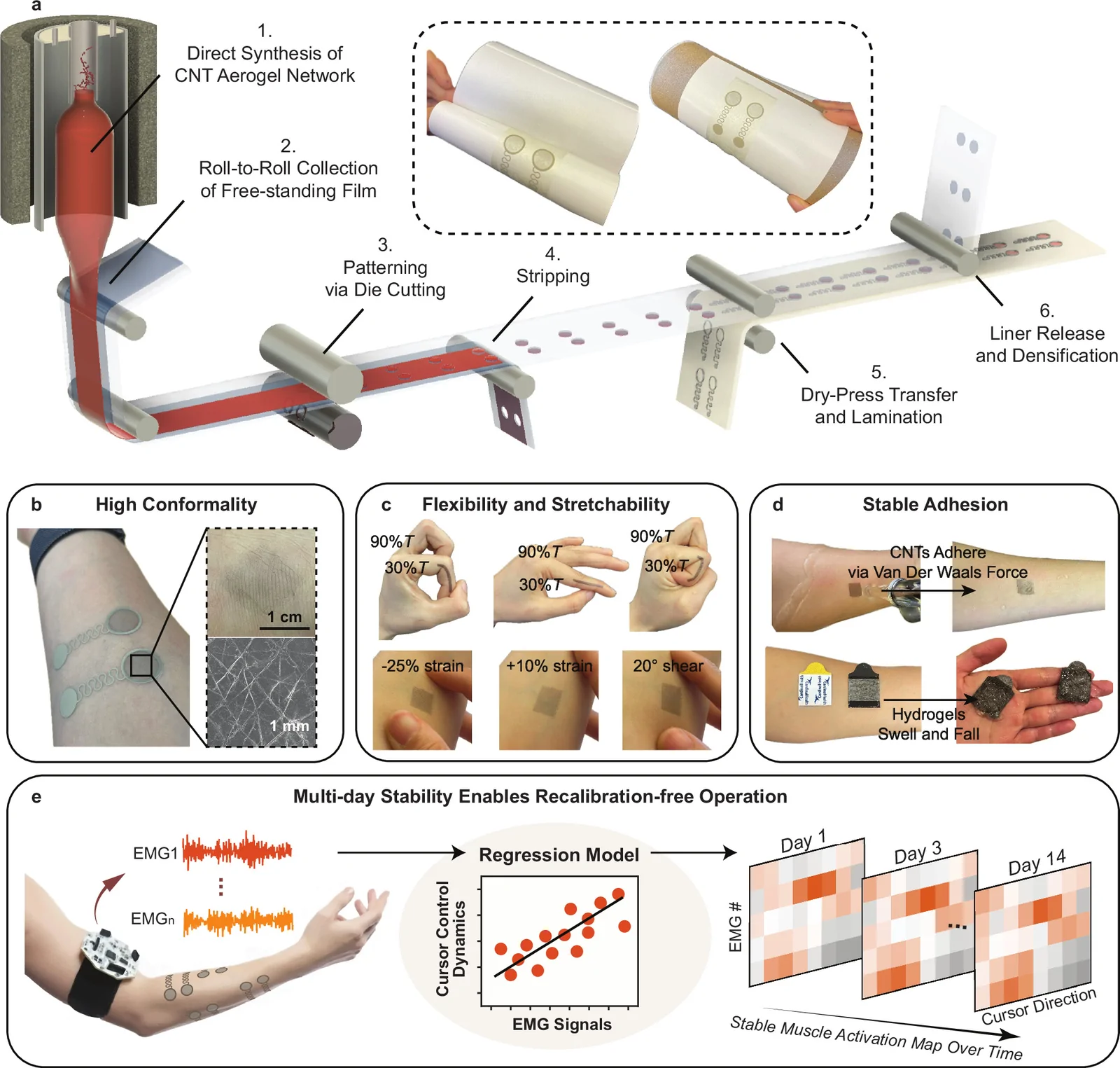
Roll-to-roll manufacturing and performance of a conformal dry electrode for stable myoelectric interfacing. **a** A continuous fabrication process for producing patterned carbon nanotube (CNT) aerogel films as ultrathin dry electrodes. **b** The resulting electrodes exhibit high conformability on the skin. Insets: photograph of the CNT film on a human subject’s palm (scale bar, 1 cm) and an SEM image of the CNT network’s microstructure on a skin replica (scale bar, 1 mm). **c** Mechanical compliance of the CNT film of varying optical transmittances (%*T*) is demonstrated under bending, stretching (−25% and +10% strain), and twisting (20° shear). **d** CNT films achieve stable adhesion via van der Waals forces, providing a stable interface compared to hydrogel-based electrodes, which swell and fall off after showering. **e** The stability of the electrode-skin interface enables consistent multi-day electromyography (EMG) recording. A static decoder, trained once on Day 1 can consistently map EMG signals to decoded computer cursor gestures in subsequent sessions, resulting in very consistent muscle activation patterns for the same gestures across 14 days.

In this work, we present a conformal carbon nanotube (CNT)-based dry electrode system that maintains a low-impedance, highly stable electrode–skin interface and preserves ML decoder performance for over two weeks without recalibration (Fig. 1). This is achieved using a modified floating catalyst chemical vapor deposition (FCCVD) process^45^, in which CNTs form a stable, entangled aerogel network directly during synthesis and can be collected and assembled roll-to-roll (Fig. 1a). We further developed a theoretical framework to study the roles of contact density, conformability, and sheet resistance on contact impedance. The optimized CNT dry electrodes are conformal, stretchable, flexible, breathable, and adhesive via van der Waals interactions^46^ (Fig. 1b–d). We demonstrated ECG, EMG, and EEG recordings, with contact impedance (13.7 kΩ·cm^2^ at 10 Hz) and SNR outperforming gold-standard wet electrodes. In addition, a continuously worn myoelectric interface was implemented and showed negligible signal degradation and required no ML model recalibration to maintain task performance over 14 days (Fig. 1e). To our knowledge, this is the first demonstration of a dry electrode system that maintains recalibration-free, multi-day ML inference stability under naturalistic conditions by preserving a stable electrode–skin interface, rather than relying on ongoing algorithmic adaptation. This result addresses a core limitation in uninterrupted, long-term, ambulatory ML-driven wearable systems beyond clinical environments.

## Results

### Material synthesis and characterization

**Fig. 2:**
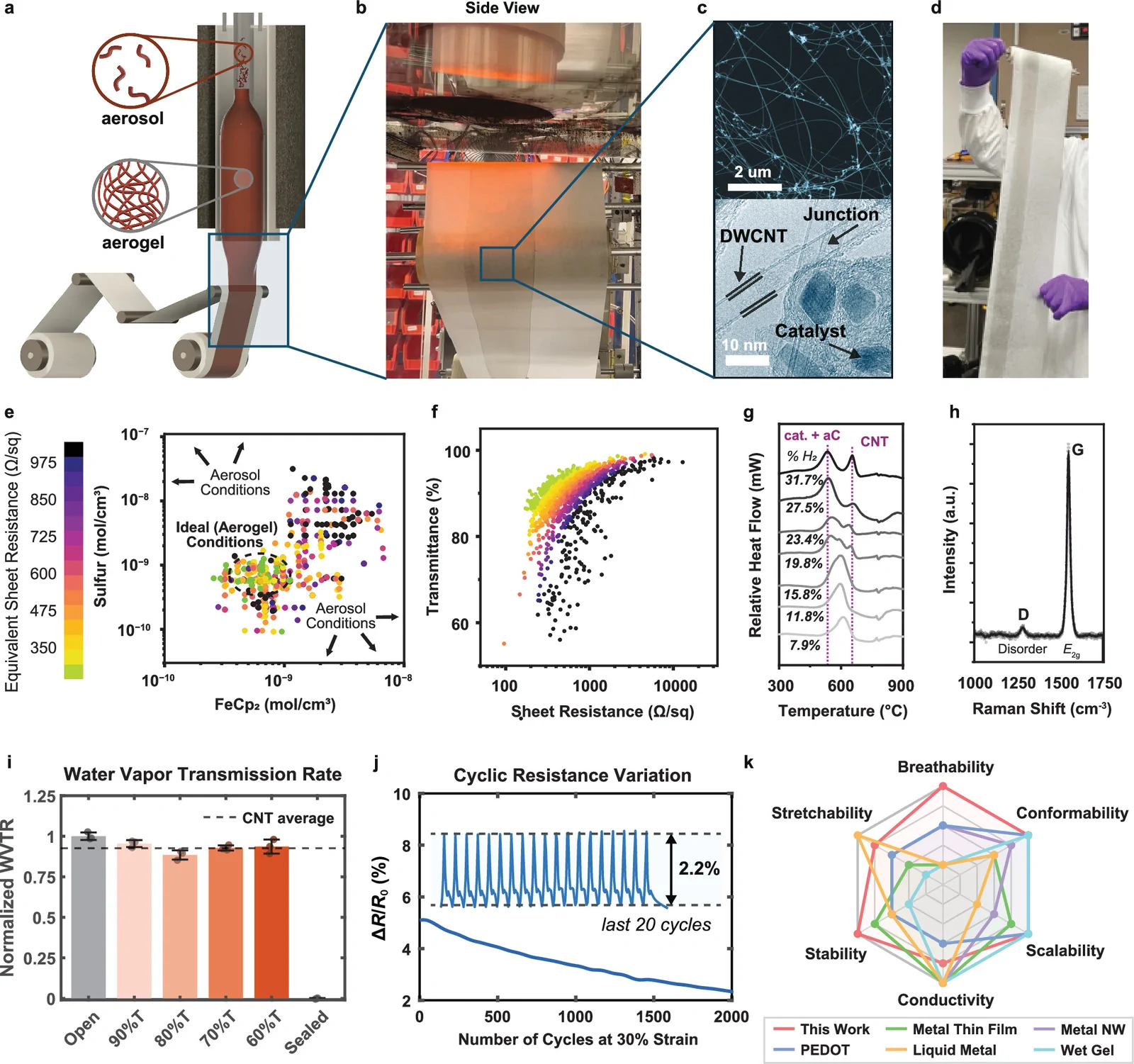
Roll-to-roll synthesis and characterization of carbon nanotube thin films. **a** Schematic of the floating catalyst chemical vapor deposition (FCCVD) process for continuous, roll-to-roll synthesis of CNT films. **b** Photograph of the large-scale production of the CNT aerogel as it is drawn from the furnace. **c** Scanning electron micrograph (top) and transmission electron micrograph (bottom) showing the porous, interconnected network of double-walled carbon nanotubes (DWCNTs). **d** Photograph demonstrating meter-scale production of CNT film. **e** Parameter space screening of ferrocene (FeCp_2_) and sulfur concentrations and the ideal aerogel conditions. **f** Relationship between optical transmittance and sheet resistance of the synthesized CNT films. **g** Differential scanning calorimetry (DSC) thermograms, showing improved CNT quality and purity with increasing hydrogen (H₂) concentration during synthesis. **h** Representative Raman spectrum of the CNT film, with the high G-to-D peak ratio indicating high crystallinity. **i** Normalized water vapor transmission rate (WVTR) for CNT films of varying optical transmittance (%*T*) compared to open-air and sealed controls, demonstrating high breathability. Data represent mean ± SD (*n* = 3 independent films per transmittance group). **j** Relative resistance change (Δ*R*/*R*_0_) of 90%*T* CNT film over 2000 cycles at 30% strain. **k** Radar plot benchmarking material properties of the CNT film in this work against PEDOT, metal thin film, metal nanowire, liquid metal, and wet gel across six performance metrics relevant to electrophysiology monitoring: breathability, conformability to skin, manufacturing scalability, material conductivity, electrochemical stability, and stretchability.

Combining the process of extrusion-blown plastic film and floating catalyst chemical vapor deposition (FCCVD) of few-walled carbon nanotubes (CNT), roll-to-roll large-scale production of free-standing CNT thin film is demonstrated (Movie S1). The method is known as blown aerosol chemical vapor deposition (BACVD)^45^ (Fig. 2a–d). Instead of growing CNTs on a substrate or collecting a loose aerogel, the BACVD process injects a carbon feedstock and carrier gas into a high-temperature reaction zone (1000–1200 °C), where CNTs nucleate and grow in the gas phase to form a CNT aerosol. An auxiliary gas stream then aerodynamically inflates the aerosol into a stable, freestanding aerogel tube, composed of a percolated CNT network. This aerogel structure is continuously drawn from the furnace, flattened, and collected by a roll-to-roll system. This strategy achieves a yield exceeding 10 m^2^ of CNT film per hour, eliminates the need for dispersion and surfactant, and resolves the trade-off between loading percentage and stretchability in composite materials.

To obtain the best-performing CNT on-skin electrode, the reduction of CNT film resistivity, characterized by the equivalent sheet resistance at 90% Transmittance (*R*_eq_), was essential. This metric is a function of the properties of the individual CNTs, such as their length^47^, wall-number, diameter^48^, and crystallinity, as well as the CNT network properties, such as junction type and presence of electronic barriers. To alter these properties, reactor parameters, such as the furnace temperature, reactor configuration, and concentration of reagents in the gas stream, can be adjusted. The critical parameters in this study were the concentrations of ferrocene (FeCp_2_) and sulfur in the feedstock gas. The aerogel synthesis windows are highlighted in Fig. 2e and appear centered at 6 × 10^−10^ mol·cm^−3^ for ferrocene and 8 × 10^−10^ mol·cm^−3^ for sulfur. Screening across this parameter space confirmed that film properties were dependent on both concentrations, with optimized conditions yielding undoped *R*_e__q_ below 250 Ω/sq (Fig. 2f).

Prior to screening for FeCp_2_ and sulfur, we ensured other aspects of the reagent stream, such as total flow and H_2_ concentration, were calibrated to ensure a stable and continuous fabrication of CNT blown aerogel films. Films prepared in a concentration of H_2_ less than 19%, could not demonstrate a *R*_eq_ less than 700 Ω/sq. Samples prepared at different H_2_ concentrations were characterized with differential scanning calorimetry (DSC) as shown in Fig. 2g. The splitting of one peak centered at 612 °C into two peaks, centered at 535 °C and 655 °C, was observed. The first of the two peaks is attributed to amorphous carbon and catalyst particles, which oxidize upon carbon shells being removed. The second peak is attributed to higher-quality CNT material, which resists oxidation until 643 °C. This applies to all CNT films prepared in H_2_ concentrations of 20% or higher. After observing this trend, the concentration of H_2_ was kept above 20% for all samples while performing the parameter space sweep of FeCp_2_ and sulfur concentration. Optimized conditions yielded G/D ratios exceeding 30 (Fig. 2h), indicating highly graphitic networks with minimal amorphous carbon or tube breakage. These values align with the low junction resistance measured. The optimized synthesis window used a reactor temperature of 1200 °C, FeCp_2_ concentrations of 4 × 10^−10^ to 1 × 10^−9^ mol·cm^−3^, sulfur concentrations of 1 × 10^−10^ to 1 × 10^−9^ mol·cm^−3^, 19–25% H_2_, and a total gas flow rate of 1800–2200 sccm. These conditions were used for all subsequent electrode fabrication. Stability of the synthesis conditions, including transmittance and sheet resistance drift (Fig. S2), further demonstrated scalability of the synthesis method.

The CNT network morphology is shown in the electron micrographs in Fig. 2c (Top). Some catalyst particles and amorphous carbon debris can be observed across the film, often trapped near junctions of multiple nanotubes. Nanotube diameter followed a Gaussian distribution (Fig. S11), with a mean of 4.36 nm and a standard deviation of 0.212. The diameter size is consistent with reports that junction resistance should be smaller in larger diameter tubes, which typically have low band gaps. The transmission electron micrographs in Fig. 2c (Bottom) also highlight the larger diameter and few-walled nature of the CNTs used in this study.

The CNT network’s open, porous structure allowed good breathability and a high water vapor transmission rate (WVTR) of 4.9 mg cm^-^^2^ h^-^^1^ across various thicknesses (Fig. 2i). This value is 95% of the open-air control (5 mg cm^−2^ h^−1^) and surpasses the natural perspiration rate of human skin (0.83–2.08 mg cm⁻^2^ h⁻^1^)^49^, thereby mitigating the risk of moisture accumulation and irritation common with non-breathable electrodes. Furthermore, cyclic mechanical testing (2000 cycles at 30% strain) in Fig. 2j demonstrated a resistance change of 5%. This ensures outstanding mechanical and electrical stability under repeated skin deformation, typically 10-30% during typical daily motions^50^. To evaluate sweat-relevant electrochemical stability, CNT films were immersed in artificial sweat at 37 °C and showed stable impedance responses over four weeks (Fig. S9).

Compared with PEDOT films, metal nanowires, metal thin film, liquid metal, and conventional wet gel electrodes, these CNT films provide a balanced combination of breathability, conformability, conductivity, stability, stretchability, and manufacturing scalability^29,51–56^ (Fig. 2k). Note that these ratings only represent material-level properties, and device-level performance can be improved by structural designs, special formulation, and architecture choice^23^. A comprehensive comparison with literature benchmarks is available in Table S2-S4.

### Electrode impedance and optimization

**Fig. 3:**
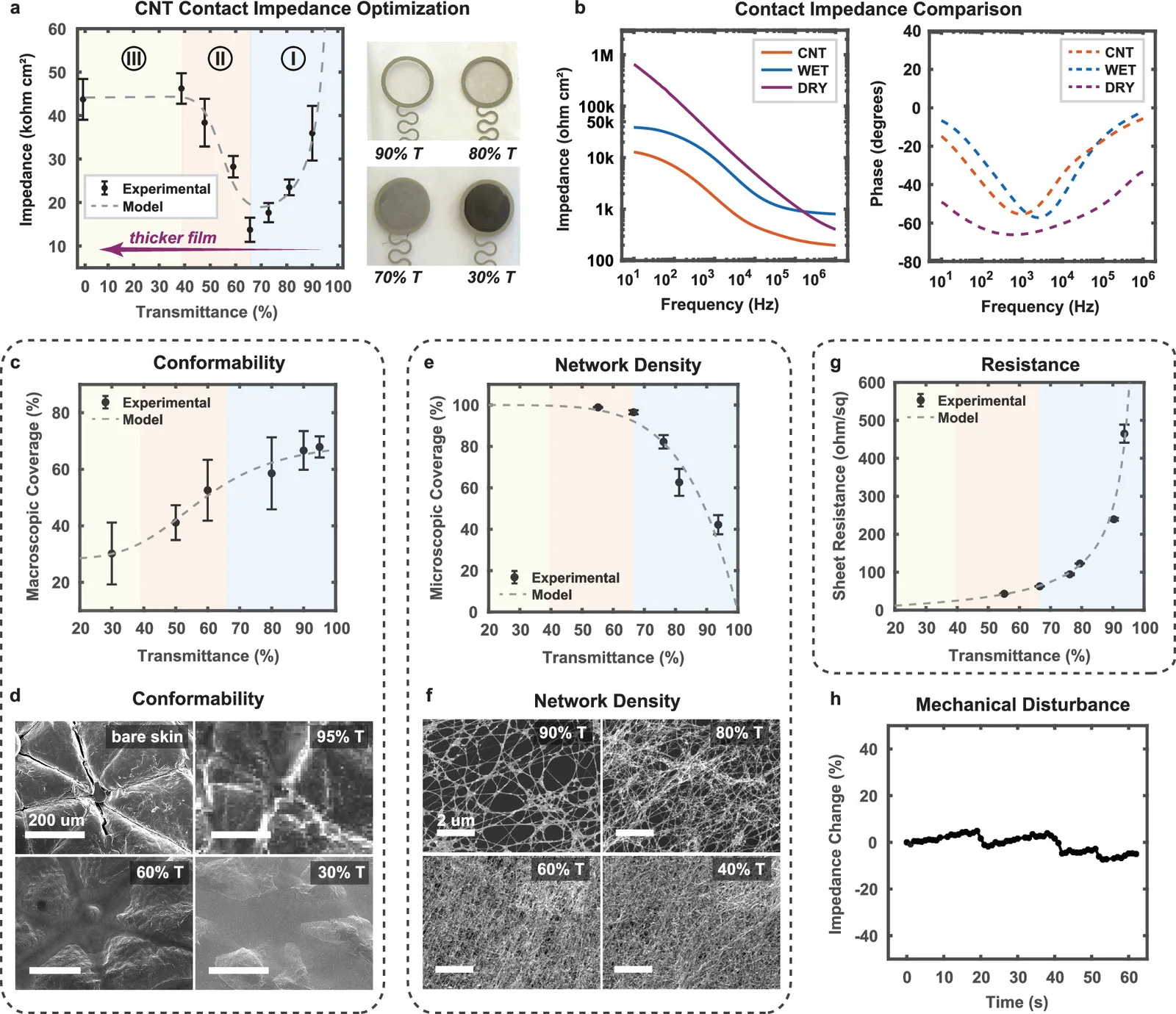
Optimization and characterization of the CNT electrode-skin contact impedance. **a** Contact impedance at 10 Hz as a function of the CNT film’s optical transmittance. An optimal contact impedance is found at ~65% transmittance (13.7 ± 2.8 kΩ·cm^2^, mean ± SD; *n* = 8 independent devices evaluated on the same human subject), defining three operational regimes: (I) dominated by poor conformability in thick films, (II) an optimal balance, and (III) limited by low network density in thin films. Insets show photographs of films with varying transmittance. **b** Contact impedance of the optimized CNT electrode compared with standard wet and conventional dry electrodes. **c** Macroscopic skin coverage versus film transmittance (mean ± SD; *n* = 3 independent samples). **d** SEM images of the film-skin interface for various film thicknesses. Scale bars, 200 µm. **e** Microscopic network coverage increases with film thickness and decreasing transmittance (mean ± SD; *n* = 3 independent samples). **f** SEM images visualizing the increase in CNT network density as transmittance decreases. Scale bars, 2 µm. **g** Sheet resistance as a function of film transmittance (mean ± SD; *n* = 3 independent samples). **h** Mechanical stability test showing the real-time percentage change in contact impedance under mechanical disturbance (*n* = 4).

Contact quality is the key challenge to designing dry electrodes. With a thickness of only a few nanometers, the CNT film conforms well to small-sized features while maintaining good stability. The high porosity gives the dry electrode a low fill factor and intrinsically high breathability. In addition, the large specific surface area and nanostructure of the CNTs maximize signal transfer and ionic conduction efficiency^57^. The design space of ultrathin CNT bioelectrodes was systematically explored (Fig. 3a). The optimized CNT dry electrodes showed lower contact impedance than wet and commercial dry gold electrodes (Fig. 3b), which contributed to higher SNR in subsequent electrophysiological recordings.

A contact impedance model based on the Boolean model^58,59^ was developed and experimentally validated (Notes S2-S5). Three coupled parameters that govern contact impedance were identified as skin conformability, contact density, and sheet resistance. The first, skin conformability, is the film’s ability to adapt to the skin’s micron-scale topography (Fig. 3c). The second, contact density, quantifies the nanoscale contact points at the CNT-skin interface (Fig. 3d). The third, sheet resistance, measures the lateral electrical resistance of the film (Fig. 3e). Optical transmittance (*T*) is used as a proxy for film thickness here (Method S1). To ensure that intrinsic material properties remain constant throughout the study, thicker CNT films were obtained by stacking single-layer films of 90%*T* (Method S1).

Three distinct operational regimes for CNT thin film electrodes were observed, each governed by a different limiting parameter (Fig. 3a). With increasing film thickness or decreasing transmittance, the skin-electrode contact impedance first decreases, then increases, and eventually stabilizes in the thicker region, matching theoretical predictions.

1. Regime I: Contact Improvement (*T* > 65%). For highly transparent films, increasing thickness reduces contact impedance, driven by an exponential decrease in sheet resistance and an exponential increase in the nanoscale contact area due to increasing network density.
2. Regime II: Impedance Rebound (*T* ≈ 65–40%). Beyond the optimal point, a loss of macroscopic conformability becomes the limiting factor. Thicker films are less flexible and don’t conform well to the micro-valleys of the skin (Fig. S13), which results in higher values of contact impedance. Hence, an impedance rebound is observed.
3. Regime III: Saturation (*T* < 40%). Further reductions in sheet resistance or increases in network density provide negligible benefits in this regime. In the thickest films, the film conforms only to the broadest skin features, which are relatively flat. The contact impedance saturates.

This regime-based framework provides a fundamental understanding of the competing mechanisms governing CNT thin-film electrode performance and offers general insights into the contact impedance behavior of other porous thin films interfacing with nonplanar or textured surfaces. At its optimum (*T* ≈ 65%), the CNT electrode achieves a contact impedance of 13.7 kΩ·cm^2^ at 10 Hz, which is threefold lower than wet gel electrodes (38.8 kΩ·cm^2^). Under direct mechanical pressure (Fig. 3h), the impedance variation of CNT electrodes remained below 7.3%. The optimized film condition, *T* ≈ 65%, was used for subsequent user studies, including electrophysiological recordings and myoelectric interface experiments.

### Biocompatibility assessment

**Fig. 4:**
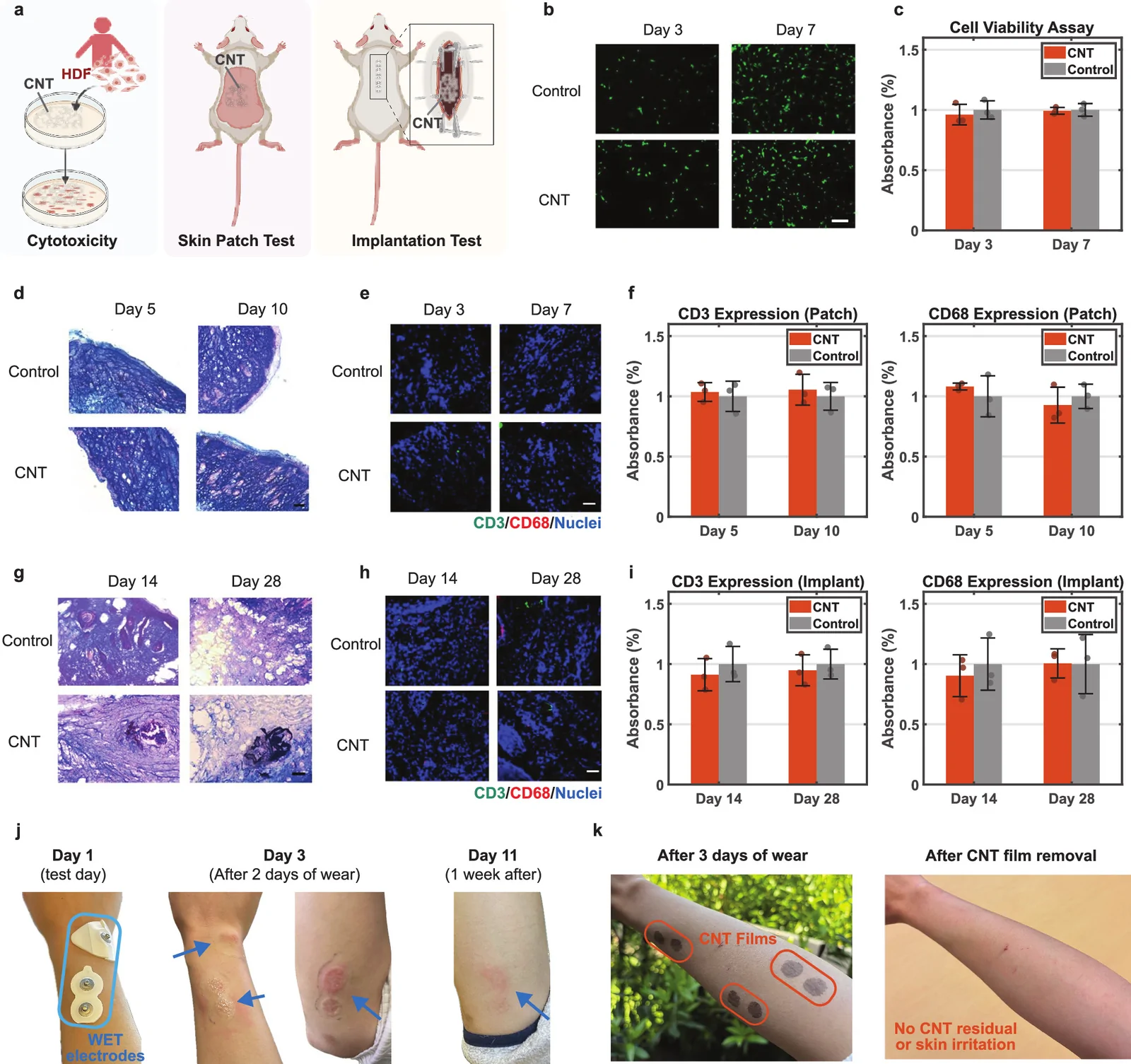
Biocompatibility of CNT films. **a** Schematic of biocompatibility assessment. Left: in vitro cytotoxicity (human dermal fibroblasts, HDFs); Middle: in vivo skin patch tests; Right: subcutaneous implantation tests in C57BL/6 mice (Created in BioRender: https://BioRender.com/44wi04a). **b**,**c** Live/Dead and MTT assays show no cytotoxicity or reduced cell viability after 3 and 7 days with no significant difference between CNT and control groups (mean ± SD, *n* = 3). Scale bar, 150 μm. **d–f** Skin patch tests show preserved tissue structure (H&E) and normal immune cell levels (IHC for CD3, CD68). No significant differences in fluorescence intensity between CNT and control groups (mean ± SD, *n* = 3). Scale bar, 100 μm. **g–i** Subcutaneous implantation shows thin fibrous capsule formation typical of a normal foreign-body response, with no abnormal CD3/CD68 expression and no significant difference between CNT and control groups (mean ± SD, *n* = 3). Scale bar, 100 μm. **j**,**k** Human tests show CNT dry electrodes cause minimal irritation compared with commercial wet electrodes, which induce visible redness and abrasion persisting up to one week.

The potential toxicity of CNTs is typically attributed to their nanoscale dimensions, particularly their ultrafine diameter and short length, which allow individual nanotubes to detach and penetrate biological membranes^60^. In contrast, the CNTs used in this work were synthesized directly as a continuous, macroscopically entangled network which eliminates free or respirable nanotube fragments. To assess the biocompatibility of the synthesized CNT films, we performed three in vitro and in vivo evaluations: cytotoxicity assays, a skin patch test, and a subcutaneous implantation test (Fig. 4a).

In vitro cytotoxicity assays showed that the films had no adverse effect on the viability or metabolic activity of human dermal fibroblasts (Fig. 4b, c). In vivo studies in mouse models further demonstrated the material’s safety. Ten-day topical skin patch tests revealed no irritation, with histology confirming normal skin architecture and baseline immune cell counts (Fig. 4d–f). Furthermore, 28-day subcutaneous implants showed only a minimal foreign body response, characterized by a thin fibrous capsule without chronic inflammation (Fig. 4g–i). Because the present tests only assess local cytotoxicity, skin irritation, and tissue response under relevant exposure conditions, they do not constitute a comprehensive systemic toxicology study of CNTs. These biocompatibility results should also be interpreted within the scope of noninvasive epidermal use on intact skin. Using CNTs in implantable or invasive devices will require more detailed evaluation of CNT migration, accumulation, and long-term biological impact.

In human subjects, the CNT films demonstrated excellent long-term skin compatibility (Fig. 4j, k). After three days of continuous wear, the CNT films caused minimal irritation. Wet electrodes, on the other hand, induced significant redness and discoloration that persisted for several days post-measurement. Skin maceration and blistering were also observed at 48 h and the wet electrodes had to be removed. These results suggest that the synthesized CNT films are more suitable for long-term wear than conventional wet electrodes.

### Electrophysiology monitoring performance

**Fig. 5:**
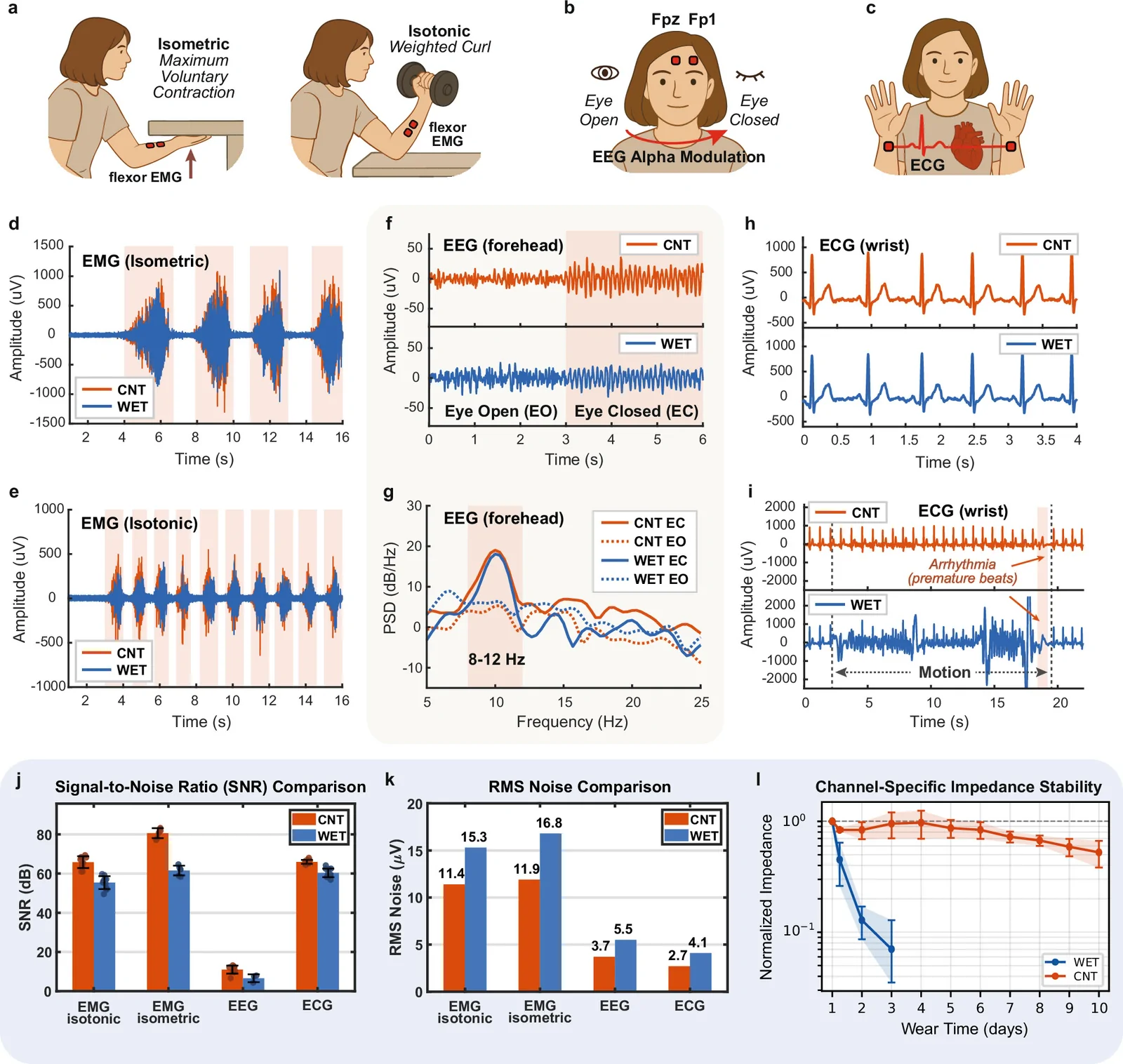
Electrophysiological performance of CNT electrodes compared to conventional wet Ag/AgCl electrodes. **a–c** Schematics of experimental setups for EMG, EEG, and ECG recordings: isometric and isotonic contractions (**a**), forehead EEG at Fp1–Fpz for alpha modulation (**b**), and wrist ECG (**c**). **d**,**e** Representative raw EMG signals from CNT (orange) and wet Ag/AgCl (blue) electrodes during isometric (**d**) and isotonic (**e**) contractions. **f**,**g** EEG recordings exhibit clear alpha-band (8–12 Hz) modulation between eyes open (EO) and closed (EC) states for both electrodes. **h** Synchronized ECG recordings on wrists showing both CNT and wet electrodes display clear P-QRS-T waves. **i** ECG traces show CNT electrodes maintain signal fidelity during motion and can detect arrhythmia events, unlike wet electrodes. **j**,**k** Summary bar charts comparing the mean ± SD SNR (**j**) and mean RMS noise (**k**) for CNT and wet electrodes across all four recording modalities: isotonic EMG (*n* = 10), isometric EMG (*n* = 6), EEG (*n* = 3), and ECG (*n* = 12). **l** Channel-specific contact impedance stability (normalized to initial application) for CNT (*n* = 10) and wet (*n* = 8) electrodes (mean ± SD).

To assess signal fidelity, the optimized CNT electrodes (65%*T*) were benchmarked against gold standard wet electrodes across three clinically relevant electrophysiological modalities (Fig. 5a–c): electromyography (EMG), electroencephalography (EEG), and electrocardiography (ECG).

In EMG recordings from the forearm, the CNT electrodes achieved a high SNR during both dynamic, isotonic contractions (65.8 ± 3.0 dB vs. 55.4 ± 3.3 dB, *p* < 0.001) and static, isometric contractions (80.6 ± 2.6 dB vs. 61.6 ± 2.5 dB, *p* < 0.001) compared to wet electrodes (Fig. 5d, e). In a standard eyes-open/closed EEG recording task, the CNT electrodes captured the alpha peak from the forehead with a higher contrast-to-noise ratio (CNR) (11.0 dB vs. 6.6 dB, *p* < 0.05) (Fig. 5f,g). During ECG recordings from the wrist (Fig. 5h), while both electrode types performed well at rest (65.97 ± 2.17 dB vs. 59.76 ± 2.96 dB, *p* < 0.001), motion interference made the wet electrode signals uninterpretable, and critical arrhythmia events were unidentifiable. On the other hand, the CNT electrodes maintained the shape of P-QRS-T waves, and the premature beats were distinct (Fig. 5i). This resilience to motion artifacts is key to ambulatory, real-world applications.

Overall, the CNT electrodes demonstrated high SNR, low noise, and reduced motion artifacts (Fig. 5j, k). Additional contact impedance measurements over 10 days also showed less channel-specific variation compared to the wet electrodes (Fig. 5l). The improved signal quality and inter-channel stability are critical for downstream ML decoding, where stable relative EMG amplitudes across channels are needed to preserve decoder performance over time.

### Multi-day decoder stability in myoelectric control

**Fig. 6:**
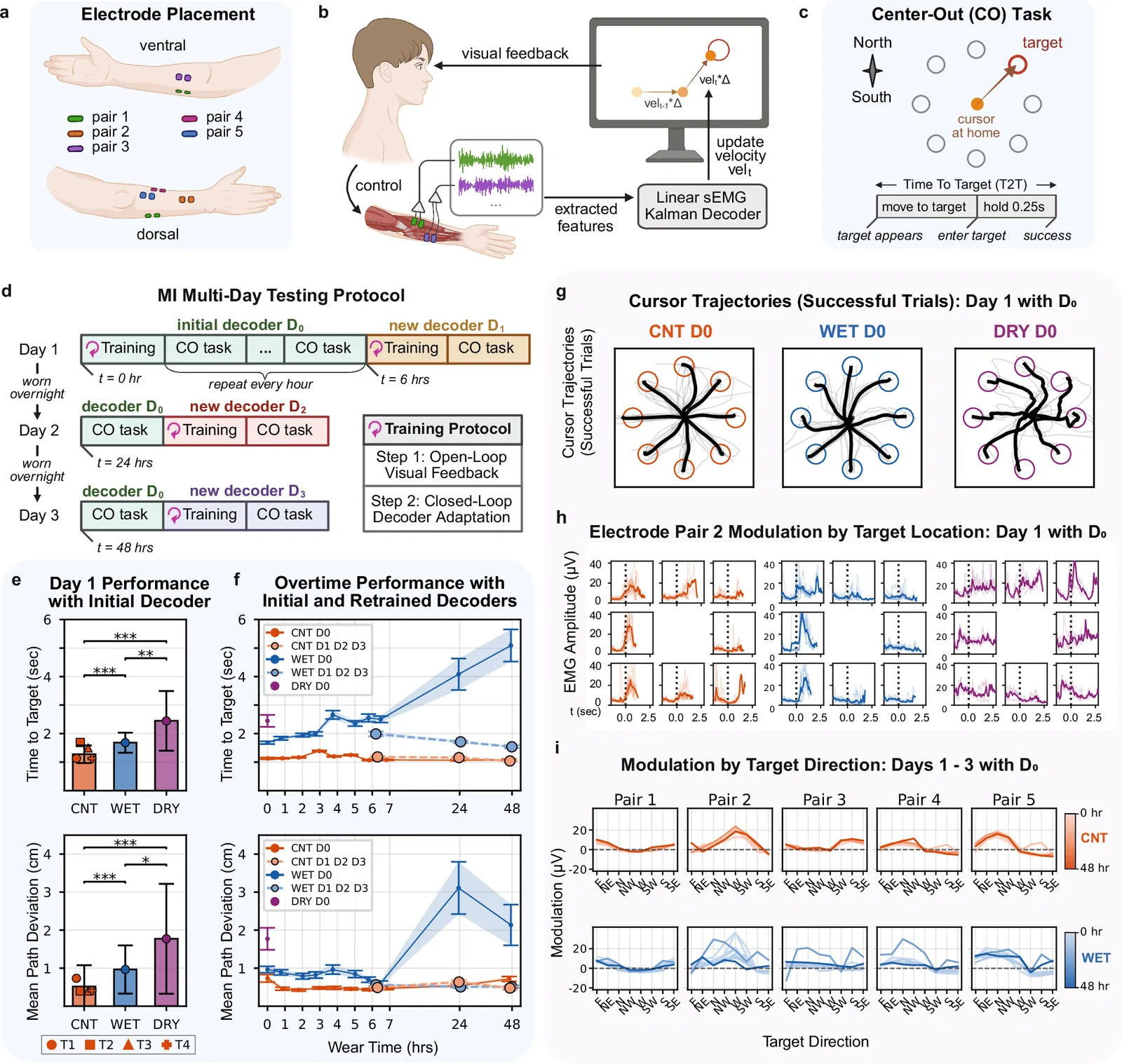
CNT electrodes in an EMG-driven myoelectric interface. **a** Placement of CNT, Ag/AgCl wet, and gold dry electrode pairs on the forearm. **b** Schematic of the EMG-driven MI with a Kalman filter decoder. **c** Center-out task with eight radial targets. **d** Multi-day protocol: initial decoder trained on Day 1, reused for 48 h without retraining; additional decoders trained at 6, 24, and 48 h. **e** Baseline time-to-target (T2T) and mean path deviation (MPD) across CNT (*n* = 219), wet (*n* = 56), and dry (*n* = 25) electrodes (mean ± SD). T1, T2, T3, and T4 represent four separate sessions, each using a new set of CNT electrodes. Two-sided Welch’s t-tests were used. T2T: CNT vs. wet, *p* = 1.61 × 10⁻^11^ (***); wet vs. dry, *p* = 0.00142 (**); CNT vs. dry, *p* = 9.64 × 10⁻⁶ (***). MPD: CNT vs. wet, *p* = 6.81 × 10⁻⁶ (***); wet vs. dry, *p* = 0.0122 (*); CNT vs. dry, *p* = 2.30 × 10⁻⁴ (***). **f** T2T and MPD over 48 h with initial (solid lines) and retrained (dashed lines) decoders: CNT (orange), wet (blue), dry (purple). Data are presented as mean ± SEM. Exact n values are provided in the Methods (CNT: *n* = 71–84; wet: *n* = 19–58; dry: *n* = 25). **g** Representative cursor trajectories at baseline. Gray traces indicate individual successful trials; black traces indicate average trajectories. **h** Peristimulus time histograms (PSTHs) from electrode pair 2 at baseline. Dashed line indicates target appearance. **i** Tuning stability over 48 h for CNT (orange) and wet (blue) electrodes showing modulation amplitude for each direction: wet electrodes display reduced amplitude and distorted directional tuning, whereas CNT electrodes preserve both amplitude and specificity over time. Figures 6a–d were created in BioRender: https://BioRender.com/0goxw56.

Myoelectric interfaces offer a powerful tool in neurorehabilitation and in human-computer interaction^61–64^, but electrode instability and the constant need to recalibrate limit the potential of these devices. To demonstrate that the CNT electrodes overcome this limitation, a myoelectric interface (MI) for 2D computer cursor control was implemented and worn continuously for multiple days, without removal or reapplication. Five pairs of CNT electrodes were placed along the forearm to capture muscle activity underlying wrist flexion/extension and abduction/adduction (Fig. 6a). A Kalman filter decoder^65,66^ predicted cursor velocity from the filtered root-mean-square of five electrode pair signals, using less than 5 min of training data (Fig. 6b).

First, the user was tasked with controlling the cursor from the home position toward one of eight radial targets as it appeared on the screen (Fig. 6c). An initial decoder was trained once on Day 1 (decoder D_0_) and then tested during the remaining wear time without any retraining. Additional decoders (D_1_-D_3_) were trained at 6 h, 24 h, and 48 h for comparison (Fig. 6d). Two performance metrics were used to benchmark against commercial Ag/AgCl wet and gold dry electrodes: time-to-target (T2T) and mean path deviation (MPD). T2T was defined as the time taken for the cursor to move from the home position to the target and successfully complete the trial. MPD was defined as the lateral distance between the cursor trajectory and the shortest path (a straight line from the home position to the target). Lower T2T and MPD values indicate more efficient and stable cursor movement. If the user failed to reach the target within 10 s, the attempt was considered failed.

Baseline performance on Day 1 using the initial decoder was highest for CNT electrodes, with shorter T2T (CNT 1.28 s vs. wet 1.68 s vs. dry 2.45 s; *p* < 0.001) and smaller MPD values (CNT 0.52 cm vs. wet 0.97 cm vs. dry 1.77 cm; *p* < 0.001) than wet or dry electrodes (Fig. 6e). Multi-day MI testing (Fig. 6f, Movies S2-S3) demonstrated that the CNT electrodes showed stable T2T and MPD over 48 h with a 100% success rate, using D_0_ without retraining. On the other hand, T2T and MPD worsened with wet electrodes and D_0_ at 24 h and 48 h and the success rate fell to 86%. When wearing CNT electrodes, retrained decoders at 6 h (D_1_), 24 h (D_2_), or 48 h (D_3_) yielded no improvement above performance with D_0_ at the same time points, indicating that the CNT electrodes enabled stable signal quality. In contrast, wet electrodes showed performance recovery with the retrained decoders (D_1_ T2T 5.09 s vs. D_3_ T2T 1.54 s), and the success rate improved to 100%. Such recovery after retraining indicated that instability of the wet electrodes rather than material degradation alone was key to decoder performance. Across individual successful trials, the cursor trajectories with CNT and wet electrodes were both fairly target-directed, while more erratic and variable paths were observed using dry electrodes on Day 1 (Fig. 6g). Filtered RMS signals from each electrode pair demonstrate that the CNT and wet electrodes show direction-modulated patterns, whereas the dry electrodes fail to preserve the distinct direction-specific patterns (Fig. 6h). Over 48 h, CNT electrodes preserved both signal amplitude and tuning, whereas gel dehydration in wet electrodes rendered the decoder weights unreliable, resulting in amplitude loss and/or tuning distortion which requires retraining to recover (Fig. 6i, Fig. S18).

**Fig. 7:**
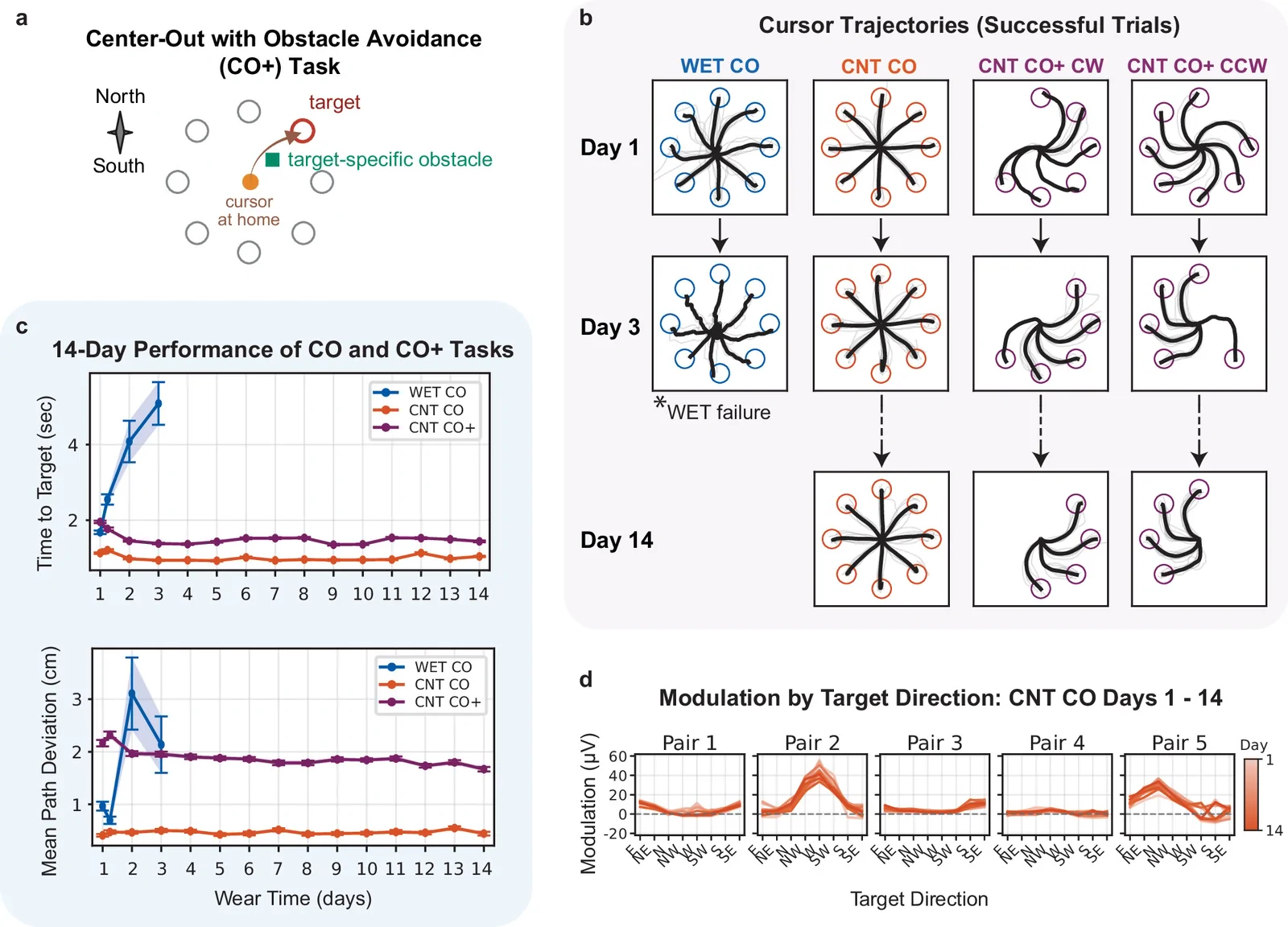
CNT myoelectric interface stability under extended wear and increased task complexity. **a** Schematic of the center-out with obstacle avoidance (CO + ) task. **b** Representative cursor trajectories from successful trials for wet CO task, CNT CO task, and CNT CO+ task in both clockwise (CW) and counterclockwise (CCW) directions on Day 1 (wet CO: *n* = 56; CNT CO: *n* = 66; CNT CO + : *n* = 58), Day 3 (wet CO: *n* = 19; CNT CO: *n* = 79; CNT CO + : *n* = 72), and Day 14 (CNT CO: *n* = 75; CNT CO + : *n* = 70) respectively. Gray traces indicate individual successful trials; black traces indicate average trajectories. **c** Long-term CNT myoelectric interface performance over 14 days for CNT CO and CO+ task: time to target (top) and mean path deviation (bottom).). Data are presented as mean ± SEM. Exact n values are provided in the Methods (CNT CO: *n* = 62–81; CNT CO + : *n* = 58–74; wet CO: *n* = 19–56). **d** Tuning stability across 14 days for CNT electrodes showing preserved modulation amplitude and specificity for each direction over time.

To further stress test long-term interface stability, we extended the CNT MI study to 14 days and introduced a more challenging center-out task with obstacle avoidance (CO+ ) (Fig. 7a). In the CO+ task, the user had to navigate the cursor around a target-specific obstacle, requiring finer control of cursor direction and velocity than the standard center-out task. The user could reach each target through either a clockwise (CW) or a counterclockwise (CCW) trajectory. The same initial decoder for the CO task was used for the CO+ task. Throughout the 14 days of continuous wear, CNT electrodes maintained the myoelectric interface performance using the same initially trained decoder. The cursor trajectories were clean and efficient in the CO task and in both clockwise (CW) and counterclockwise (CCW) CO+ tasks (Fig. 7b). Both T2T and MPD remained stable, with the CO+ task showing slightly longer completion times and larger path deviations than the standard CO task due to the increased task complexity (Fig. 7c). Direction-specific EMG modulation patterns were also preserved across 14 days (Fig. 7d, Fig. S19) with minimal drift (Fig. S20). These results showed that CNT electrodes maintained stable multichannel muscle activation features needed for recalibration-free decoding. In addition, cross-session decoder transfer was evaluated by applying a decoder from a previous session to a new set of CNT electrodes, placed at the same forearm locations (Fig. S22). The transferred decoder showed similar CO and CO+ performance as the newly trained decoder (Figs. S23, S24), suggesting that newly applied CNT electrodes maintain stable skin interfaces across sessions.

These results highlight that instability of traditional wet electrodes remains the primary barrier to long-term ML-integrated wearable systems. As a result, most current ML pipelines assume a stationary input distribution and rely on periodic recalibration to account for slow signal drift or abrupt disruptions. In this work, the CNT electrodes required no retraining to maintain reliable task performance due to their interface stability. In addition, less than 5 min of user-specific training data were needed to train the initial decoder, making the system viable for users with movement impairments, who may fall outside the training data distribution for generalized cross-subject cross-session models. By sustaining high performance without recalibration, CNT electrodes reduce the retraining burden on the user, enable user learning over time, and allow comfortable and uninterrupted multi-day operation in naturalistic settings.

## Discussion

This work demonstrated high-fidelity, stable dry electrodes based on CNT thin film in a multi-day EMG-based myoelectric interface task. Using a Kalman filter decoder trained with limited user-specific data only upon initial application, the system outperforms the clinical-standard wet electrodes and maintains consistent performance over 14 days of continuous wear without retraining. These results address long-standing challenges of wet electrodes, which are susceptible to signal drift and frequent decoder updates due to interface instability. Operating without skin preparation or conductive gels, the CNT electrodes show a flexible, breathable, and biocompatible form factor that maintains compliant and stable contact with the skin during extended wear. Through systematic model development and impedance characterization, three key design axes governing skin–electrode interfaces were identified: conformability, contact density, and sheet resistance. These parameters define a design trade-off framework applicable to electrodes composed of percolated networks across a broad range of materials. Balancing these properties results in stable, low contact impedance, high signal-to-noise ratio (SNR), and reduced motion-induced artifacts under naturalistic conditions, exceeding the performance of clinical-standard wet gel electrodes. At the system level, our results indicate that, in machine-learning–integrated bioelectronic systems, robust long-term performance depends critically on the engineering of interfacial properties that preserve signal integrity over time. The CNT electrodes support recalibration-free inference in a myoelectric interface, substantially reducing system complexity, user burden, and cognitive load. These findings establish CNT thin films as a promising platform for scalable long-term bioelectronic interfaces for ambulatory applications.

Several limitations remain. The myoelectric decoding experiments were designed as pilot demonstrations: larger-cohort user studies, longer-term protocols, and clinical trials will be needed to establish population-level generalizability, particularly in rehabilitation populations. Future work should evaluate performance across diverse user characteristics, including skin type and condition, sweating response, muscle strength, motor-unit recruitment, and fatigue. The impact of these factors should be quantified through inter- and intra-user variation in electrode–skin impedance, signal stability, decoding accuracy, and calibration burden. For users with impaired neuromuscular control, multi-month clinical trials are needed to evaluate whether stable CNT electrode interfaces translate into improved physical therapy and neurorehabilitation outcomes, quantified by longitudinal changes in motor function, therapy adherence, and functional recovery. Although the CNT electrodes maintained signal quality after exercise, sweating, and shower exposure (Fig. S16), decoder stability was not evaluated during more extreme conditions, such as underwater exposure or active heavy sweating. Future work should assess continuous multichannel recording and ML decoding during and immediately after wet or high-motion conditions. These studies will help identify the mechanisms underlying performance degradation under challenging real-world conditions and guide optimization strategies for the electrode interface properties, such as thermal transport characteristics, underwater encapsulation, and adhesion stability, without compromising breathability. Lastly, the practical lifetime of the current device is limited by device-level integration, including interconnect fatigue, substrate degradation, and gradual changes at the skin surface. Future development of high-performance flexible encapsulation and interconnect layers will be needed to realize a fully integrated long-term wearable device.

## Methods

### CNT film synthesis

Carbon nanotube (CNT) films were synthesized in a floating catalyst chemical vapor deposition (FCCVD) furnace comprising a high-temperature reactor (1000–1200 °C)^23^. The feedstock contained ferrocene (FeCp_2_) as a catalyst precursor, sulfur as a growth promoter, and methane (CH_4_) as the carbon source. Nitrogen (N_2_) and hydrogen (H_2_) served as carrier gases to promote CNT synthesis and aerogel formation. The resulting CNT network was continuously collected as a free-standing film onto a low-surface-energy substrate using a roll-to-roll winding system. Film transmittance, thickness, and sheet resistance were controlled by tuning growth conditions. The temporary collection substrate was selected to prevent film collapse, folding, or twisting while enabling subsequent release and patterning (Table S6).

### Characterization of CNT films

Material characterization was performed as follows. Surface morphology was analyzed using a field-emission scanning electron microscope (FE-SEM, Zeiss Gemini 500) at an accelerating voltage of 5.0 kV. High-resolution structural and crystalline analysis was conducted on a High-Resolution Transmission Electron Microscope (HRTEM, FEI Tecnai G2 F20 X-TWIN). The CNT thin films were press-transferred onto Cu grids as free-standing films during electron microscopy. TEM analysis was performed on 3 samples for each synthesis condition. A confocal Raman spectrometer (Horiba LabRAM) was used to assess CNT quality, and a 532 nm laser excitation source was used. Raman spectra analysis was performed on 3 samples for each synthesis condition. Sheet resistance was measured by a four-point probe (RM3000 + , Jandel Engineering Ltd). Optical transmittance of the films was measured with a spectrophotometer (Agilent Cary 5000 UV-Vis-NIR) at a wavelength of 550 nm. For sheet resistance and optical transmittance, 3 locations were measured on each sample, and 3 samples were collected for each synthesis condition. CNT films on skin replicas were sputter-coated with ~5 nm Au/Pd prior to SEM imaging. For each CNT film thickness, SEM analysis was performed on 3 samples. Skin replicas were prepared using a two-step molding process. First, Dragon Skin 10 silicone elastomer (Smooth-On, Inc) was cast directly on the human forearm to capture the microscale skin texture. After curing, the Dragon Skin negative mold was gently peeled from the skin. UV-curable resin was then cast onto the Dragon Skin mold and cured under UV illumination to generate a positive replica of the skin surface. Breathability was quantified by water vapor transmission rate (WVTR) measurements using a gravimetric method (*n* = 3 for each CNT film thickness). CNT films of various thicknesses were sealed over scintillator vials containing deionized water, and mass loss was monitored in an ambient lab environment (22 ± 1 °C and 40 ± 5 % relative humidity). An open chamber served as the 100 % transmission reference. Mechanical durability was assessed by two cyclic tests (one sample per test condition): (1) tensile testing, in which films were stretched to 30% strain for 2000 cycles; (2) bending testing, in which films were bent to a radius of 3 mm for 1000 cycles. Resistance was continuously recorded with a Keysight 34470 A in the four-wire mode to eliminate contact artifacts. Electrochemical stability was characterized by immersing CNT films in artificial sweat (phosphate-buffered saline, Sigma-Aldrich) at 37 °C for four weeks, with periodic electrochemical impedance spectroscopy measurements (Ivium-n-Stat, Ivium Technologies) to monitor impedance changes.

### Electrode fabrication

Tattoo-style CNT dry electrodes (Figure S15) were fabricated on commercial temporary tattoo paper (Silhouette America, Inc., USA). Silver (Ag) interconnects (Novacentrix PSPI-1000) were patterned through a laser-cut 5 µm polyimide mask and sintered at 70 °C for 20 min. The mask’s central region was removed, and a CNT film was press-transferred from the roll-to-roll substrate onto the tattoo paper, due to the surface energy preference. Ethanol spraying was used to densify the CNT network and reduce junction resistance. Films were used pristine (without additional chemical doping) to ensure long-term stability and biocompatibility. For skin application, the electrode was placed at the desired site and the backing layer wetted to dissolve the water-soluble release polymer. As the water evaporated, van der Waals interactions promoted conformal adhesion to the skin. The skin was fully dried before measurements were taken.

### Biocompatibility assessment

Biocompatibility of the CNT films was evaluated following ISO 10993 standards through a combination of in vitro cytotoxicity, in vivo skin patch, and subcutaneous implantation tests. Anti-CD3 [SP7] (ab16669) and anti-CD68 (ab31630) were purchased from Abcam (Cambridge, UK) and used for immunostaining, with both antibodies diluted 1:100 in the final staining solution. Normal adult human dermal fibroblasts (HDF) from Lonza (Basel, Switzerland) were used for in vitro biocompatibility assessment. C57BL/6 wild-type male mice were obtained from Jackson Laboratory (Bar Harbor, ME, USA). Mice were housed under specific-pathogen-free conditions in the animal facilities at the California Institute of Technology. Animals were maintained on a 12-h light/12-h dark cycle at an ambient temperature of approximately 22 ± 2 °C and relative humidity of 40–60%. Mice were housed in standard laboratory cages with appropriate bedding and environmental enrichment and had ad libitum access to standard laboratory chow and water. Mice used for all experiments were approximately 8 weeks of age.

For cytotoxicity assessment, UV-sterilized 1 × 1 cm CNT films were co-cultured with human dermal fibroblasts (HDFs) at 37 °C and 5% CO₂, with culture medium as a negative control. Cell viability and metabolic activity were quantified after 3 and 7 days using Live/Dead staining and MTT assays. For skin patch testing, 0.5 cm × 0.3 cm CNT films on polymer substrates were applied to the shaved dorsal skin of C57BL/6 mice for 5 and 10 days, after which tissue samples were collected for hematoxylin–eosin (H&E) staining and immunohistochemistry (IHC) for CD3 and CD68. Polymer substrate alone served as the control. For implantation testing, 3 cm × 3 cm CNT films were inserted into dorsal skin pockets of C57BL/6 mice, with sham surgeries serving as controls. Incision sites were sutured and allowed to heal normally. Samples were explanted after 14 and 28 days and examined histologically (H&E and CD3/CD68 IHC) to evaluate tissue integration and immune response. Statistics were taken two-sided.

### Human subject testing

Five healthy adult subjects of both sexes, aged 18–40 years, were recruited for this study. Skin was cleaned with 70% isopropanol prior to electrode placement; no abrasion or shaving was performed. CNT electrodes were compared against commercial Ag/AgCl wet electrodes (Duotrode™ and 3 M Red Dot™) and Au-coated dry electrodes (Dryode™) of matched area. Skin at electrode sites was visually inspected after each session and monitored for up to 27 days post-use for irritation, blistering, or discoloration.

### Impedance measurements

Electrode–skin impedance was measured using an Impedance Analyzer (IM3536, Hioki, Japan). Frequency sweeps (10 Hz–1 MHz, 40 points per decade) were performed after application of the electrodes on the human skin. Measurements were taken from distinct samples.

### Electrophysiological recordings

EEG, EMG, and ECG recordings were acquired using CNT-based electrodes placed in parallel with reference wet electrodes on the same subject. EEG electrodes were positioned at Fp1/Fp2 following the international 10–20 system, with subjects alternating between 30-s eyes-open and eyes-closed epochs in a quiet room. EMG electrodes were placed over the dominant forearm flexor muscle with 2 cm spacing as subjects performed isometric (3-s static crimp using a spring tool) and isotonic (3-lb dumbbell curl) contractions. ECG electrodes were applied to the wrists in a Lead-I configuration and recorded during rest and during repetitive wrist movements and gentle tapping next to the electrode to introduce motion artifacts. EEG signals were bandpass filtered between 5–50 Hz; EMG, 5–100 Hz; and ECG, 1–50 Hz. All signals were notch-filtered at 60 Hz, and EEG spectral features were further analyzed using Welch’s method to extract alpha-band (8–12 Hz) power and signal quality metrics. Data were processed using MATLAB (version R2020b).

### ML inference stability testing

Five pairs of surface electrodes were placed on the right forearm targeting major muscle groups: pronator teres (pair 1), extensor pollicis longus/brevis (pair 2), flexor carpi (pair 3), extensor carpi (pair 4), and extensor digitorum (pair 5). EMG signals were acquired at 1 kHz sampling rate and 28-bit resolution using a bioamplifier and data acquisition system (RZ2 BioAmp Processor with SIM Subject Interface Module, TDT, USA) controlled via a WS8 workstation running Synapse software. Raw EMG data were band-pass filtered between 10 Hz and 100 Hz and notch-filtered at 60 Hz and 120 Hz (20 dB, 1/10-octave bandwidth). The root-mean-square (RMS) envelope was extracted using a 3 Hz low-pass smoothing filter and streamed in real time at 1 kHz to the decoder. A Kalman filter decoder was trained using a closed-loop decoder adaptation (CLDA) protocol consisting of (i) open-loop training with visual cursor feedback and (ii) assisted closed-loop control with gradually reduced computer assistance. The final decoder was evaluated in a two-dimensional center-out task with eight radial targets. CNT electrodes were benchmarked against commercial Ag/AgCl wet electrodes (Duotrode™, Myotronics, USA) and Au-coated dry electrodes (Dryode™, NeuroTek Inc., USA) of matched surface area. Four independent sessions were conducted (Fig. S21), each using a newly fabricated set of 10 CNT electrodes placed in the same forearm configuration. Three sessions were performed over 48 h, and one extended session was performed over 14 days. Data were processed using Python (version 3.13.5) and statistics were taken two-sided using Welch’s t-tests.

For task-performance analysis, time-to-target (T2T) and mean perpendicular deviation (MPD) are reported as mean ± SEM across repeated task trials within each session. For T2T, *n* represents the number of successful attempts in which the target was reached within 10 s. For MPD, *n* represents the total number of trials, including both successful and unsuccessful attempts. Over the duration of wear, individual task trials are repeated measurements from the same participant.

For T2T with the initial CNT decoder (D0), the numbers of successful attempts at 0, 1, 2, 3, 4, 5, 6, 7, 24, and 48 h were *n* = 76, 75, 76, 71, 78, 72, 79, 84, 82, and 81, respectively. For the initial wet decoder (D0) at the corresponding time points, *n* = 56, 50, 51, 48, 37, 43, 39, 40, 21, and 19, respectively. For the retrained CNT decoders (D1–D3) at 6, 24, and 48 h, *n* = 79, 78, and 82, respectively, and for the retrained wet decoders, *n* = 51, 55, and 58, respectively. For the dry-electrode condition at 0 h, *n* = 25.

For MPD with the initial decoder, the number of trials for CNT electrodes at 0, 1, 2, 3, 4, 5, 6, 7, 24, and 48 h was *n* = 76, 75, 76, 71, 78, 72, 79, 84, 82, and 81, respectively. For wet electrodes at the corresponding time points, *n* = 56, 50, 51, 48, 37, 43, 39, 40, 26, and 22, respectively. For the retrained CNT decoder at 6, 24, and 48 h, *n* = 79, 78, and 82, respectively; for the retrained wet decoder, *n* = 51, 55, and 58, respectively. For the dry-electrode condition at 0 h, *n* = 25.

The same method applies to the obstacle avoidance task during extended wear. For CNT electrodes in the center-out (CO) task, the number of successful attempts included in the T2T analysis at 0, 6, 24, 48, 72, 96, 120, 144, 168, 192, 216, 240, 264, 288, and 312 h was *n* = 66, 62, 75, 79, 79, 81, 77, 79, 76, 79, 77, 78, 70, 76, and 75, respectively. For the CNT obstacle-avoidance (CO + ) task at the corresponding time points, *n* = 58, 64, 70, 72, 73, 71, 69, 68, 64, 73, 74, 70, 68, 70, and 70, respectively. For wet electrodes in the CO task at 0, 6, 24, and 48 h, the number of successful attempts included in the T2T analysis was *n* = 56, 39, 21, and 19, respectively.

For MPD, the total number of trials for CNT electrodes in the CO task at 0, 6, 24, 48, 72, 96, 120, 144, 168, 192, 216, 240, 264, 288, and 312 h was n = 66, 62, 75, 79, 79, 81, 77, 79, 76, 79, 77, 78, 70, 76, and 75, respectively. For the CNT CO+ task at the corresponding time points, *n* = 58, 64, 70, 72, 73, 71, 69, 68, 64, 73, 74, 70, 68, 70, and 70, respectively. For wet electrodes in the CO task at 0, 6, 24, and 48 h, the total number of trials included in the MPD analysis was *n* = 56, 39, 26, and 22, respectively.

### Ethics declarations

All animal experiments were conducted in accordance with institutional guidelines for the care and use of laboratory animals and were approved by the Institutional Animal Care and Use Committee (IACUC) of the California Institute of Technology (Caltech) under an approved animal protocol. All human studies were approved by the University of California, Berkeley Institutional Review Board (IRB). Each participant gave informed written consent.

### Reporting summary

Further information on research design is available in the Nature Portfolio Reporting Summary linked to this article.

## Supplementary information

Supplementary Information

Description of Additional Supplementary Files

Supplementary Movie 1

Supplementary Movie 2

Supplementary Movie 3

Reporting Summary

Transparent Peer Review file

## Source data

Source Data

## Data Availability

All data supporting the findings of this study are available within the article and its supplementary files. Any additional requests for information can be directed to, and will be fulfilled by, the corresponding authors. Source data are provided with this paper. Raw EMG data for multiday myoelectric cursor control are available at https://doi.org/10.5281/zenodo.22071769. Source data are provided with this paper.

## Code availability

Code used for data analysis is available at https://doi.org/10.5281/zenodo.22071769.
