# Supplementary material for: Multi-day recalibration-free electrophysiology interface based on carbon nanotube thin film dry electrodes
Source: Nat Commun. 2026 Oct 13;17:11583. doi: 10.1038/s41467-026-78169-6 (PMC13751614; doi:10.1038/s41467-026-78169-6)
Supplement: Supplementary file 1 [file 41467_2026_78169_MOESM1_ESM.pdf]

# Supplementary information

## Multi-day Recalibration-free Electrophysiology Interface Based on Carbon Nanotube Thin Film Dry Electrodes

Supplementary Figures S1-S24

Supplementary Tables S1-S7

Supplementary Notes S1-S5

Supplementary Methods S1

Supplementary Movies S1-S3

**Supplementary Table S1 |** Comparison of key properties across wet, dry, and CNT-based electrodes for long-term, ML-compatible biosignal acquisition.

| Challenges | Wet Gel Electrodes | Conventional Dry Electrodes | CNT Dry Electrodes (This Work) |
|---|---|---|---|
| **Skin preparation & stability** | Requires abrasive skin prep and gel application; risk of channel shorting | Quick setup, but requires frequent repositioning; long settling time | **Gel-free, maintenance-free setup; stable contact from initial application** |
| **Contact impedance** | Low initially, but increases with gel drying | High and sensitive to motion and environment | **Low and stable overtime without conductive gel** |
| **Signal fidelity (SNR)** | High at first, but degrades as gel dries and motion disrupts gel-skin contact | Moderate and inconsistent; susceptible to signal dropout | **High SNR maintained over multi-day wear** |
| **Motion artifact resistance** | Moderate initially; worsens as gel redistributes | Poor; air gaps and rigid interfaces amplify artifacts | **Strong resistance due to ultrathin, flexible, skin-conformal design** |
| **Biocompatibility** | Limited by gel and adhesive, irritation, poor breathability | Better than wet, but rigidity causes mechanical stress and discomfort | **Excellent: soft, porous, breathable, and irritation-free** |
| **User Experience & Perceptibility** | Bulky and uncomfortable; hinders natural movement | Often stiff; relies on pressure for contact | **Nanometers-thin, van der Waals adhesion; imperceptible on skin** |
| **ML retraining burden** | Frequent retraining is needed due to impedance drift and motion artifacts | Long settling time; inter-session variability, frequent recalibration | **Stable model performance over 48 hours without retraining** |
| **Suitability for real-world use cases** | Impractical for long-term use due to drying, irritation, and maintenance needs | Signal instability limits use in fine-resolution and extended applications | **Enables robust, multi-day ML-driven monitoring in naturalistic settings** |

**Supplementary Table S2 |** Performance comparison of nanomaterial dry electrophysiology electrodes.

| Material class | ref # | Material System | Form Factor | \|Z\| kΩ·cm² | Signal (vs. wet) | Mechanical Stability | Motion Noise | Breathability ($g·m^{-2}·h^{-1}$) | Extended Wear | ML Stability |
|---|---|---|---|---|---|---|---|---|---|---|
| **MXene** | 1 | MXene/CNF/PCE on fabric | 3D Textile | 1350 (100 Hz) | ECG ↓ SNR | 15% strain; 1000 bends | - | Intrinsic (structural) | - | 96% accuracy; Stability - |
| | 2 | MXene/cellulose-polyester | Mini-pillar electrode | 0.35 (1 kHz) | ECG/EMG/EEG ≈ visual | - | ↓ | - | - | - |
| **Metal Nano-material** | 3 | AgNW/PU/HPAN | Textile patch | ~2000 (10 Hz) | ECG/EMG ≈ SNR | 500% strain 4200 bends (3 mm) | - | 72.8 | Stable under sweat | - |
| | 4 | Au/AuNP/PI | Flexible patch | - | ECG/EMG ↑ SNR | 10,000 bends (5 mm) | ↓ | - | - | - |
| **Graphene** | 5 | MLG/SEBS | E-tattoo | ~20 (10 Hz) | ECG/EMG/EEG ≈ visual | strain, peel-off, stir | - | - | - | - |
| | 6 | MLG | E-tattoo | ~90 (100 Hz) | EDA ≈ | 42% strain | - | - | 15 h | - |
| **CNT** | 7 | MWCNT on yarn | Textile patch | 130 (100 Hz) | ECG/EMG ≈ SNR | 70-100% strain | ↑ | Intrinsic (structural) | - | - |
| | 8 | MWCNT/PDMS | Micropillar electrode | 273 (30 Hz) | ECG ≈ visual | - | ≈ | - | 48 h (8 h settle) | - |
| | 9 | MWCNT/PDMS | Soft patch | ~900 (100 Hz) | ECG ≈ visual | 45% strain | ≈ | - | 7 days | - |
| | 10 | SWCNT/SNF | E-tattoo | - | ECG/EMG ≈ visual | 1000 stretches, compression, twist | - | Intrinsic (structural) | Stable under sweat | - |
| **This Work (CNT)** | | **FWCNT** | **E-tattoo** | **13 (10 Hz)** | **ECG/EMG/EEG ↑ SNR ↓ noise** | **2,000 cycles (30%) 1,000 bends (3 mm)** | ↓ | **49** | **14 days** | **Better than wet; Stable over 2 wks** |

**Notes:**

*Reported skin-electrode contact impedance (|Z|) are taken at the specified frequency and normalized by electrode area, assuming two electrodes.

*Signal quality vs. wet Ag/AgCl electrodes are reported based on metrics provided in the referenced works, including signal-to-noise ratio (SNR), RMS noise, or qualitative visual comparison of waveform fidelity.

*Compared to wet Ag/AgCl electrodes, ↑, ≈, and ↓ indicate lower, comparable, or higher values respectively, and - indicates not reported.

*Breathability is reported either quantitatively via water vapor transmission rate (WVTR), when available, or qualitatively based on the electrodes' intrinsic form factor and physical structure. - indicates not reported.

**Supplementary Table S3 |** Performance comparison of continuous conductive film dry electrophysiology electrodes.

| Material class | ref # | Material System | Form Factor | \|Z\| kΩ·cm² | Signal (vs. wet) | Mechanical Stability | Motion Noise | Breathability ($g \cdot m^{-2} \cdot h^{-1}$) | Extended Wear | ML Metrics & Stability |
|---|---|---|---|---|---|---|---|---|---|---|
| Organic | 11 | PEDOT:PSS | E-tattoo | - | ECG<br>↑ SNR ↓ noise | 10% strain | - | - | - | - |
| | 12 | PEDOT:PSS/ WPU/sorbitol | Self-adhesive patch | 82<br>(10 Hz) | ECG<br>≈ visual ≈ noise | 43% strain;<br>440 cycles (30%) | ↓ | - | 16 h | - |
| | 13 | PEDOT:PSS | E-tattoo | ~1600<br>(10 Hz) | ECG<br>≈ visual | - | - | - | - | - |
| | 14 | HPMC/PEO/ PEDOT:PSS | E-tattoo | 80<br>(10 Hz) | ECG/EMG<br>↑ SNR | 1000 bends (1.5 cm) | ↓ | 57 | 24 h | - |
| | 15 | PEDOT:PSS/ GO/PEGDMA | E-tattoo | ~20<br>(10 Hz) | EMG/EEG<br>↑ SNR | 110% strain<br>2000 cycles (30%) | ↓ | - | - | 92% accuracy;<br>Stability - |
| Metal | 16 | Ag/Ti on PET yarn | Textile patch | - | ECG<br>≈ visual | - | ≈ | Intrinsic (structural) | - | - |
| | 17 | Au/Cr | E-tattoo | - | ECG<br>≈ noise | 18% strain | ↓ | Breathable (physiology) | - | 97.4% accuracy;<br>Stability - |
| | 18 | Au/SEBS | E-tattoo | ~1250<br>(100 Hz) | ECG/EMG<br>≈ SNR | 120% strain;<br>1000 cycles (30%) | ≈ | 25.3 | Stable under sweat | - |
| | 19 | Au/Parylene | E-tattoo | 44<br>(1 kHz) | ECG/EMG<br>≈ SNR ≈ noise | 100 cycles (60%) | ↓ | - | 10 h | - |
| | 20 | Au/PU-PDMS | E-tattoo | 144<br>(100 Hz) | ECG<br>↓ SNR | 1100 bends | - | 25 | 6 days | - |
| **This Work (CNT)** | | **FWCNT** | **E-tattoo** | **13<br>(10 Hz)** | **ECG/EMG/EEG<br>↑ SNR ↓ noise** | **2,000 cycles (30%)<br>1,000 bends (3 mm)** | ↓ | **49** | **14 days** | **Better than wet;<br>Stable over 2 wks** |

**Notes:**

*Reported skin-electrode contact impedance (|Z|) are taken at the specified frequency and normalized by electrode area, assuming two electrodes.

*Signal quality vs. wet Ag/AgCl electrodes is reported based on metrics provided in the referenced works, including signal-to-noise ratio (SNR), RMS noise, or qualitative visual comparison of waveform fidelity.

*Compared to wet Ag/AgCl electrodes, ↑, ≈, and ↓ indicate lower, comparable, or higher values respectively, and - indicates not reported.

*Breathability is reported either quantitatively via water vapor transmission rate (WVTR), when available, or qualitatively based on the electrodes' intrinsic form factor and physical structure or physiological metrics as described in the referenced works. - indicates not reported.

**Supplementary Table S4 |** Performance comparison of other emerging electrophysiology electrodes.

| Material class | ref # | Material System | Form Factor | \|Z\| kΩ·cm² | Signal (vs. wet) | Mechanical Stability | Motion Noise | Breathability ($g \cdot m^{-2} \cdot h^{-1}$) | Extended Wear | ML Stability |
|---|---|---|---|---|---|---|---|---|---|---|
| Liquid Metal | 21 | EGaIn/Ag-Cu/PU/PMA | Semi-liquid metal fibers | 90 (60 Hz) | ECG ≈ visual | 180% strain 10,000 cycles (100%) | ↓ | ~100 | 24 h | - |
| | 22 | EGaIn/PU | E-tattoo | ~70 (100 Hz) | ECG/ICG ↓ SNR | 1000 cycles (500%) 100 peels | ↓ | 204 | Stable Under water | - |
| | 23 | LMNF/PDMS | E-tattoo | ~300 (10Hz) 9 (1 kHz) | EMG ↑ SNR | 60% strain 37000 cycles | ↓ | 21 | - | 97.5% accuracy; Stability - |
| Hydro-gel | 24 | PAAM/PSBMA/PAM-C-S | Headband | - | EEG/EMG ↑ SNR | 700% strain 20 peels | ≈ | - | Stable under sweat | 90% accuracy; Stability - |
| | 25 | Gelatin/Borax/Glycerol/PU | Self-adhesive patch | 49 (100 Hz) | ECG/EEG/EOG ≈ SNR | 696% strain 1000 cycles (100%) | ↓ | 70 | 8 days | - |
| | 26 | PAAC/PSBMA/PVA | Patch | 16 (1 kHz) | ECG/EMG ↑ SNR | 20 peels | ≈ | - | Stable under sweat | - |
| Micro-machined | 27 | Ag/AgCl/TPU | Microstructured Patch | 31 (10 Hz) | ECG ≈ SNR | 5000 bends (8mm) | ↓ | - | - | - |
| | 28 | AgLMP/PDMS | Microstructured Patch | 50 (10 Hz) | ECG/EEG ≈ SNR | 10 peels | ≈ | - | Stable under water | - |
| | 29 | Au/Ni/Cu/Methacrylate | Micromachined Earpiece | 72 (50 Hz) | EEG ≈ accuracy | - | ↑ | - | 40 h | 93% accuracy; Stability - |
| **This Work (CNT)** | | **FWCNT** | **E-tattoo** | **13 (10 Hz)** | **ECG/EMG/EEG ↑ SNR ↓ noise** | **2,000 cycles (30%) 1,000 bends (3 mm)** | ↓ | **49** | **14 days** | **Better than wet; Stable over 2 wks** |

**Notes:**

*Reported skin-electrode contact impedance (|Z|) are taken at the specified frequency and normalized by electrode area, assuming two electrodes.

*Signal quality vs. wet Ag/AgCl electrodes is reported based on metrics provided in the referenced works, including signal-to-noise ratio (SNR), RMS noise, or qualitative visual comparison of waveform fidelity.

*Compared to wet Ag/AgCl electrodes, ↑, ≈, and ↓ indicate lower, comparable, or higher values respectively, and - indicates not reported.

*Breathability is reported either quantitatively via water vapor transmission rate (WVTR), when available, or qualitatively based on the electrodes' intrinsic form factor and physical structure or physiological metrics as described in the referenced works. - indicates not reported.

**Supplementary Note S1 |** Determination of synthetic concentrations.

Literature values were used to determine the concentration of ferrocene and sulfur in the reactor[30,31]

Gas flow rates are denoted by $Q$, where the total flow into the reactor is $Q_{\mathrm{Total}}$. The flow streams passing through the ferrocene and sulfur reagent cartridges are denoted $Q_{\mathrm{FeCp_2}}$ and $Q_{\mathrm{Sulfur}}$, respectively. The carrier gas stream, which carries the bulk of the inert gas and the hydrogen, is denoted as $Q_{\mathrm{Carrier}}$. Upon exiting the individual cartridges and merging into the main gas line, the total flow is given by

$$Q_{\mathrm{Sulfur}} + Q_{\mathrm{FeCp_2}} + Q_{\mathrm{Carrier}} = Q_{\mathrm{Total}}$$

For these calculations, the gas streams flowing over the sulfur and ferrocene cartridges are assumed to be fully saturated with the corresponding vapor species. Under this assumption, the molar concentration of each vaporized species is determined by its saturated vapor pressure and the ideal gas law, such that

$$c_x = \frac{P_{\mathrm{vap,x}}}{RT}$$

where $P_{\mathrm{vap},x}$ is the saturated vapor pressure of species $x$, $R$ is the gas constant, and $T$ is the absolute temperature. The saturated vapor pressures of ferrocene and sulfur are modeled as

$$\boldsymbol{c}_{\mathbf{vap,FeCp_2}} = {P_{\mathrm{vap,FeCp_2}}}/{RT} = \frac{P_0}{RT} e^{-\frac{E_{\mathrm{FeCp_2}}}{RT}}$$

$$\boldsymbol{c}_{\mathbf{vap,Sulfur}} = {P_{\mathrm{vap,Sulfur}}}/{RT} = \frac{P_0}{RT} e^{-\frac{E_{\mathrm{sulfur}}}{RT}}$$

where $P_0$ is a parameterized constant, $R$ is the gas constant, and $E_{sulfur}$ and $E_{FeCp_2}$ are the heats of vaporization of the two reagents. The effective concentration of each reagent in the combined reactor flow is then weighted by the fractional flow rate through its corresponding cartridge:

$$[x] = \frac{c_{\mathrm{vap,x}}\, Q_x}{Q_{\mathrm{Total}}} \left[\frac{\mathrm{mol}}{\mathrm{cm}^3}\right]$$

Once fitted, these values for concentration can then be used to calculate the concentration of the main reagents in the reaction zone. Substituting these expressions into the flow-weighted concentration equations yields the effective ferrocene and sulfur concentrations in the reaction zone,

$$[\mathbf{FeCp_2}] = \frac{\boldsymbol{c}_{\mathbf{vap,FeCp_2}} \cdot Q_{\mathrm{FeCp_2}}}{Q_{\mathrm{Total}}}$$

$$[\mathbf{Sulfur}] = \frac{\boldsymbol{c}_{\mathbf{vap,Sulfur}} \cdot Q_{\mathrm{Sulfur}}}{Q_{\mathrm{Total}}}$$

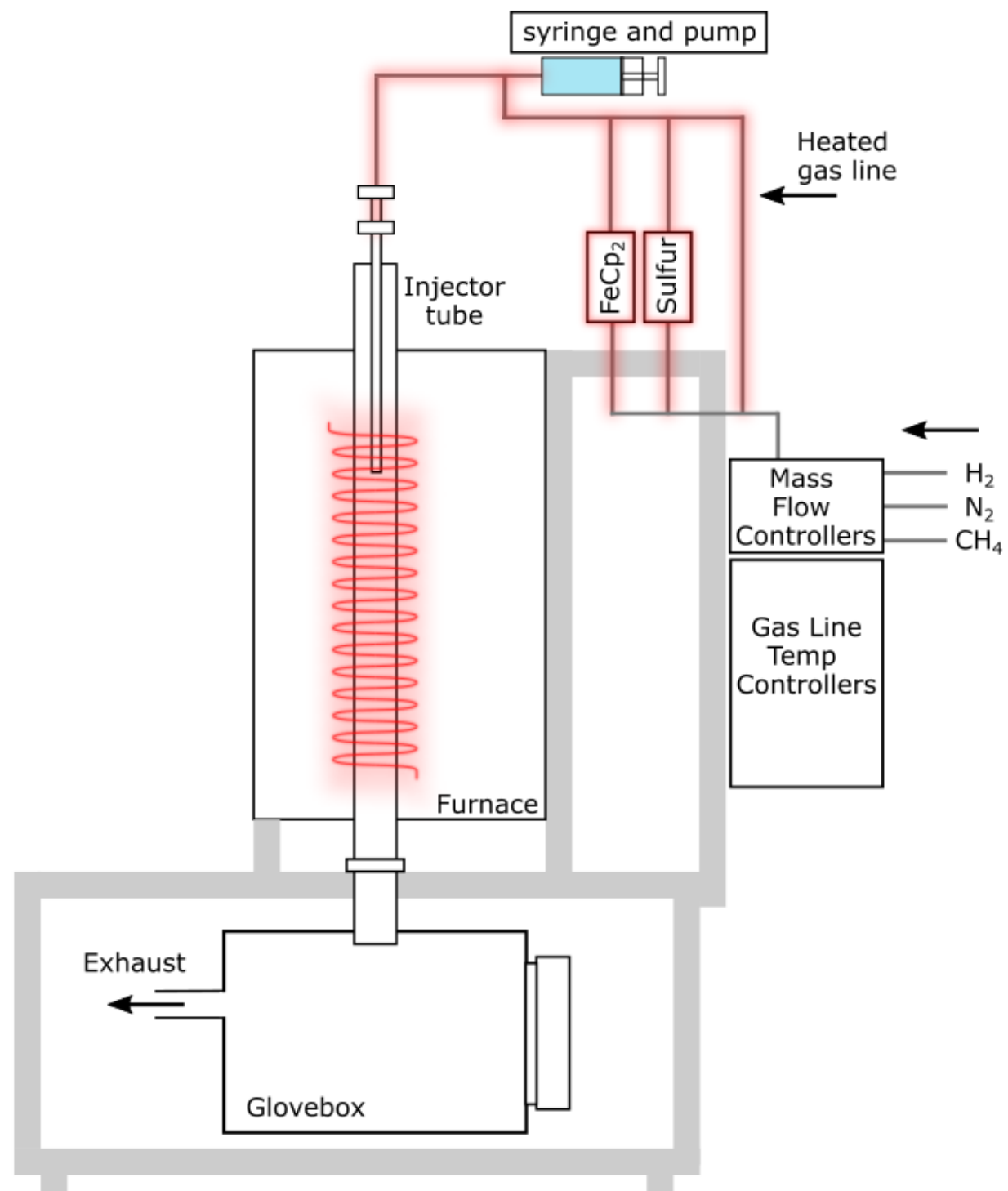


**Supplementary Figure S1** | Vertical reactor configuration.

The diagram illustrates the placement of the sulfur and ferrocene cartridges and the carbon source within the vertical reactor configuration. The individual flow contributions sum to the total flow entering the main furnace.

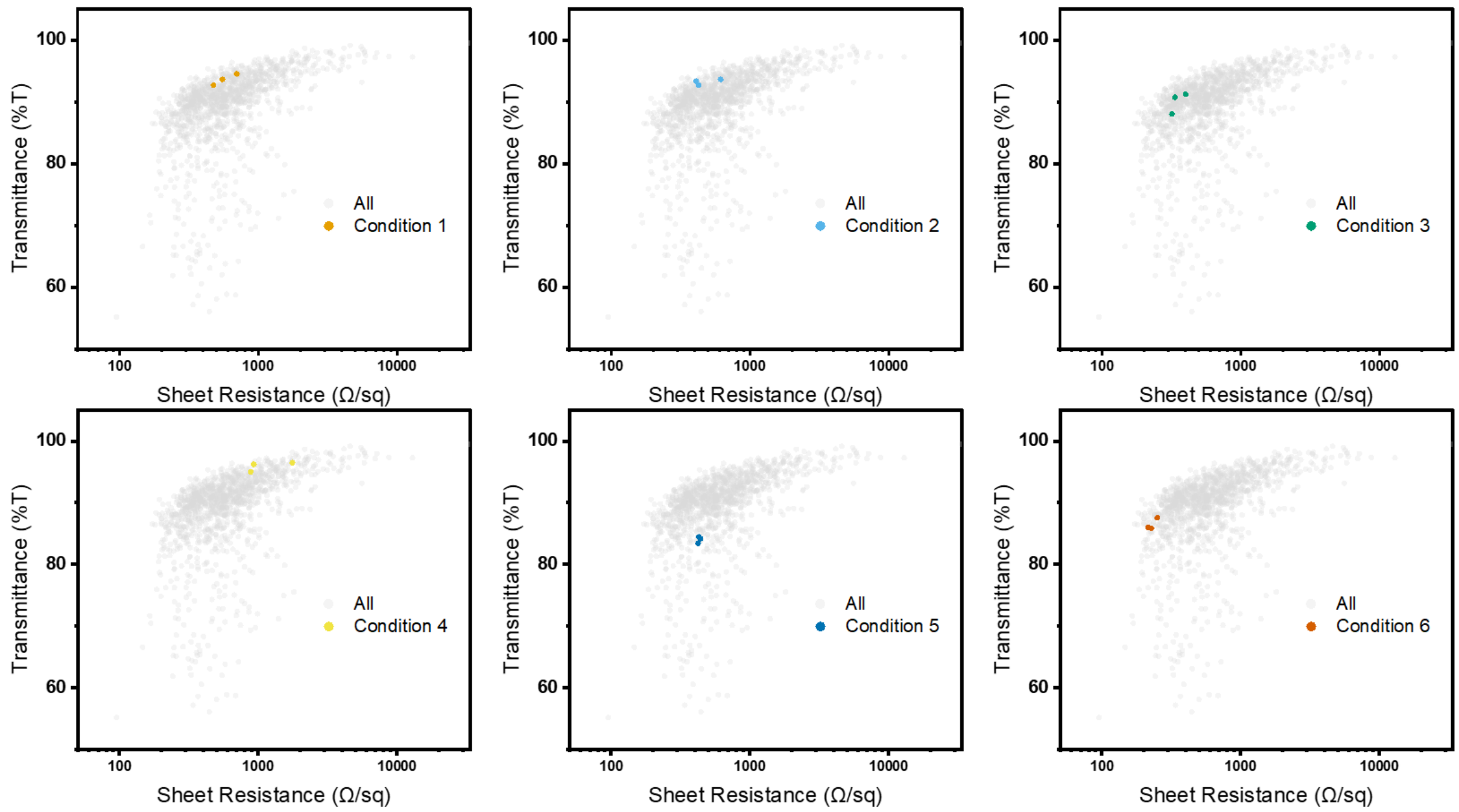


**Supplementary Figure S2** | Stability of CNT synthesis conditions.

The stability of each synthesis conditions is evaluated by characterizing sheet resistance and optical transmittance and observing the drift over time. For a given condition, measurements were taken at approximately 5-min intervals to assess the relative drift. When switching between synthesis conditions, a minimum settling time of 20 min was allowed before sampling to ensure that the reactor has reached the new steady-state condition.

**Supplementary Table S5** | Representative synthesis conditions.

Six randomly selected experimental conditions are listed to demonstrate performance variations across specific synthesis parameters. From left to right, the table report the carbon, iron, and sulfur sources, and the flow rate of the carbon-alcohol precursor, followed by the three-zone furnace temperatures and the corresponding temperatures of the reagent cartridges. The final columns summarize the individual gas-line flow rates that combine to form the total reactor inlet stream. Each condition was repeated three times to capture the performance variability.

| | Reagents | | | | Gas Flow | | | | |
|---|---|---|---|---|---|---|---|---|---|
| Condition Number | Carbon source | Iron source | Sulfur source | C Feeding (mL/min) | Main Line (sccm) | FeCp2 Line (sccm) | H2 Line (sccm) | Sulfur Line (sccm) | Total Flow (sccm) |
| 1 | EtOH | Ferrocene | powder | 3.6 | 1250 | 220 | 400 | 350 | 2220 |
| 2 | EtOH | Ferrocene | powder | 4.2 | 1230 | 220 | 500 | 350 | 2300 |
| 3 | EtOH | Ferrocene | powder | 3.3 | 1320 | 130 | 545 | 330 | 2325 |
| 4 | BuOH | Ferrocene | powder | 2.2 | 1150 | 120 | 450 | 250 | 1970 |
| 5 | BuOH | Ferrocene | powder | 2.3 | 720 | 150 | 378 | 264 | 1512 |
| 6 | EtOH | Ferrocene | powder | 4.2 | 1130 | 150 | 595 | 400 | 2275 |

**Supplementary Table S6** | Performance and stability for representative synthesis conditions.

The table summarizes the performance metrics measured for several representative experimental conditions, selected to show the degree of variability observed within each condition. CNT films were collected during three separate runs per condition to show the reactor stability.

| | Performance (Sheet resistance & Transmittance) | | | | | |
|---|---|---|---|---|---|---|
| Condition Number | Rs (ohm/sq) | | | T (%) | Avg. Rs (ohm/sq) | sd |
| | 1 | 2 | 3 | | | |
| 1 | 591 | 563 | 499 | **93.6** | **551** | **38.5** |
| | 714 | 746 | 636 | **94.5** | **699** | **46.2** |
| | 477 | 477 | 470 | **92.7** | **475** | **3.30** |
| 2 | 436 | 405 | 437 | **92.7** | **426** | **14.9** |
| | 403 | 417 | 410 | **93.3** | **410** | **5.71** |
| | 636 | 619 | 586 | **93.6** | **614** | **20.8** |
| 3 | 422 | 366 | 412 | **91.2** | **400** | **24.4** |
| | 320 | 319 | 315 | **88.0** | **318** | **2.16** |
| | 338 | 353 | 316 | **90.8** | **336** | **15.2** |
| 4 | 881 | 869 | 890 | **95.0** | **880** | **8.60** |
| | 1790 | 1737 | 1755 | **96.5** | **1760** | **22.0** |
| | 909 | 962 | 908 | **96.2** | **926** | **25.2** |
| 5 | 418 | 403 | 447 | **83.4** | **423** | **18.3** |
| | 441 | 435 | 445 | **84.2** | **440** | **4.11** |
| | 424 | 414 | 444 | **84.5** | **427** | **12.5** |
| 6 | 229 | 227 | 223 | **85.9** | **226** | **2.49** |
| | 211 | 208 | 227 | **86.0** | **215** | **8.34** |
| | 251 | 248 | 253 | **87.6** | **251** | **2.06** |

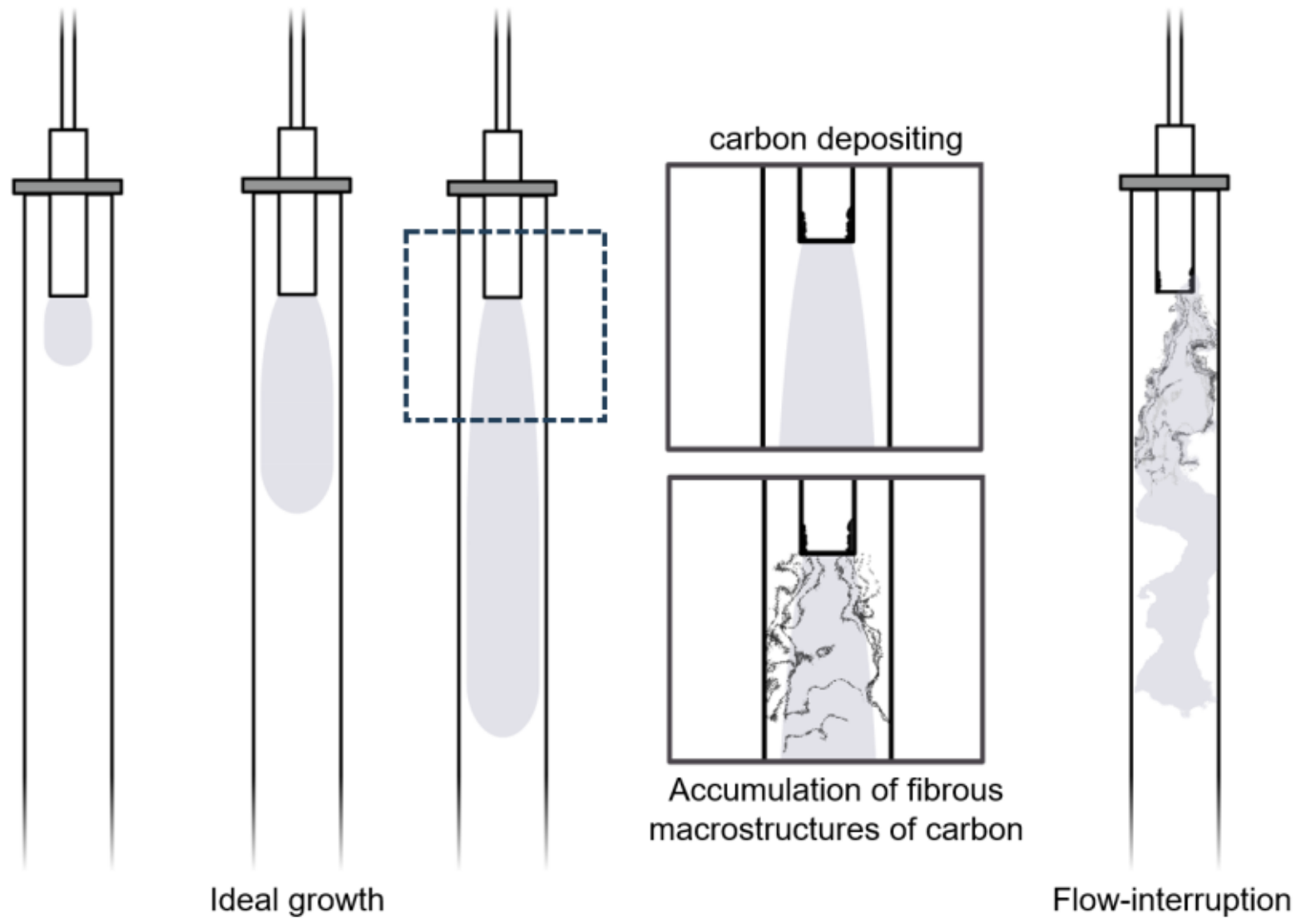


**Supplementary Figure S3 |** Ideal versus interrupted formation of CNT aerogel bubbles.

Schematic illustration of the “clogging” failure mode during CNT aerogel synthesis, which prevents the formation of testable films. Under such growth conditions, excessive carbon deposition occurs at the tip of the internal quartz injector, leading to the formation of fibrous carbon macrostructures. The continuously formed CNT aerogel film becomes entangled within these macrostructures, disrupting the local flow dynamics in the furnace and preventing stable aerogel bubble formation and continuous draw-out from the reactor. As a result, these conditions do not yield mechanically stable or homogeneous CNT films. If the reactor is operated far outside the normal synthesis window, clogging will occur rapidly especially at higher carbon concentration in the gas stream. Within normal reaction conditions, however, the reactor can be operated for several hours before clogging becomes an issue.

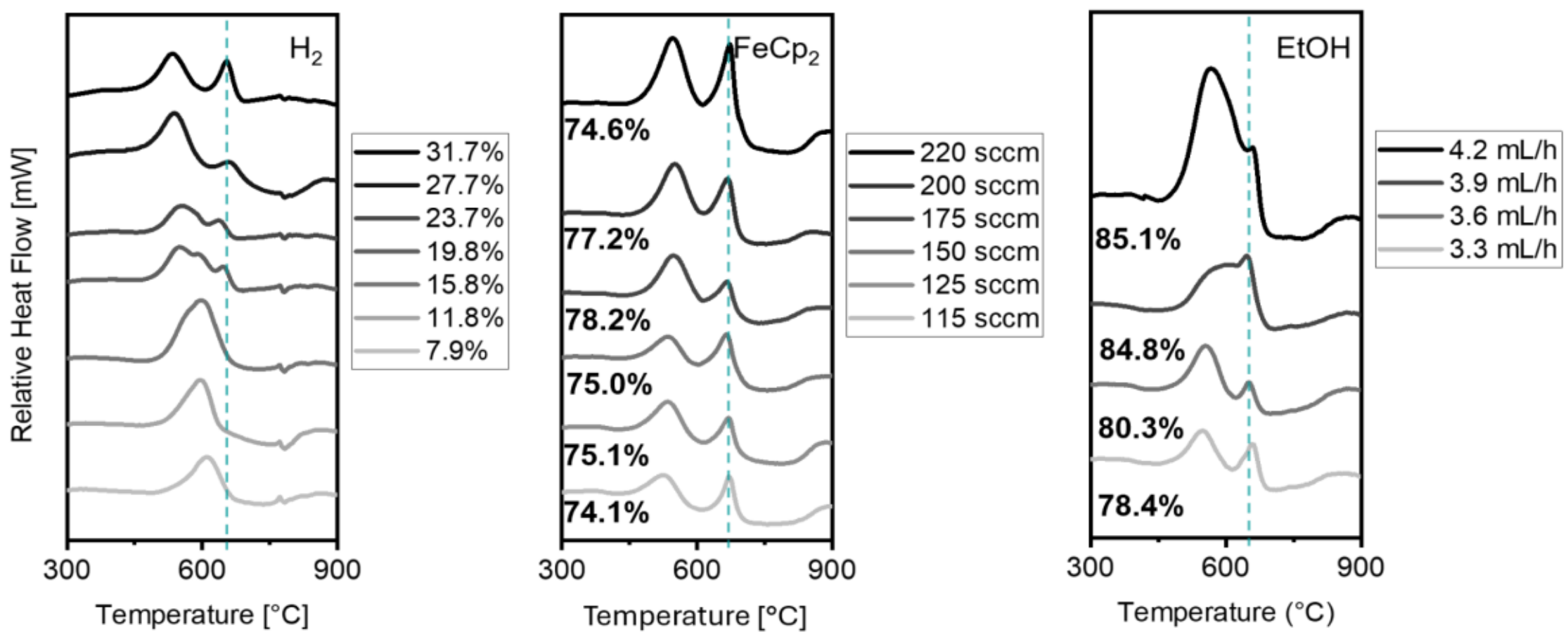


**Supplementary Figure S4 |** Thermal analysis of CNT precursor decomposition under varying synthesis parameters.

Differential heat flow traces of (left) hydrogen, (middle) ferrocene ($FeCp_2$), and (right) ethanol during CNT film synthesis. The vertical dashed line at ~600 °C marks the onset of catalyst activation and CNT growth. Increasing $H_2$ concentration broadens and shifts the decomposition peak, reflecting its role in controlling carbon supersaturation and defect density. $FeCp_2$ decomposition remains stable across flow rates (115–220 sccm), confirming consistent catalyst formation. Increasing ethanol feed rate enhances carbon supply and CNT yield up to saturation.

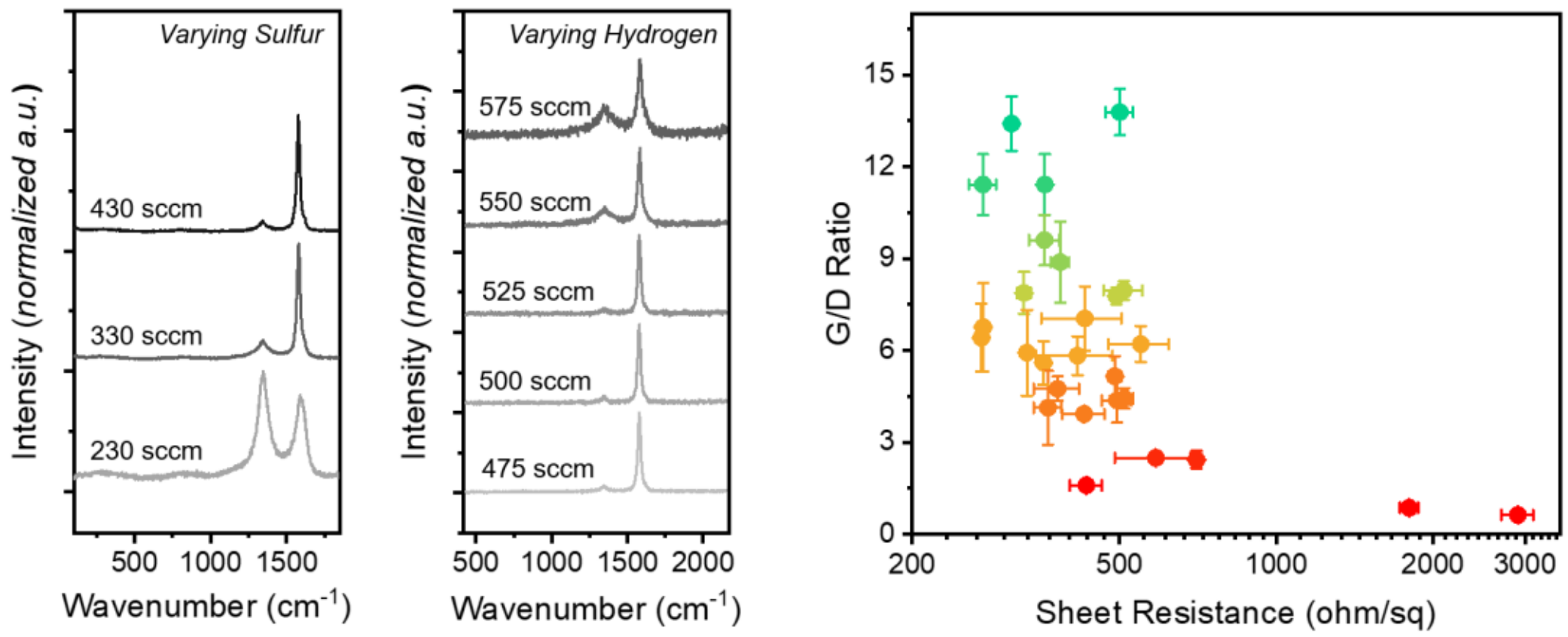


**Supplementary Figure S5 |** Relationships between varied sulfur and hydrogen flows, with corresponding representative Raman spectra. Relationship between CNT film quality and conductivity, showing that higher Raman G/D ratios (>9) generally correspond with lower sheet resistances, consistent with low defect density and large-diameter nanotubes synthesized under Argon flow.

The Raman G/D intensity ratio is widely used as an indicator of structural quality in CNTs, with higher ratios corresponding to reduced defect density, longer tube length, and improved crystallinity. The CNT films synthesized in this work exhibited high G/D ratios, exceeding 12 when considering the peak area ratios ($A_{\mathrm{G}}/A_{\mathrm{D}}$), and exceeding 30 when considering the peak height ratios ($I_{\mathrm{G}}/I_{\mathrm{D}}$). These values are indicative of highly graphitic networks with minimal amorphous carbon or tube breakage. Such high values are consistent with low junction resistance and enhanced carrier mobility observed in the electrical measurements. It is important to note, however, that the absolute magnitude of the G/D ratio also depends on the nanotube diameter distribution. Larger-diameter or multi-walled CNTs tend to display intrinsically weaker D-band intensity due to reduced curvature-induced disorder, even at comparable defect densities. This effect partially contributes to the elevated G/D ratios observed here and aligns with previous reports of aerosol-grown CNTs with diameters in the range of 10–20 nm. Therefore, the high Raman quality factor observed is attributed to both the low defect density and the relatively large tube diameter characteristic of this synthesis regime.

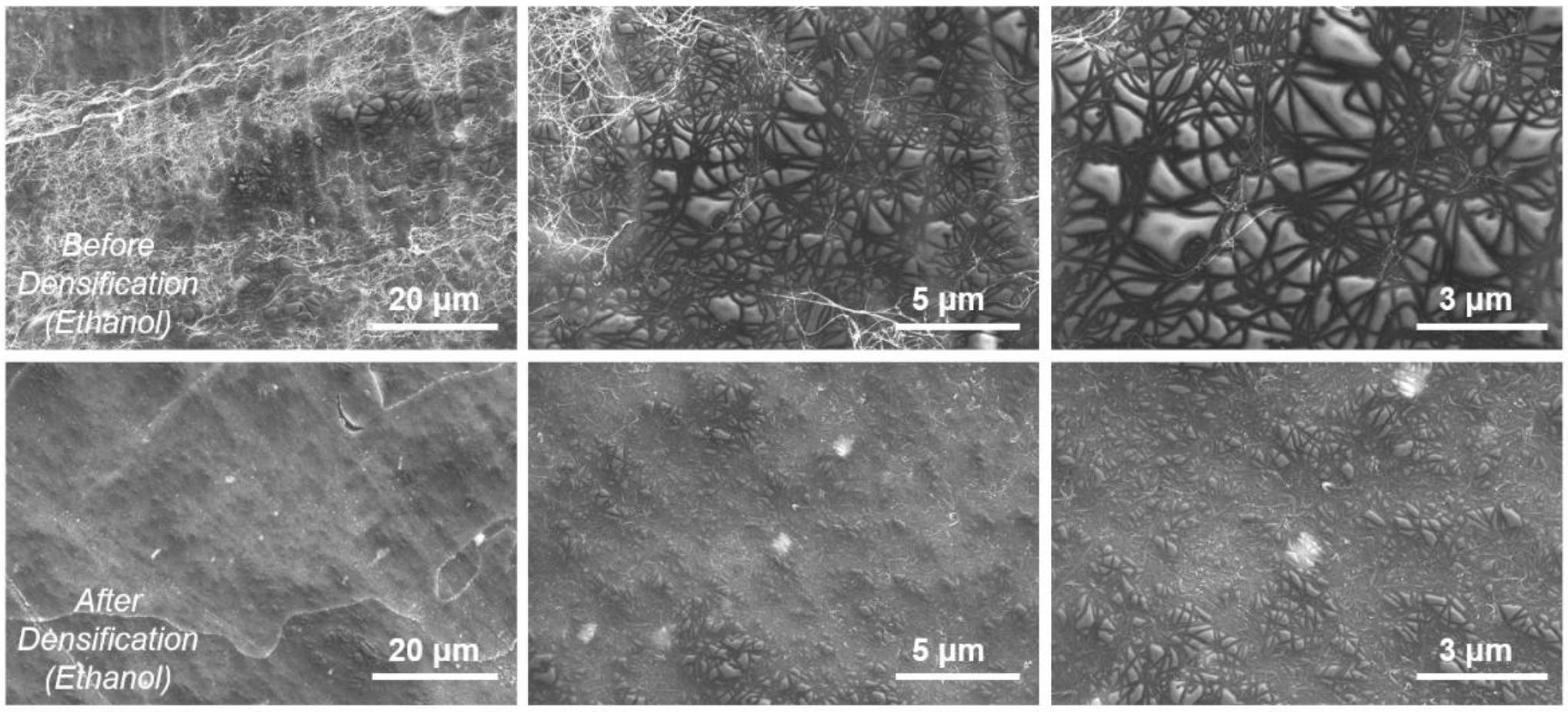


**Supplementary Figure S6 |** SEM images of entangled CNT network morphology of a single deposited layer.

The as-deposited CNT film already exhibits an entangled, interconnected network morphology before densification. After ethanol densification, the film becomes more compact while retaining the continuous CNT network structure. The preserved network morphology supports that each deposited CNT layer forms a mechanically continuous and electrically percolated CNT film rather than isolated CNT aggregates. Scale bars: 20 μm, 5 μm, and 3 μm.

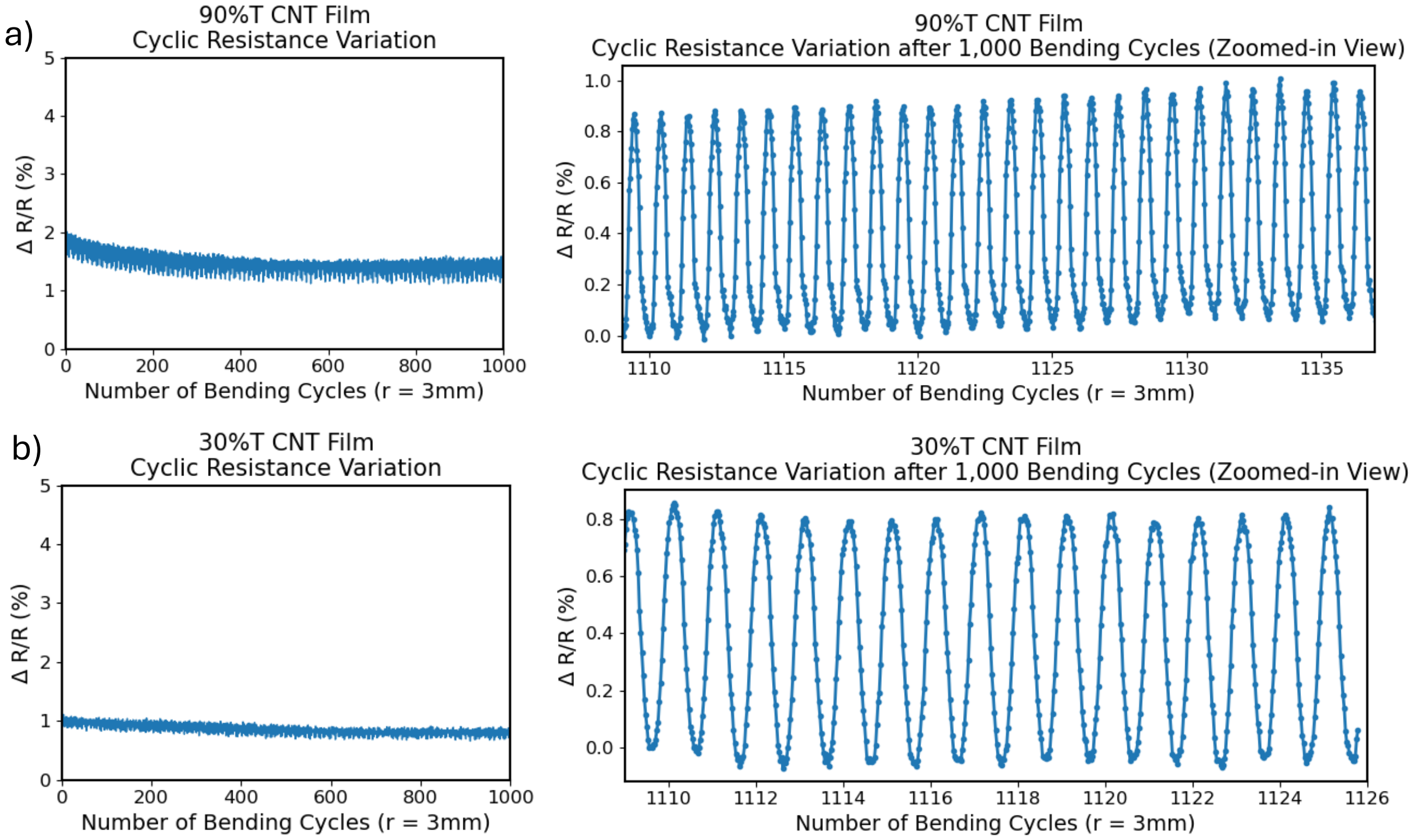


**Supplementary Figure S7 |** Cyclic resistance response for CNT films of 90%*T* (a) and 30%*T* (b), recorded over 1,000 bending cycles at a bending radius of 3 mm. The applied bending radius of 3 mm is smaller than typical radii experienced during natural human motion (generally ≥ 5–10 mm), representing a conservative mechanical fatigue condition for wearable applications.

Both films showed minimal resistance change throughout repeated deformation, with the 90%T film having less than 2% variation and the denser 30%*T* film remaining around 1% variation. The lower resistance variation of the 30%*T* film is consistent with its denser CNT network, while both films demonstrate robust electromechanical stability under cyclic deformation.

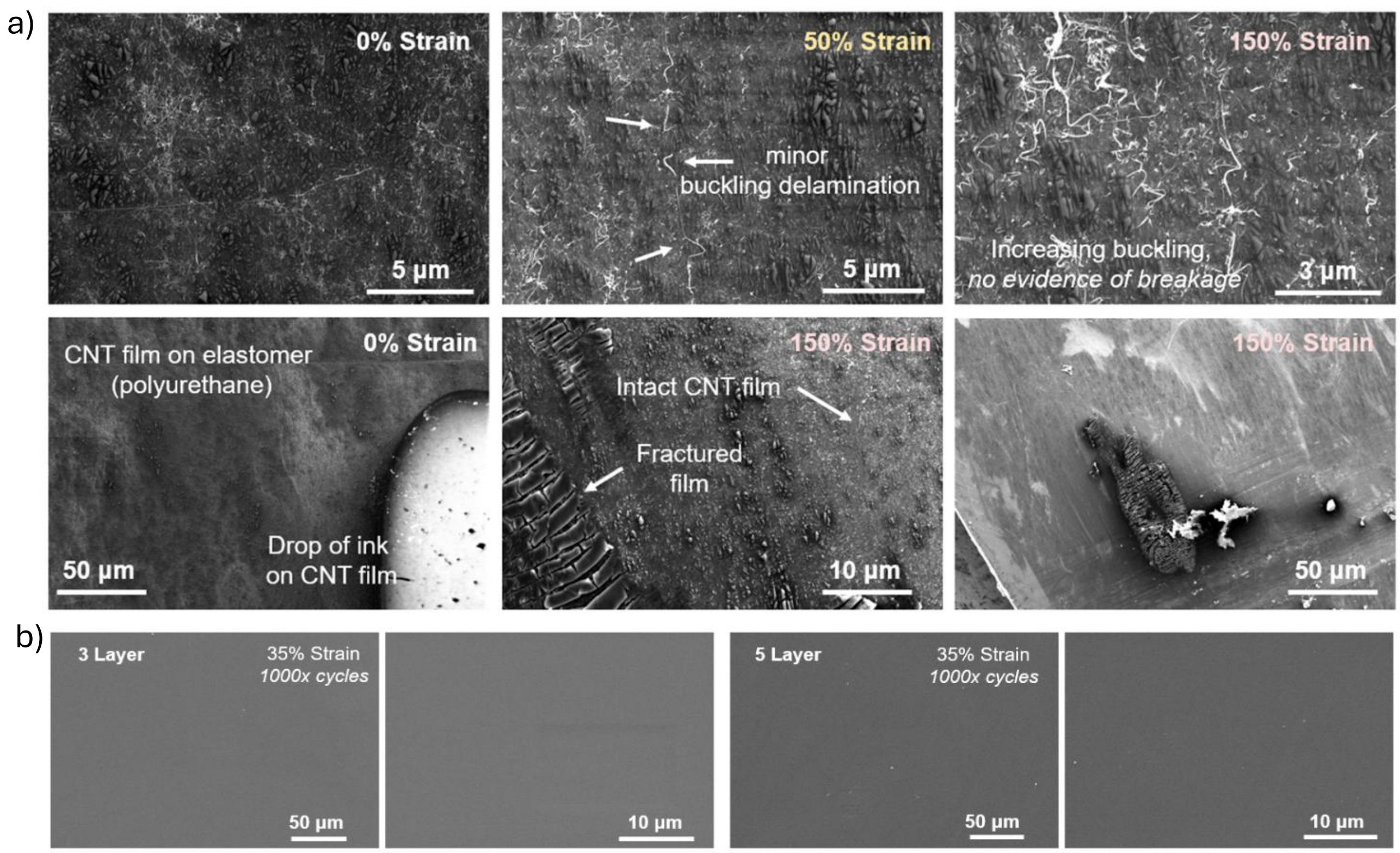


**Supplementary Figure S8 |** SEM images of CNT films on polyurethane elastomer.
a, SEM images of CNT after various tensile strains. At 0% strain, the CNT film forms a continuous, entangled network on the substrate. At 50% strain, minor buckling and local delamination are observed, while the film remains largely continuous. At 150% strain, buckling becomes more pronounced, but no obvious CNT network breakage is observed. Fractured film was observed when additional drop of ink was deposited on top of the CNT film, causing mechanical mismatch between the ink and the substrate.
b, Tacky-transfer test on 3-layer and 5-layer CNT films after 1000 stretch–release cycles at 35% strain. SEM images of the transfer surface showed no observable CNT fragments or CNT residues after mechanical cycling, supporting that the interconnected CNT network maintains structural integrity and does not readily shed detectable nanotube debris under the tested conditions.

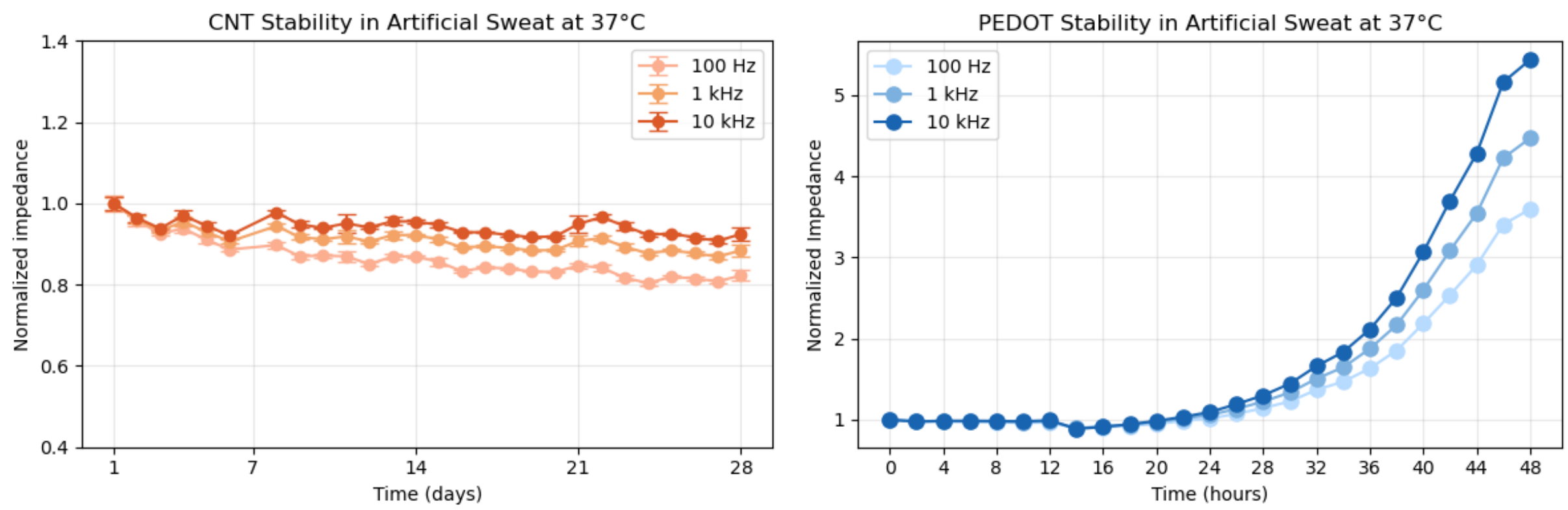


**Supplementary Figure S9 |** Electrochemical stability of CNT electrodes during continuous immersion in artificial sweat at 37°C compared with PEDOT electrodes.

CNT electrodes maintained stable impedance over 28 days. PEDOT electrodes showed a rapid increase in normalized impedance within 48 h, indicating degradation of electrochemical stability in artificial sweat at elevated temperature.

**Supplementary Table S7 |** Comparison of candidate collection substrates for CNT film collection and dry press-transfer process. Evaluation of substrate surface chemistry and CNT film adhesion/release behavior.

| Substrate | Surface Chemistry / Mechanism | CNT Film Behavior |
|---|---|---|
| Weighing paper | Polyethylene coating provides a smooth, semi-hydrophobic finish. | Semi-stick behavior; incomplete CNT release. |
| Parchment paper | Silicone-coated surface with low adhesion energy. | CNTs adhere strongly and cannot be retransferred. |
| Glassine paper | Super-calendared, smooth, dense cellulose surface with limited porosity. | Semi-stick behavior; partial CNT release possible. |
| Wax paper | Hydrophobic wax coating reduces surface energy. | CNTs adhere strongly; cannot be retransferred. |
| Freezer paper | Polyethylene-coated surface, similar to weighing paper. | CNTs adhere strongly; cannot be retransferred. |
| Teflon | Extremely low surface energy and non-wetting surface. | Very poor CNT attachment; film continuity not maintained. |
| **Tracing paper *(Optimal)*** | **Pure cellulose fibers produced via sulfite pulping; low to moderate surface energy.** | **CNTs attach uniformly and can be cleanly retransferred.** |

Moderate-surface-energy cellulose substrates such as tracing paper enable reversible CNT attachment, while hydrophobic or ultra-low-energy surfaces prevent uniform collection or retransfer.

**Supplementary Method S1 |** Layer-by-layer stacking method and optical ttransmittance as a proxy for CNT thin film thickness.

In the experimental framework in this work, films of varying thicknesses were not synthesized directly. Instead, thickness variation was achieved by sequentially stacking identical layers. CNT thin films were synthesized under identical conditions yielding a single-layer transmittance of 90% at 550 nm. Thicker films were subsequently obtained by sequentially stacking these single-layer films to form multilayer constructs. The primary motivation for this layer-by-layer approach is to ensure that intrinsic material properties remain constant throughout the study, isolating macroscopic thickness as the sole variable. Direct synthesis of thicker films often alters fundamental material properties, such as film morphology, bundle density, and junction resistance, due to variations in synthesis conditions and dynamics.

For inherently inhomogeneous thin film materials, such as random nanotube networks, it is assumed that the effective or average film thickness scales linearly with the number of stacked layers $N$. This assumption permits the treatment of the film as a composite of independent, statistically identical layers.

Although the physical number of layers $N$ serves as the controlled experimental variable, optical transmittance $T$ is utilized as the primary independent variable for modeling and analysis for the following reasons. First, transmittance provides a direct measure of the "optical thickness" of the material, which exhibits a robust correlation with the mass density of the network. Moreover, as a standard metric for characterizing transparent conducting films (TCFs), the use of transmittance allows results to be easily compared with existing literature and industry standards. Lastly, unlike the discrete integer value of layers $N$, transmittance $T$ offers a continuous variable capable of capturing subtle density variations that may occur even within a single layer.

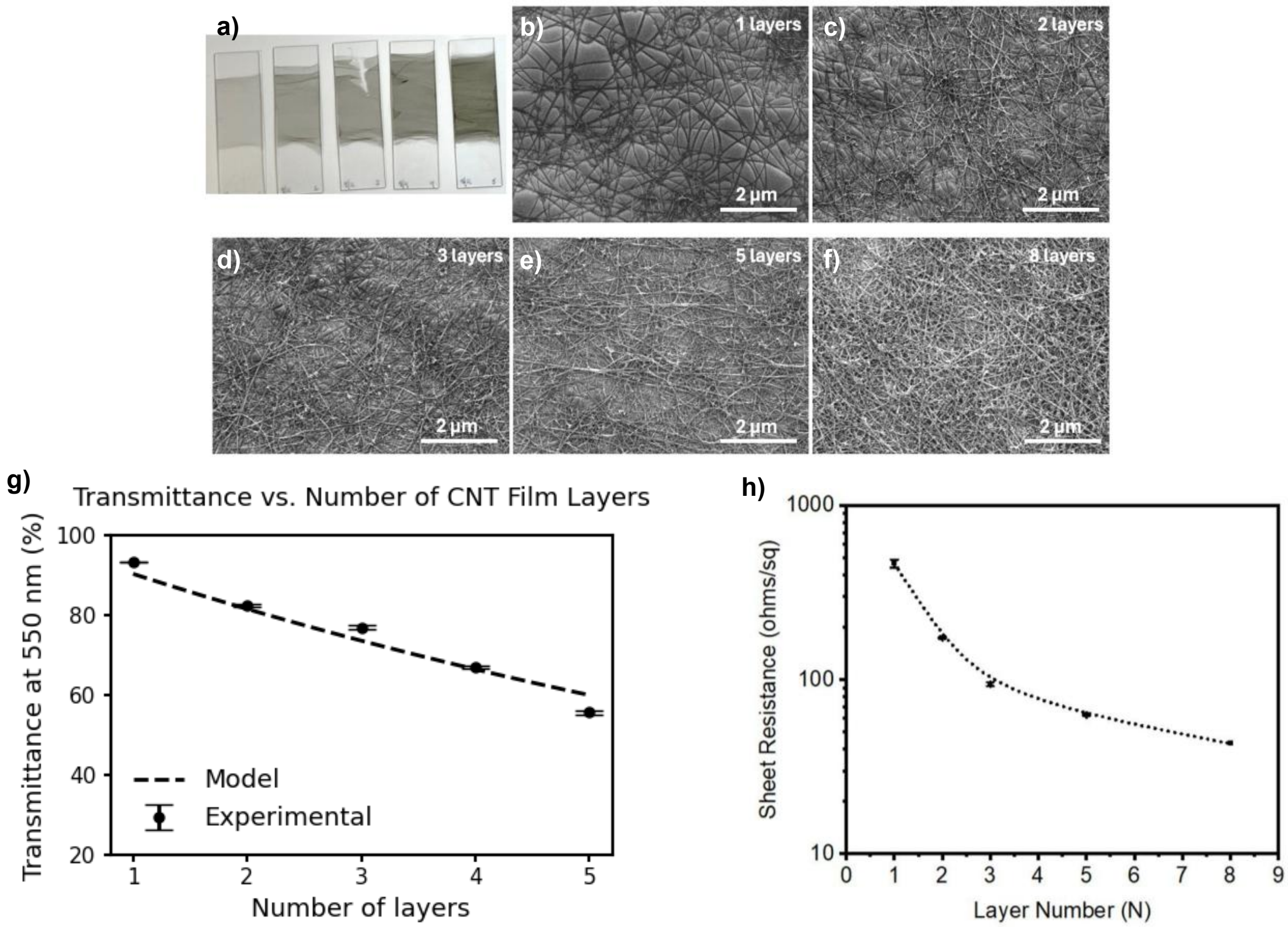


**Supplementary Figure S10 |** Optical characterization of stacked CNT thin films.
a, Photographs of CNT films with increasing numbers of stacked layers (1–5). b–f, SEM images showing the evolution of network density with layer number, where 1 layer corresponds to a film synthesized at 93% transmittance at 550 nm. Increasing the number of stacked layers systematically reduces film porosity and increases percolation density. g, Optical transmittance at 550 nm as a function of layer number, showing consistency between experimental and theoretical values. h, Sheet resistance as a function of layer number, showing consistency between experimental and theoretical values. The results confirm that stacking identical CNT layers yields a predictable exponential reduction in sheet resistance and optical transmittance with film thickness.

The optical transmittance of each stack was characterized by UV–Vis spectroscopy and compared with theoretical predictions. The excellent agreement between measured and calculated values confirms that stacking of the same CNT film material produces an exponential attenuation in transmittance with increasing layer number (or effective thickness). Corresponding SEM micrographs illustrate the progressive densification of the CNT network and reduction in film porosity with additional layers.

**Supplementary Note S2 |** Modeling framework for skin–electrode contact impedance in CNT thin-film dry electrodes.

An analytical framework for the skin–electrode contact impedance of CNT thin-film dry electrodes is derived by decomposing the system into three physically distinct parameters: areal coverage, sheet resistance, and conformability. Each parameter captures a different aspect of the electrode–skin interface and can be independently characterized.

Areal coverage $\boldsymbol{\phi}$ describes the fraction of the substrate covered by the conductive network. This parameter is critical for understanding the nanoscopic interfacial area between the electrode and the skin. Sheet resistance $R_s$ characterizes the intrinsic electrical resistance of the percolating CNT network and captures charge transport within the film. Conformability $\varphi$ describes the film's ability to maintain coverage over topological features, thereby determining the effective microscopic contact area at the electrode–skin interface. By analyzing these parameters independently, a combined model can be constructed to describe the unique interfacial contact behavior of ultrathin, inhomogeneous nanotube films.

Previous studies have shown that key dry-electrode properties, including conformability, scale directly with film thickness[32]. For inherently inhomogeneous thin-film materials such as CNT networks, optical transmittance $T$ is a more practical proxy for effective film thickness ("optical thickness").

A commonly used two-domain optical model expresses the transmittance as

$$T = (1 - \boldsymbol{\phi}) + \boldsymbol{\phi}\, e^{-\alpha t}$$

where $\boldsymbol{\phi}$ is the fraction of the surface covered by CNTs, $t$ is the local film thickness, and $\alpha$ is the absorption coefficient. The first term represents uncovered regions of the substrate, while the second term accounts for absorption in CNT-covered regions.

However, in sparse or intermediate coverage regimes typical of transparent thin films, the local thickness varies substantially across the film and increases in deposited material primarily serve to fill previously uncovered regions rather than uniformly increasing thickness. As a result, this model is only valid in the linear regime and is more appropriately applied to optically thick films, where Beer–Lambert attenuation dominates. To accurately resolve the coupled relationship between areal coverage, thickness distribution, and optical transmittance in ultrathin CNT films, a Boolean model[33–35] is adopted instead to describe percolated the CNT network in the thin-film regime, as detailed in Supplementary Note S3.

**Supplementary Note S3 |** Boolean model for random tube deposition: areal coverage, thickness statistics, and optical transmittance.

1. Boolean Model Problem Statement and Definitions

Consider a square domain (sheet) of dimensions $L \times L$ with total area $S_{total} = L^2$. A total of $n_0$ tubes, each of length $L$ and width $W$ are deposited sequentially onto the domain. The position of each tube is uniformly distributed across the domain width. It is assumed that the tube width is small relative to the domain size $\mathrm{W} \ll \mathrm{L}$.

Let $p$ be the probability that a specific point on the sheet is covered by a single randomly placed tube. This is given by the ratio of the tube area to the sheet area:

$$p = \frac{L \cdot W}{L^2} = \frac{W}{L}$$

One layer of thin film is defined as the deposition of $n_0$ tubes. A system composed of $N$ such layers is considered.

2. Areal Coverage $\phi$

**Proposition 1**

The fractional areal coverage $\phi$ of a surface after the deposition of $N$ layers, each consisting of $n_0$ tubes, is given by:

$$\phi \approx 1 - e^{-N\lambda}$$

where $\lambda = n_0 \frac{W}{L}$ is the nominal tube density per layer. Physically, $\lambda$ represents the expected number of tubes intersecting a given point per layer. Equivalently, letting $d$ denote the average tube diameter, the $\lambda d$ product corresponds to the average film thickness of a single layer, as derived in the corollary to Proposition 2.

*Proof*

Let $U_k$ denote the probability that an arbitrary point on the sheet remains uncovered (local thickness $t = 0$) after the deposition of $k$ tubes. This is derived via induction.

<u>Initial Case:</u>

Before any deposition ($k = 0$), the entire sheet is uncovered:

$$U_0 = 1$$

<u>Inductive Step:</u>

Consider the state after $k$ tubes have been placed. When the $(k+1)$-th tube is deposited, it covers a fraction $p$ of the domain. Since the placement is random, the probability that a specific point remains uncovered is the probability that it was previously uncovered and is not hit by the new tube.

$$U_{k+1} = U_k(1 - p)$$

Solving this recurrence relation yields:

$$U_{n_0} = U_0(1-p)^{n_0} = \left(1 - \frac{W}{L}\right)^{n_0}$$

Asymptotic Limit:
In the limit where $n_0 \gg 1$ and $p \ll 1$ such that the product $\lambda = n_0 p$ remains finite, the exponential limit definition $\lim_{n\to\infty}(1 - x/n)^n = e^{-x}$ is applied.
Let $A_0$ be the uncovered fraction for a single layer ($n_0$ tubes; $N = 1$).

$$A_0 = \lim_{n_0\to\infty}\left(1 - \frac{\lambda}{n_0}\right)^{n_0} = e^{-\lambda}$$

Extension to $N$ Layers:
For a stack of $N$ independent layers, the probability that a point remains uncovered is the product of the probabilities for each layer:

$$U_{total} = (A_0)^N = \left(e^{-\lambda}\right)^N = e^{-N\lambda}$$

The areal coverage ϕ, defined as the fraction of the domain covered by at least one tube, is the complement of the uncovered fraction:

$$\phi = 1 - U_{total} = 1 - (A_0)^N = 1 - e^{-N\lambda}$$

*Q.E.D.*

3. Thickness Distribution

**Proposition 2**
The distribution of the local film thickness $t$ (number of overlapping tubes at a point) follows a Poisson distribution defined by the parameter λ:

$$P(t) = \frac{\lambda^t e^{-\lambda}}{t!}$$

*Proof*
Let $P_k(t)$ be the probability that a point has thickness $t$ after the deposition of $k$ tubes. This is derived via induction.

Initial Case:
For $k = 0$, the thickness is uniformly zero.

$$P_0(t) = \begin{cases} 1, & t = 0 \\ 0, & t > 0 \end{cases}$$

Inductive Step:
Upon depositing the $(k+1)$-th tube, the thickness at a point is $t$ either by increasing from $t-1$ to $t$ when hit (probability $p$), or by remaining $t$ when missed (probability $1-p$). This yields the recurrence relation:

$$P_{k+1}(t) = P_k(t)(1-p) + P_k(t-1)p$$

Solving this recurrence for $n_0$ tubes corresponds to the probability mass function of the Binomial distribution $B(n_0, p)$:

$$P_{n_0}(t) = \binom{n_0}{t} p^t (1-p)^{n_0 - t}$$

<u>Poisson Limit:</u>
Under the conditions $n_0 \to \infty$ and $p \to 0$ with $\lambda = n_0 p$ fixed, the Binomial distribution converges to the Poisson distribution:

$$\lim_{n_0 \to \infty} P_{n_0}(t) = \frac{\lambda^t e^{-\lambda}}{t!}$$

*Q.E.D.*

**Corollary: Average Thickness**
The expected value of the film thickness $\langle t \rangle$ is given by the first moment of the Poisson distribution. By definition, the mean of a Poisson distribution is:

$$\langle t \rangle = \lambda = n_0 \frac{W}{L}$$

This confirms that $\lambda$ physically represents the average thickness of the percolated network and scales linearly with the number of deposited tubes.

4. Optical Transmittance

**Proposition 3**
Let $\alpha$ be the absorbance coefficient per single tube. Following the Beer-Lambert law, the transmittance $T_0$ of one layer (consisting of $n_0$ tubes) is given by:

$$T_0 = e^{-\lambda(1-e^{-\alpha})}$$

*Proof*
The local transmittance $T(t)$ at a point with thickness $t$ (where $t$ is the number of stacked tubes) is defined by the Beer-Lambert law. For empty regions ($t = 0$), transmittance is 100% ($T = 1$). For regions with $t$ tubes, the transmittance decreases exponentially with absorbance $\alpha t$.

$$T(t) = \begin{cases} 1, & t = 0 \\ e^{-\alpha t}, & t > 0 \end{cases}$$

The total transmittance of the sheet is the expected value of the local transmittance over the entire area. This is calculated by summing the transmittance for each thickness weighted by the probability of that thickness occurring ($P(t)$ from Proposition 2).

$$T_0 = \sum_{t=0}^{\infty} P(t) \cdot T(t)$$

Substituting the Poisson distribution $P(t) = \frac{\lambda^t e^{-\lambda}}{t!}$ and $T(t) = e^{-\alpha t}$:

$$T_0 = \sum_{t=0}^{\infty} \left(\frac{\lambda^t e^{-\lambda}}{t!}\right) e^{-\alpha t}$$

Factoring out the constant term $e^{-\lambda}$ and combining the powers of $t$:

$$T_0 = e^{-\lambda} \sum_{t=0}^{\infty} \frac{(\lambda e^{-\alpha})^t}{t!}$$

Recalling the Taylor series expansion for the exponential function, $e^x = \sum_{n=0}^{\infty} \frac{x^n}{n!}$. Letting $x = \lambda e^{-\alpha}$, the summation becomes $e^{\lambda e^{-\alpha}}$.

$$T_0 = e^{-\lambda} \cdot e^{\lambda e^{-\alpha}}$$

Combining the exponents:

$$T_0 = e^{-\lambda + \lambda e^{-\alpha}} = e^{-\lambda(1-e^{-\alpha})}$$

*Q.E.D.*

**Corollary: Transmittance of $N$ Layers**
For a system composed of $N$ identical, independent layers, the total transmittance $T$ is the product of the transmittance of each individual layer.

$$T = (T_0)^N$$

Substituting the result from Proposition 3:

$$T = \left(e^{-\lambda(1-e^{-\alpha})}\right)^N$$

$$T = e^{-N\lambda(1-e^{-\alpha})}$$

This result indicates that the transmittance decays exponentially with the number of layers $N$, consistent with the macroscopic Beer-Lambert law. Because CNT films can be continuously thickened even after they become fully opaque, this model is constrained to the thin film regime.

5. Relationship Between Areal Coverage and Transmittance

Combining Proposition 1 and Proposition 3, the areal coverage $\phi$ can be expressed as a direct function of the total transmittance $T$:

$$\phi = 1 - T^{\beta}$$

where $\beta = \frac{1}{1-e^{-\alpha}}$ depends solely on the absorption of a single tube.

6. Physical Interpretation and Estimation of $\beta$ for Few-Walled Carbon Nanotubes

The average diameter of carbon nanotubes in this work is $4.36\ \mathrm{nm}$ (few-walled CNT), which typically accommodates approximately 3 concentric walls ($N_{walls} \approx 3$ ). The theoretical absorptance of a single free-standing graphene layer is $\pi\alpha_{fine} \approx 2.3\%$ . Thus, the total absorptance fraction for one tube is approximately

$$1 - e^{-\alpha} \approx 6 \times 0.023 = 0.138\ =\ 13.8\%$$

And exponent β:

$$\beta = \frac{1}{1 - e^{-\alpha}} \approx \frac{1}{0.138} \approx 7.25$$

Fitting the Boolean model to experimental data yields $\beta = 6.23\ \pm 1.51$ (95% CI), in agreement with the theoretical prediction, confirming the physical interpretation of $\beta$ as arising from the intrinsic optical absorption of few-walled CNTs.

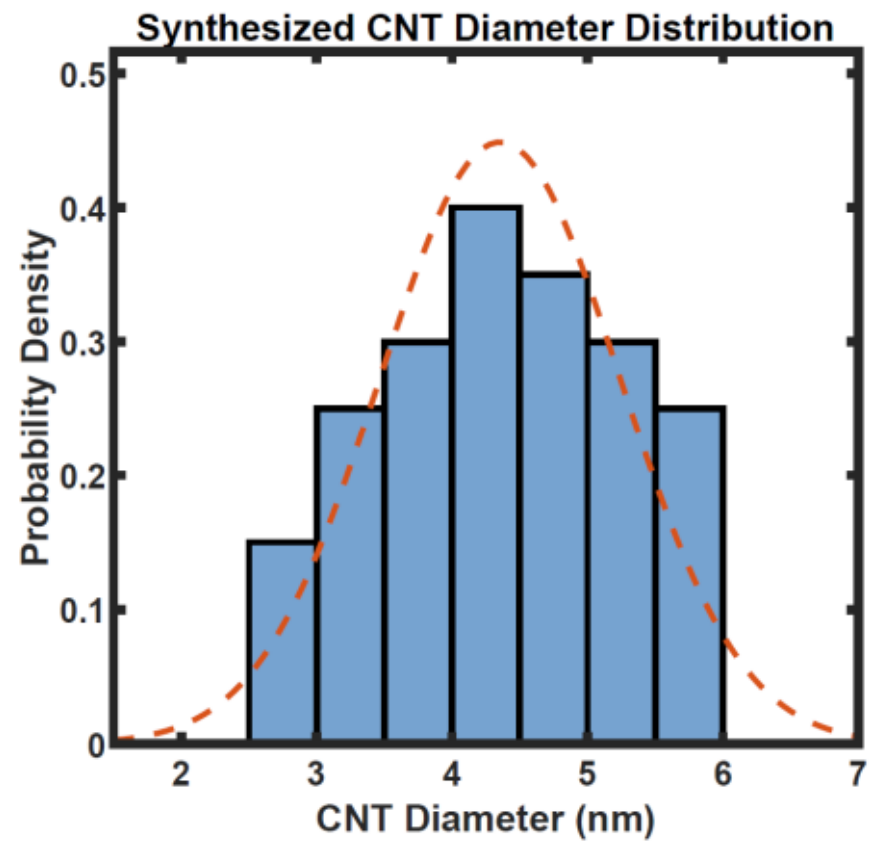


**Supplementary Figure S11 |** Diameter distribution of the synthesized CNTs.

Histogram of CNT diameters fitted with a Gaussian distribution, showing a mean diameter of 4.36 nm and a standard deviation of σ = 0.212.

**Supplementary Note S4 |** Optical transmittance as a function of sheet resistance.
Optical transmittance as a function of sheet resistance can be fitted using the classic transparent-conductor equation in literature[36].

$$T = \left(1 + \frac{Z_0}{2R_\mathrm{S}} \frac{\sigma_\mathrm{DC}}{\sigma_\mathrm{OP}}\right)^{-2}$$

where $Z_0$ is the impedance of free space, $\sigma_{DC}$ is electrical conductivity, and $\sigma_\mathrm{OP}$ is the optical conductivity. Fitting this expression to the experimental data yields a value of $\sigma_\mathrm{DC}/\sigma_\mathrm{OP} \approx 12.95$, which is consistent with commonly reported figures of merit for transparent conductors.

The CNT film in this work differs from typical percolative materials because it inherently meets a percolation requirement during synthesis. Since the process requires drawing a continuous film directly from the reactor, the CNT network must already be sufficiently interconnected for continuous collection. As a result, the prevalence of disconnected bundles, dead-end paths, or other non-conductive network fragments is naturally lower than in conventional percolative systems.

**Supplementary Notes S5 |** Conformability Model on Skin.

To model the conformability of the CNT thin film on skin, a mechanical model (Wang-Lu Model) which describes the partial conformability of thin elastic membranes on wavy surfaces was adopted[32]. The total energy of the system is expressed as:

$$U_{\text{total}} = U_{\text{bending}} + U_{\text{membrane}} - U_{\text{adhesion}} + U_{\text{substrate}}$$

Since the skin deformation due to application of the CNT thin film is negligible, $U_{\text{substrate}}$, the energy associated with deforming the substrate itself is assumed to be zero.

The expanded and normalized energy expression of the model becomes:

$$\widehat{U} = \alpha \frac{1}{12}\eta^3 D(\hat{x}_c) + \alpha\eta\beta^2 K(\hat{x}_c, \beta) - \frac{\mu}{\beta^2} E(\hat{x}_c, \beta)$$

The functions $D(\hat{x}_c)$, $K(\hat{x}_c, \beta)$, and $E(\hat{x}_c, \beta)$ describe the bending, membrane, and adhesion energy contributions, respectively, as a function of the fractional conformal coverage $\hat{x}_c$.Readers are referred to the original Wang and Lu manuscript[32] for the complete functional forms. For consistency with our notation, we refer to this fractional coverage as $\varphi$.

Four parameters govern this model are (1) Modulus ratio $\alpha = E_{\mathrm{m}}/E_{\mathrm{s}}$, where $E_{\mathrm{m}}$ and $E_{\mathrm{s}}$ are the Young's moduli of the membrane and substrate; (2) Normalized roughness $\beta = (2\pi h_0)/\lambda$, where $h_0$ and $\lambda$ are the amplitude and wavelength of the sinusoidal surface profile; (3) normalized membrane thickness $\eta = t/\lambda$, where $t$ is the membrane thickness; and (4) normalized work of adhesion $\mu = \gamma/(E_{\mathrm{s}}\lambda)$, where $\gamma$ is the adhesion energy of the CNT film obtained from literature.

This energy formulation yields the conformal area $\varphi$ that minimizes the total energy. A negative total energy indicates that adhesion stabilizes a given conformal configuration on a sinusoidal surface.

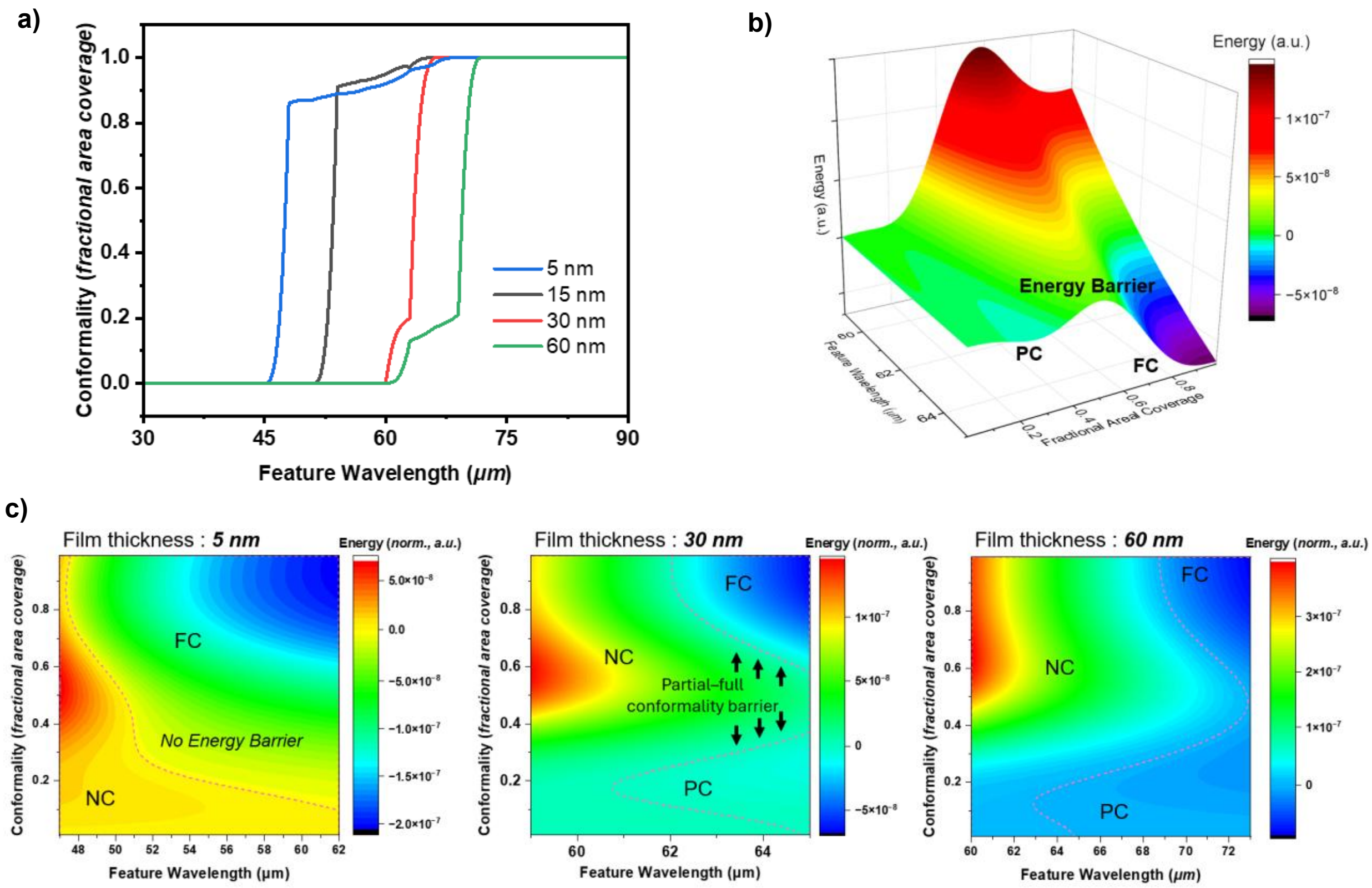


**Supplementary Figure S12** | Energy landscape of conformability states.

**a,** Fractional area coverage (conformability) as a function of skin feature wavelength for CNT films with thicknesses of 5, 15, 30, and 60 nm, showing a sharp onset of conformal coverage. The threshold feature wavelength increases with CNT film thickness as expected. **b,** Representative three-dimensional energy landscape illustrating the total energy of the system as a function of feature wavelength and conformability. Three regimes are identified based on the location of the global energy minimum: non-conforming (NC), partial conformal (PC), and full conformal (FC). **c,** Two-dimensional energy maps for films with thicknesses of 5 nm (left), 30 nm (center), and 60 nm (right). In each plot, the pink dotted line marks the wavelength at which the total energy first becomes negative and is energetically favorable. Each film exhibits a characteristic sequence of transitions (NC-PC-FC) occurring at distinct wavelength intervals, with corresponding onset points shown in **a**. Notably, films of 30 nm and 60 nm thickness display clear conformational transition barriers separating these regimes.

However, real skin exhibits regions that are relatively flat as well as deeper valleys and are better represented by a spectrum of spatial frequencies, each contributing a distribution of local curvatures, while the Wang–Lu model assumes that the substrate surface is purely sinusoidal. In experiments, conformability appears to saturate, suggesting that skin wrinkles include significant high-curvature features that fall outside the sinusoidal assumption of the model. To more accurately capture this behavior, a sigmoidal model was used instead:

$$\varphi = M_{\mathrm{v}} + \frac{M_{\mathrm{f}} - M_{\mathrm{v}}}{1 + (T/T_0)^p}$$

where $p$ is the shape parameter, and $T_0$ is the midpoint of the sigmoid. The terms $M_{\mathrm{f}}$ and $M_{\mathrm{v}}$ represent the conformability limits associated with the flat and valley-dominated regions of the surface, respectively. This form naturally captures two regimes: a strongly conforming regime at high transmittance $T$, and a weakly conforming regime at low $T$.

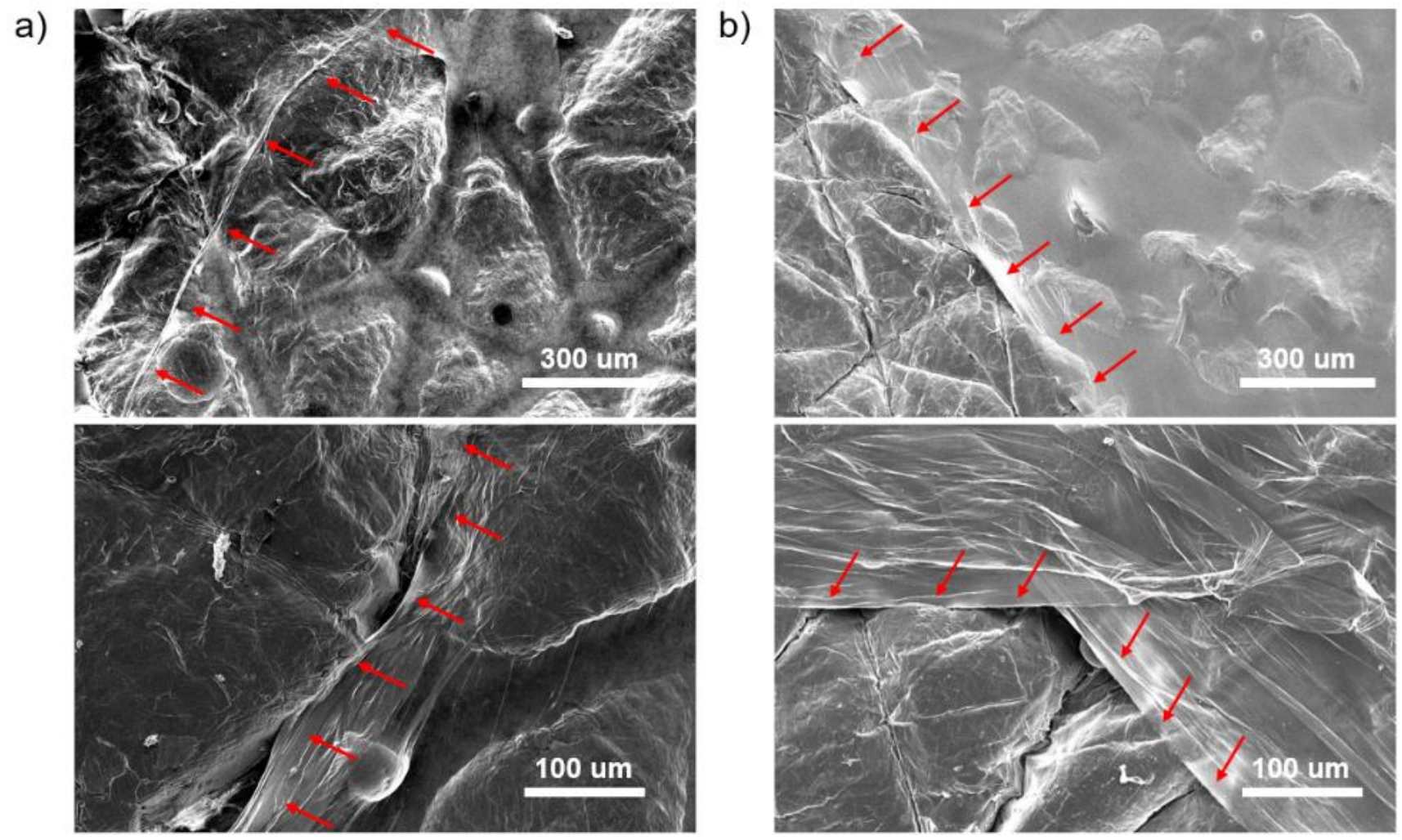


**Supplementary Figure S13 | CNT film conformability vs film thickness.**

SEM images of CNT films with ~60%*T* (a) and ~30%*T* (b) on skin replica. the skin replica is only partially covered by the CNT films and the red arrows indicate the transition between CNT-covered and uncovered regions. Scale bars: 300 µm and 100 µm.

The ~60%*T* CNT film more closely follows the native skin microtopography, conforming to surface wrinkles and ridges. In contrast, the thicker, denser ~30%*T* CNT film appears less conformal, spanning across and partially masking the underlying surface features like a blanket over the skin texture. Red arrows highlight the film–skin interface and regions where differences in conformability are evident. These images show that increasing CNT film thickness reduces conformability to microscale skin features.

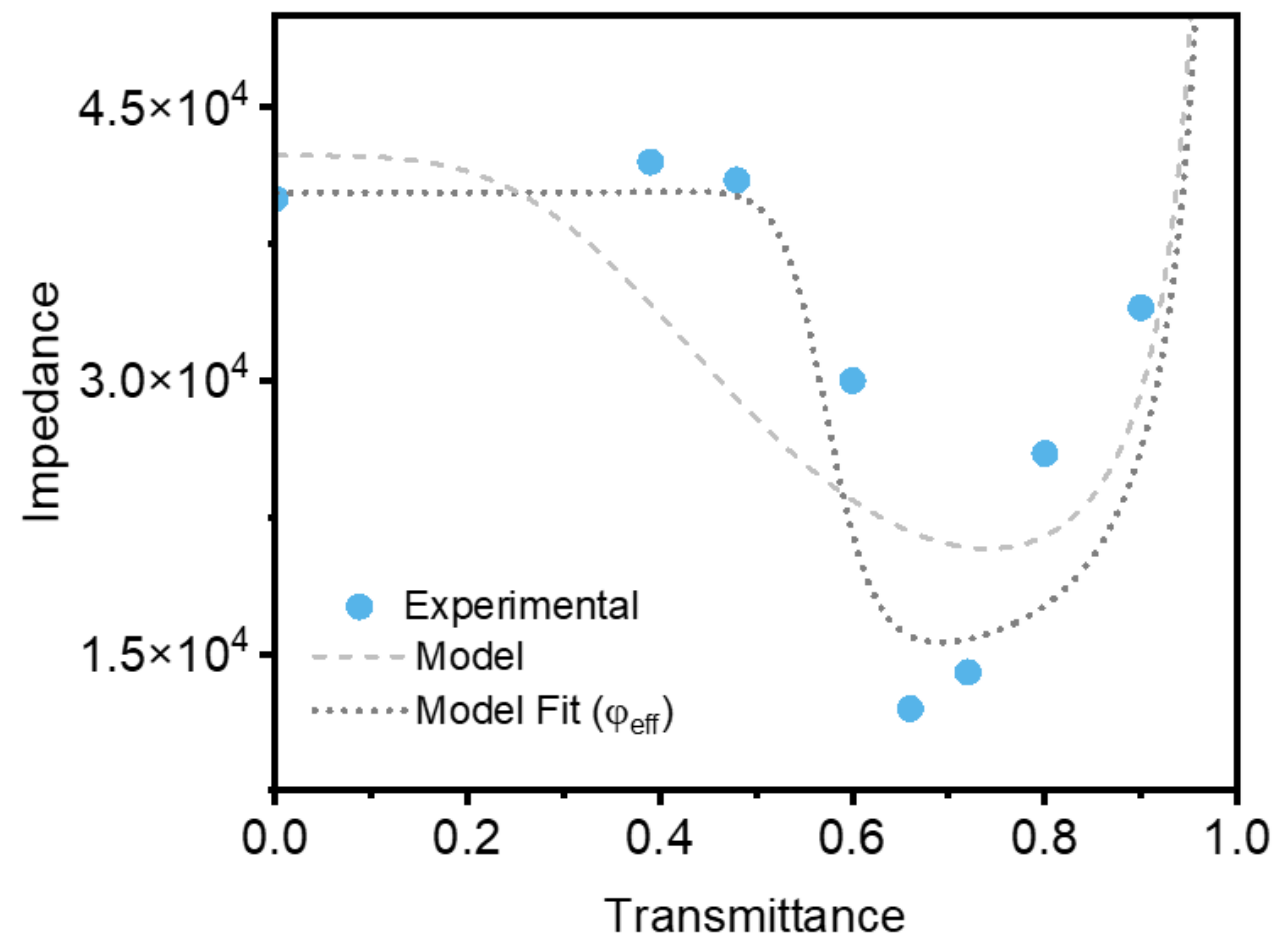


**Supplementary Figure S14 |** Comparison of contact impedance model with experimental data.

Measured contact impedance as a function of optical transmittance (blue symbols). The dashed line shows the contact impedance model prediction, while the dotted line shows the model fit with fitted conformability parameters.

With these models established, two factors become critical for predicting electrical performance. The effective conformal area fraction is defined as the product of areal coverage and conformability:

$$\varphi_{\text{eff}} = \varphi \times \phi$$

The normalized contact impedance can then be expressed using a classic electrode–skin interface (ESI) model, given by

$$Z(\omega, T) = R_{\text{S}}(T) + \left(\frac{\varphi_{\text{eff}}(T)}{R_{\text{ct}}} + \varphi_{\text{eff}}(T) \cdot Y_0 \cdot (j\omega)^{\alpha}\right)^{-1}$$

In the low-frequency limit, the impedance approaches

$$Z_{\text{DC}}(T) = R_{\text{S}}(T) + \frac{R_{\text{ct}}}{\varphi_{\text{eff}}(T)}$$

While the combined model based on sheet resistance, areal coverage, and conformability captures the overall trends from experimental results, quantitative deviations are observed. These deviations primarily arise from differences between the projected conformal area measured from electron micrographs of CNT films transferred onto replica substrates and the effective conformability on human skin. Such discrepancies are expected due to the substantially different Young's modulus of human skin compared to the replica substrate, as well as variations in transfer mechanics during CNT film application. Consequently, parameters extracted from replica-based conformability measurements may not fully capture the absolute behavior on skin, although the trends remain representative. By adjusting the conformability limit and the shape parameter of the conformability model, while holding all other parameters and the overall model formulation constant, the model achieves improved quantitative agreement with the experimental results.

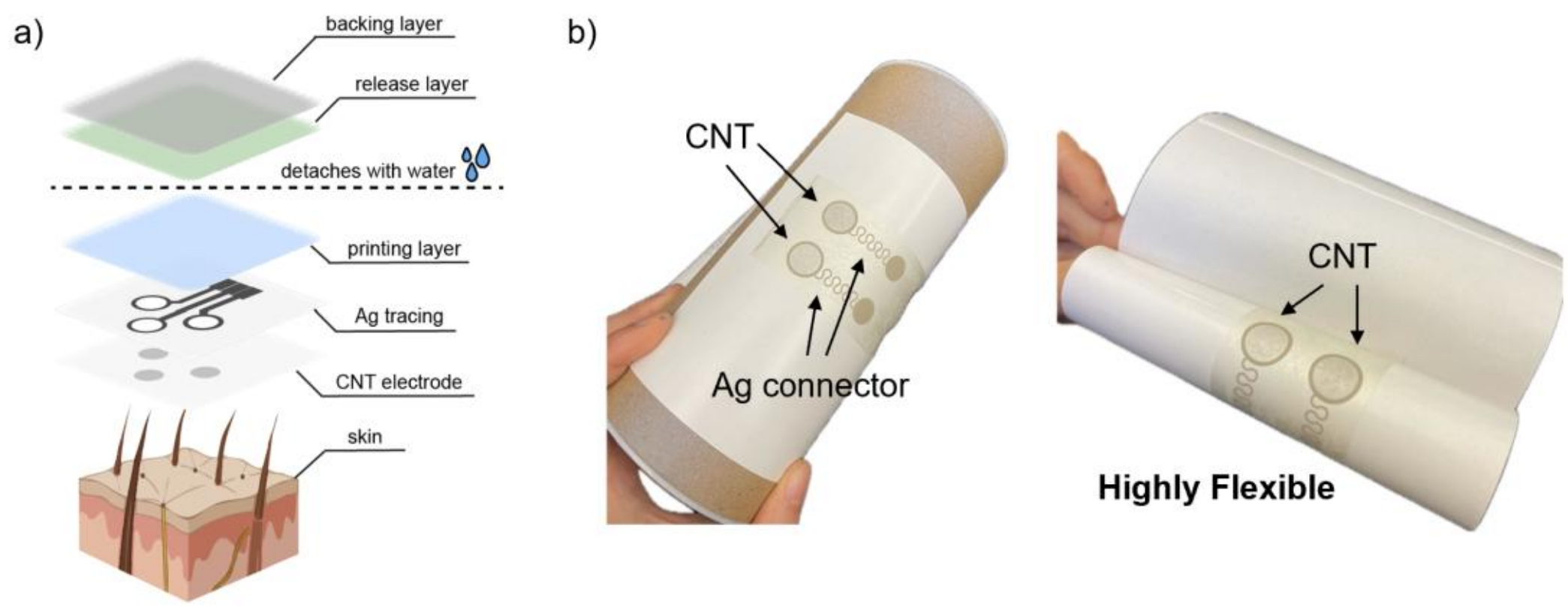


**Supplementary Figure S15 |** Device stack of CNT dry electrodes for epidermal electrophysiology. **a,** Schematic illustration of the electrode stack and water-assisted transfer process. The temporary transfer medium consists of a backing layer, water-dissolvable release layer, and printing layer that supports patterned CNT electrodes and printed Ag interconnects during device assembly and transfer to skin. **b,** Photographs of patterned CNT electrodes and Ag connectors, showing high mechanical flexibility.

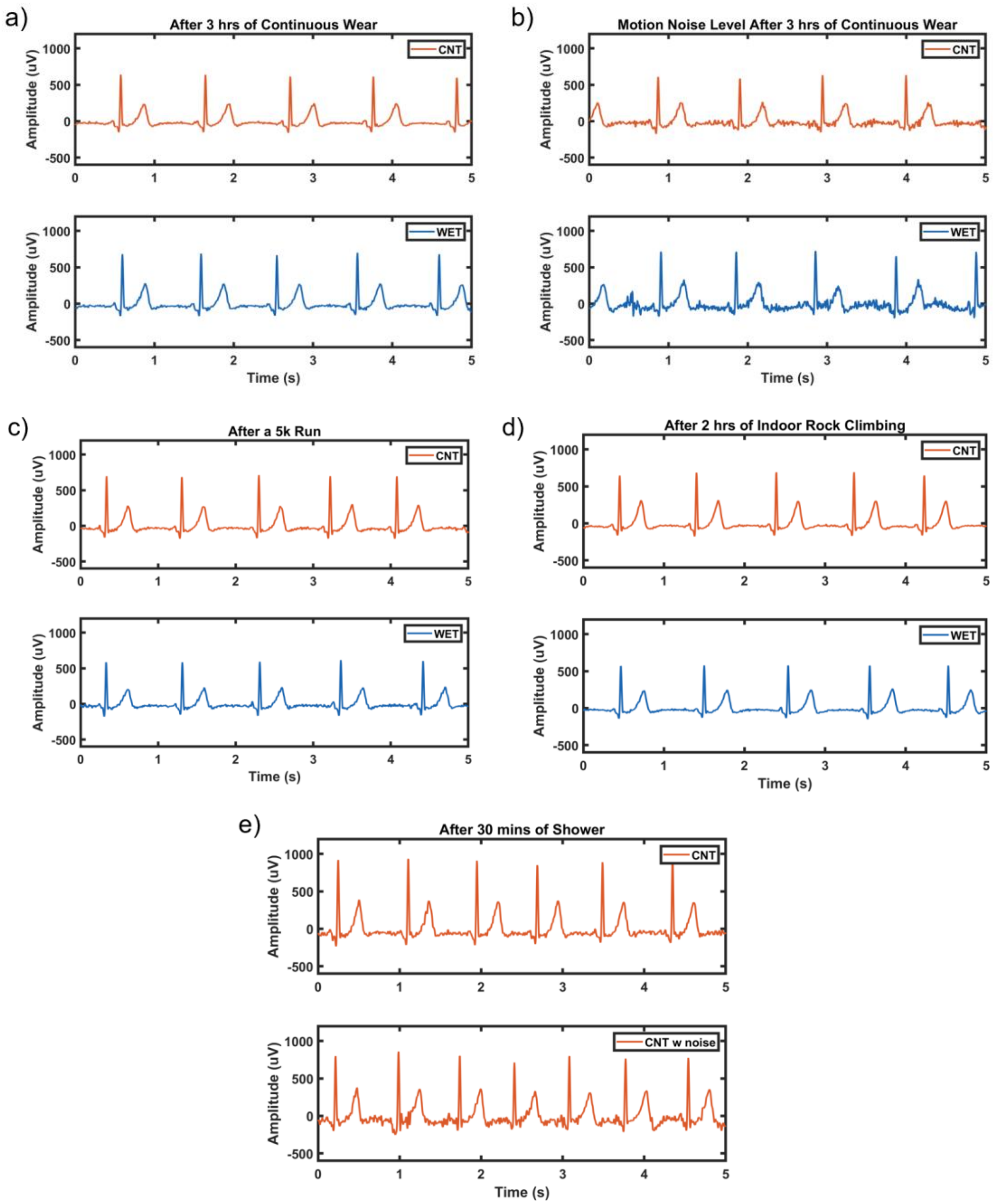


**Supplementary Figure S16 |** ECG signal stability after sweat, exercise, and shower.

**a,** ECG signals after 3 h of continuous wear. **b,** ECG recordings during motion perturbation following 3 h of continuous wear, showing motion-noise response. **c,** ECG signals recorded after an outdoor 5 km run, used to evaluate electrode performance after heavy sweating. **d,** ECG signals recorded after 2 h of indoor rock climbing, used to evaluate stability following repeated upper-body motion, forearm muscle activation, and mechanical perturbation. **e,** CNT ECG signals recorded after a 30 min hot shower, used to evaluate performance after elevated skin temperature and extreme external moisture exposure. Wet electrodes had lost adhesion during the hot shower.

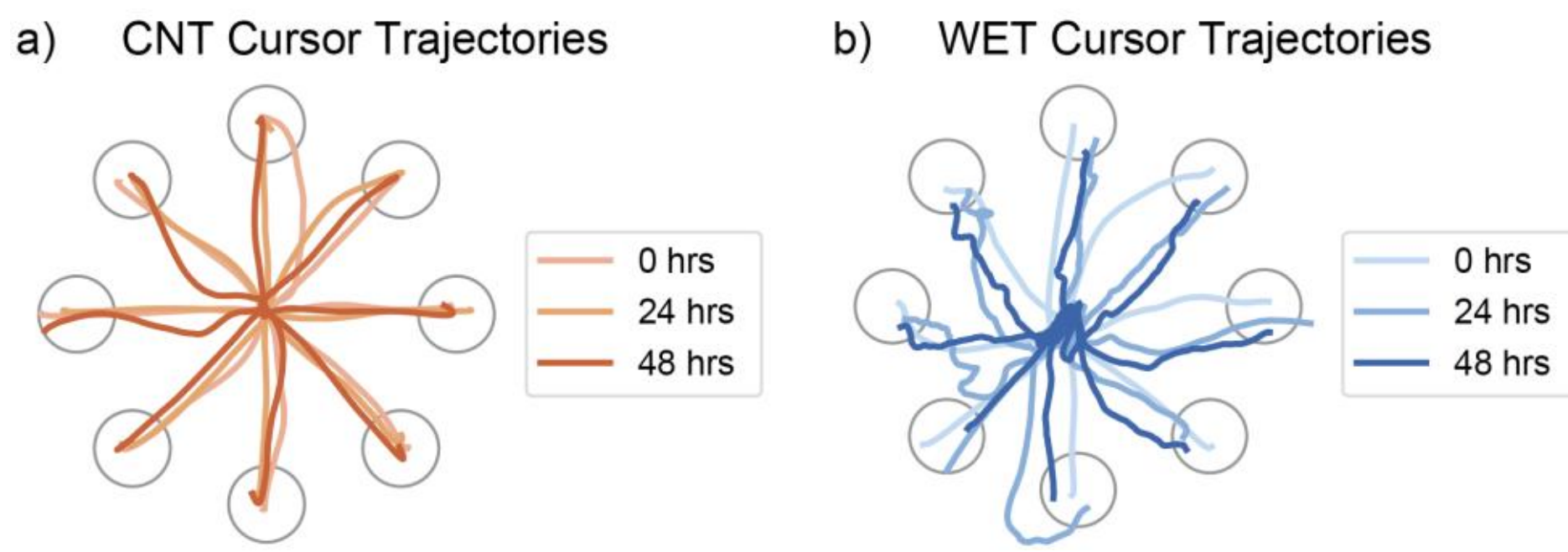


**Supplementary Figure S17 |** Cursor trajectory stability over time.

Representative cursor trajectories for eight-direction center-out tasks recorded using **(a)** CNT dry electrodes and **(b)** commercial gel electrodes at 0 h, 24 h, and 48 h. Each trace represents the average trajectory from the center to one of eight peripheral targets. CNT electrodes maintain consistent and well-directed trajectories across all sessions without decoder recalibration, whereas commercial electrodes show trajectory drift and reduced directional consistency over time.

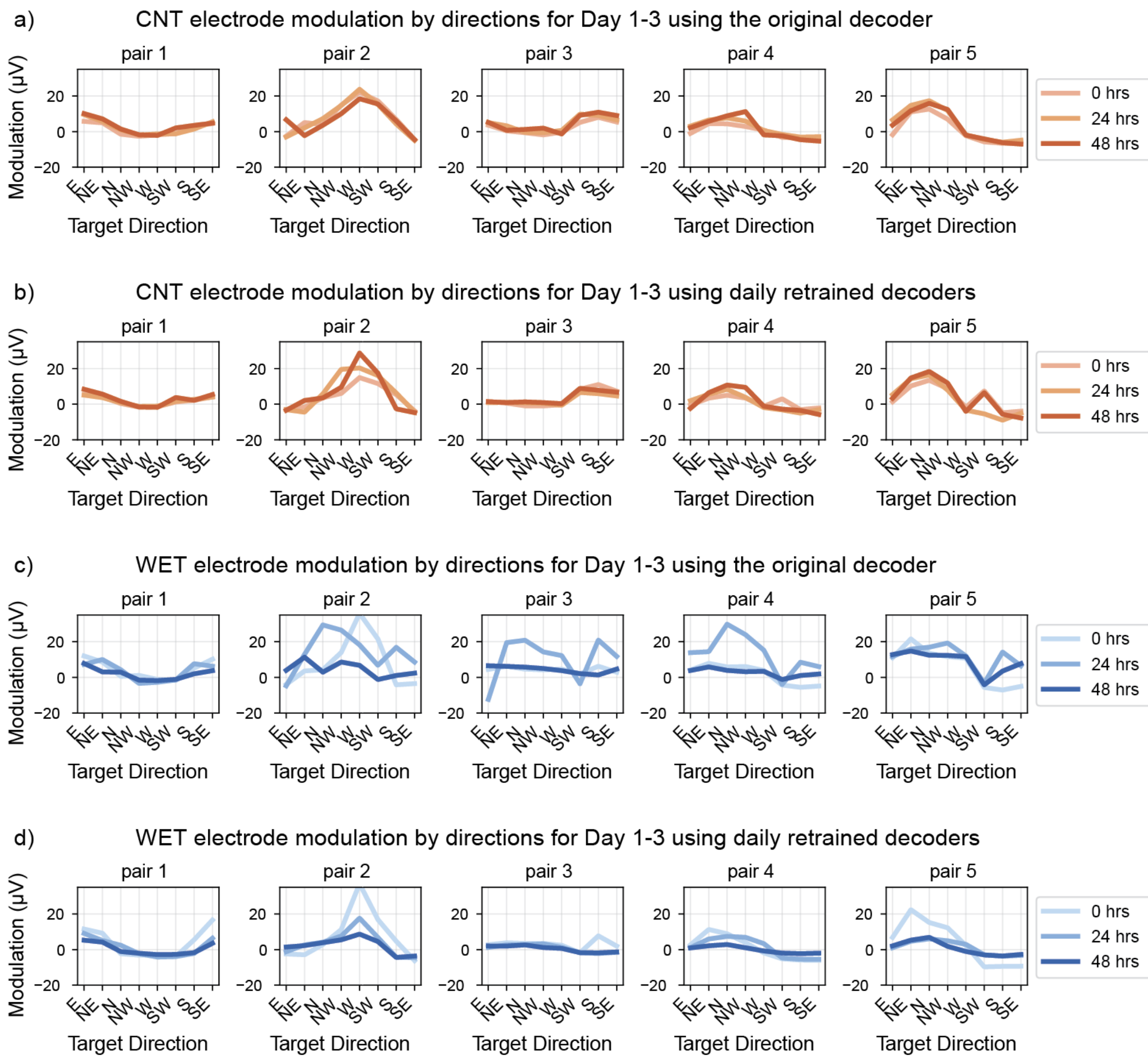


**Supplementary Figure S18 |** Tuning curve stability over time.
**(a–d)** Directional tuning curves for five electrode pairs measured using (a,b) CNT dry electrodes and (c,d) wet electrodes at 0 h, 24 h, and 48 h. (a,c) show modulation profiles obtained with the original decoder, while (b,d) show curves generated after decoder retraining. CNT electrodes maintain consistent modulation patterns across sessions without retraining, whereas wet electrodes exhibit substantial variability requiring recalibration.

Although retraining at 48 h still restored BMI performance for wet electrodes, it did so with reduced response amplitudes. This supports instability, rather than reduced SNR alone, as the key constraint and underscores the impracticality of frequent recalibration for continuous-use systems. Hence, frequent recalibration becomes necessary to maintain performance, which is impractical for real-world, continuous-use systems.

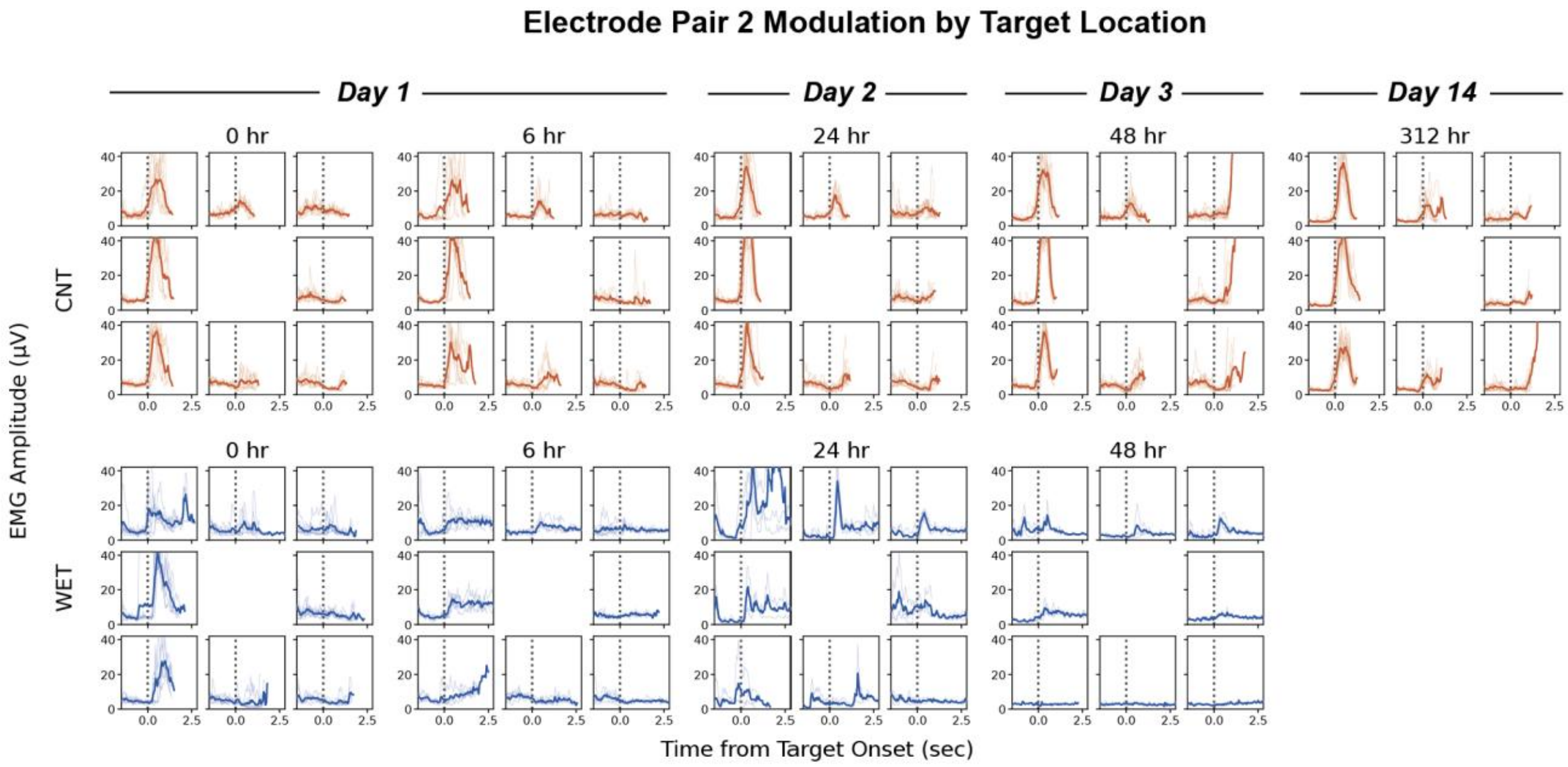


**Supplementary Figure S19 |** EMG modulation from electrode pair 2 over extended wear, grouped by target location.

EMG RMS traces from electrode pair 2 are shown for CNT dry electrodes and wet gel electrodes across cursor control sessions at 0, 6, 24, 48, and 312 h after initial electrode placement. Wet electrodes failed after 48 hours. Each small panel corresponds to one target location, arranged according to the center-out target layout. CNT electrodes preserved clear target-dependent modulation from Day 1 to Day 14, whereas wet electrodes showed significant variation in modulation patterns from Day 1 to Day 3. Dark traces represent the average values, and lighter traces represent individual trials. The vertical dashed line marks target onset.

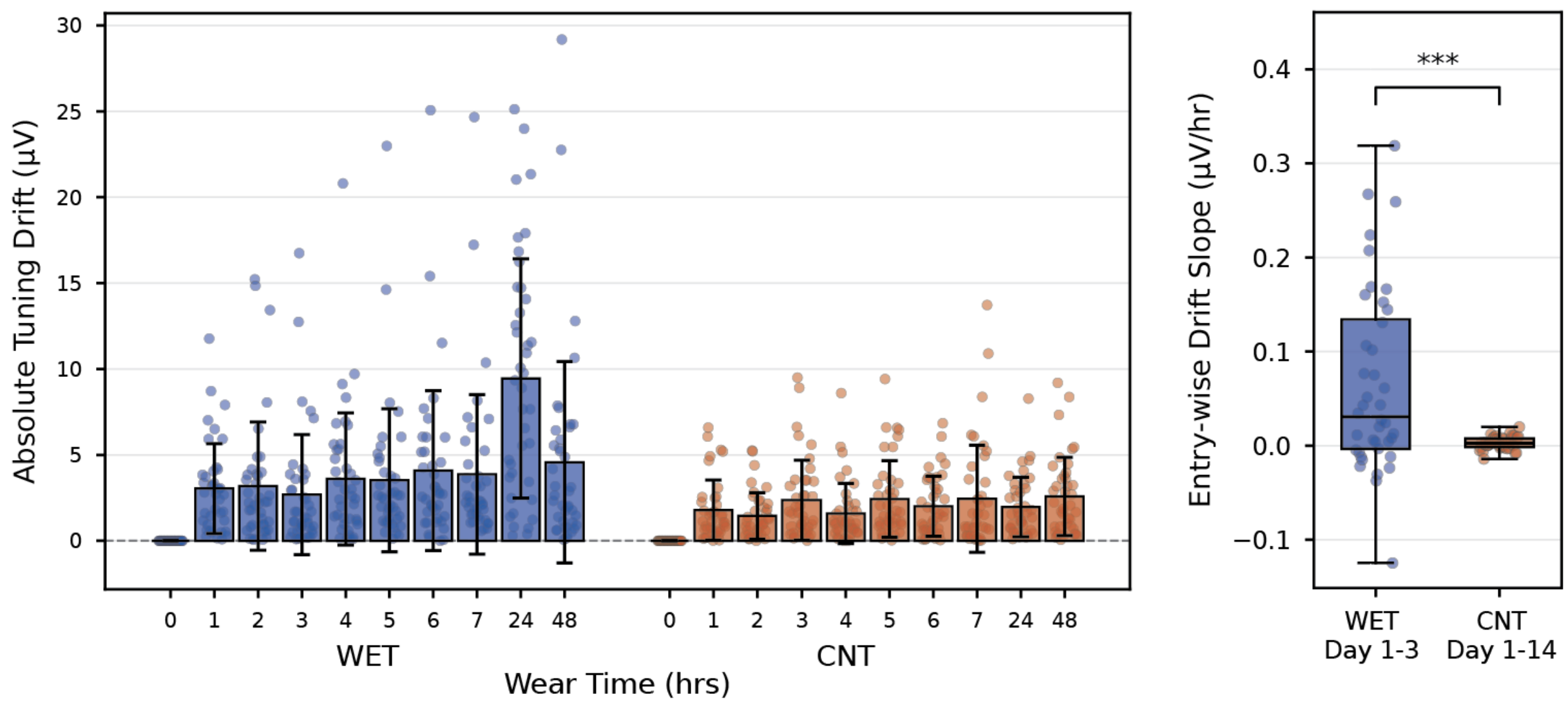


**Supplementary Figure S20 |** EMG tuning drift during extended wear.

Left, absolute EMG tuning drift was quantified over time for wet gel electrodes and CNT dry electrodes. Points represent individual measurements. Wet electrodes showed larger and more variable tuning drift over the first 48 h, whereas CNT electrodes maintained lower drift. Right, tuning drift slopes were compared for wet gel electrodes and CNT dry electrodes. CNT electrodes were calculated across 2 weeks of wear whereas the wet electrodes were measured across 2 days and failed afterwards. CNT electrodes exhibited significantly smaller drift slopes over Day 1–14 than wet electrodes over Day 1–3, indicating improved stability of target-dependent EMG tuning during prolonged wear.

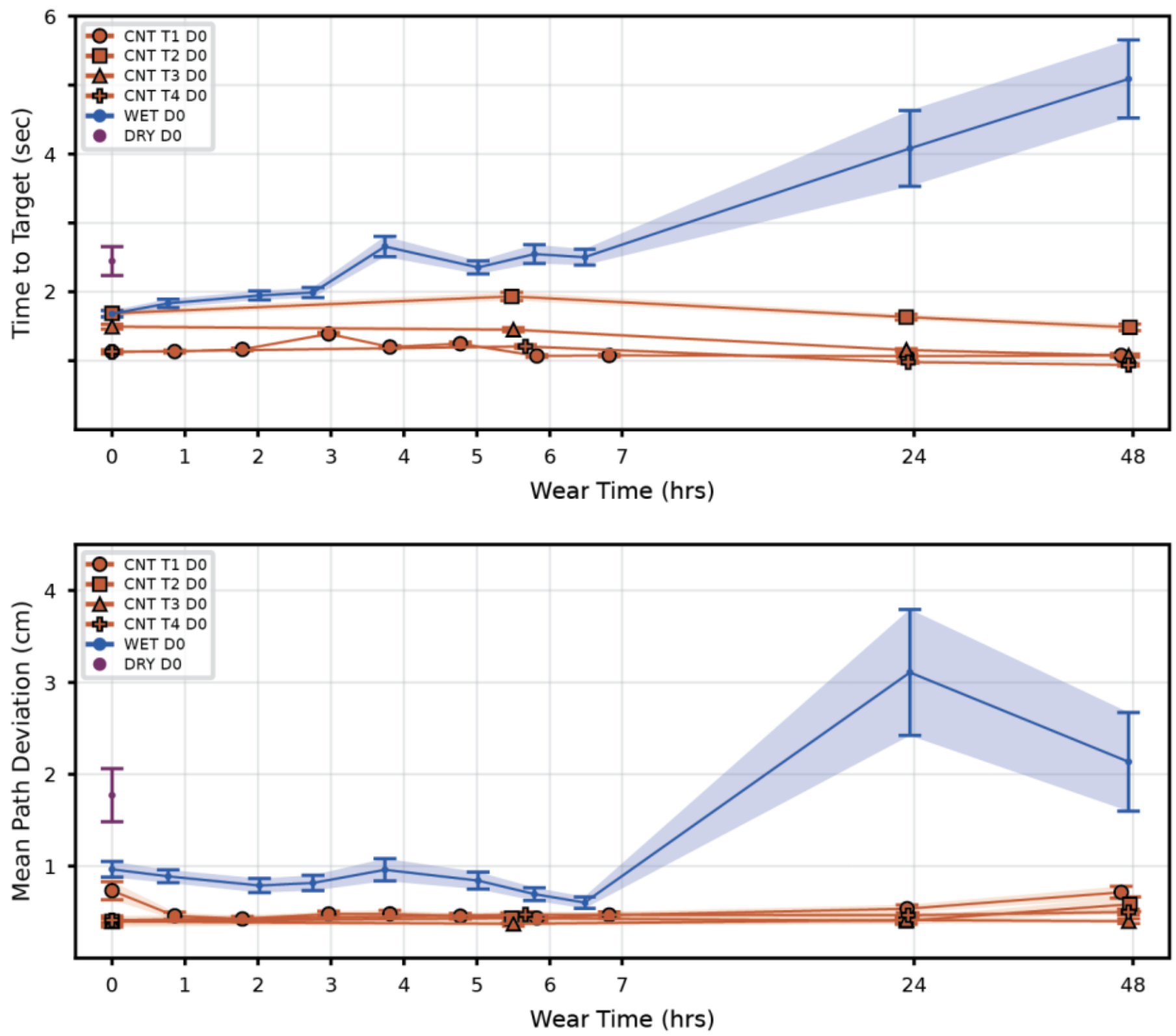


**Supplementary Figure S21 |** Reproducibility across independent CNT electrode sessions. The center-out task was evaluated using four independent CNT electrode sessions (T1–T4), with a new CNT electrode set applied for each session. Performance was quantified by time to target and mean path deviation over 48 h of wear and compared with wet and dry electrodes. Both performance metrics, time to target and path deviation, were consistent across all four CNT sessions, demonstrating reproducibility of the results.

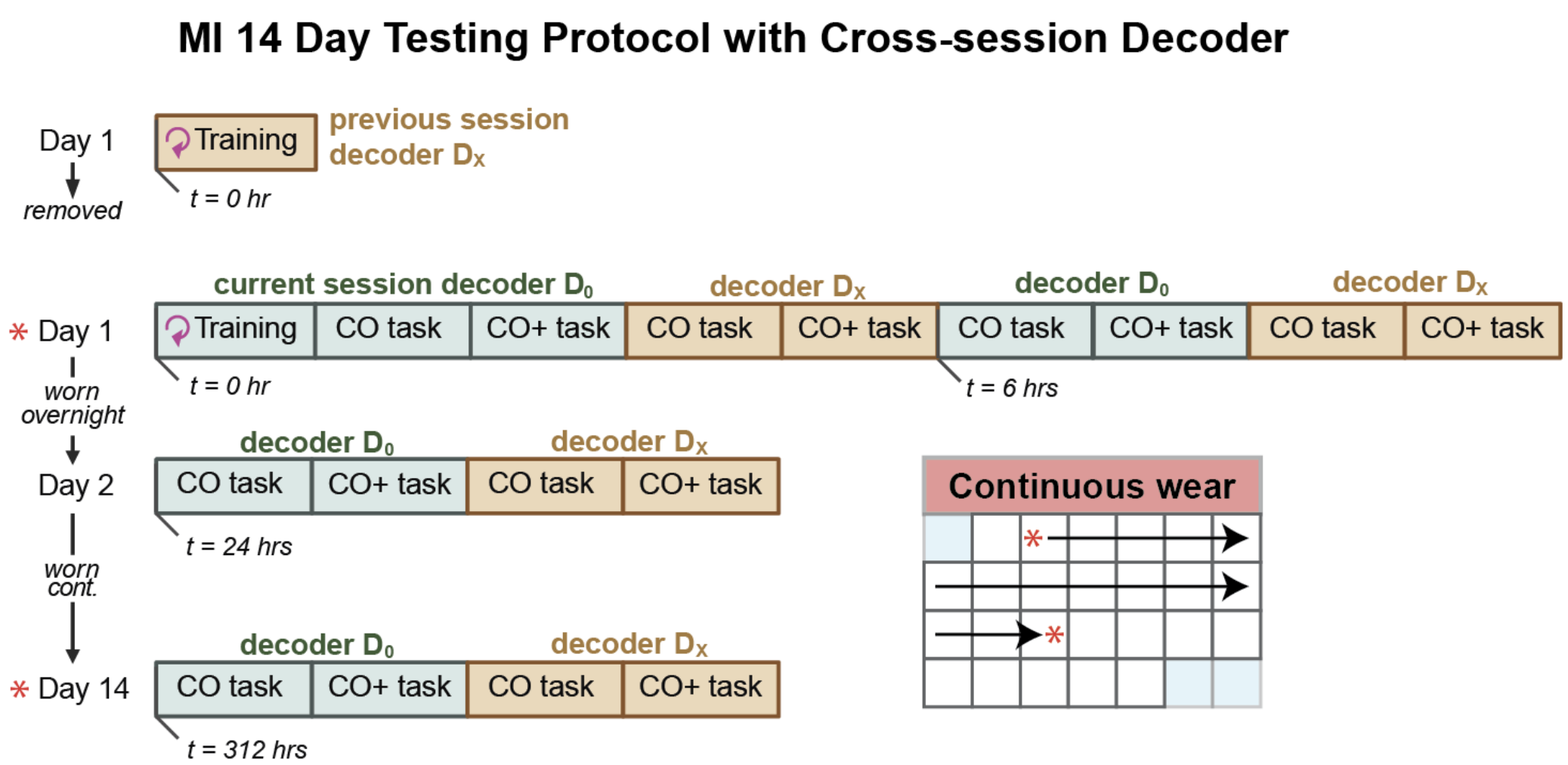


**Supplementary Figure S22 |** Experimental protocol for cross-session decoder transfer and performance comparison with retrained decoder for 14 days of continuous wear.

A 14-day myoelectric interface testing protocol is used to evaluate whether a decoder trained in one session could be applied in subsequent sessions without retraining. A decoder from a previous session ($D_x$) was first obtained in a separate earlier session. In the 14-day continuous-wear experiment, a new decoder ($D_0$) was trained at the start of Day 1 ($t = 0\ h$), after which participants performed the center-out (CO) and center-out with obstacle avoidance (CO+) tasks using both the current-session decoder ($D_0$) and the previous-session decoder ($D_x$). On Day 1, testing was repeated again at $t = 6\ h$. The same CO and CO+ evaluations with both decoders were then performed on Day 2 ($t = 24\ h$) and Day 14 ($t = 312\ h$) while the electrodes remained in continuous wear between sessions.

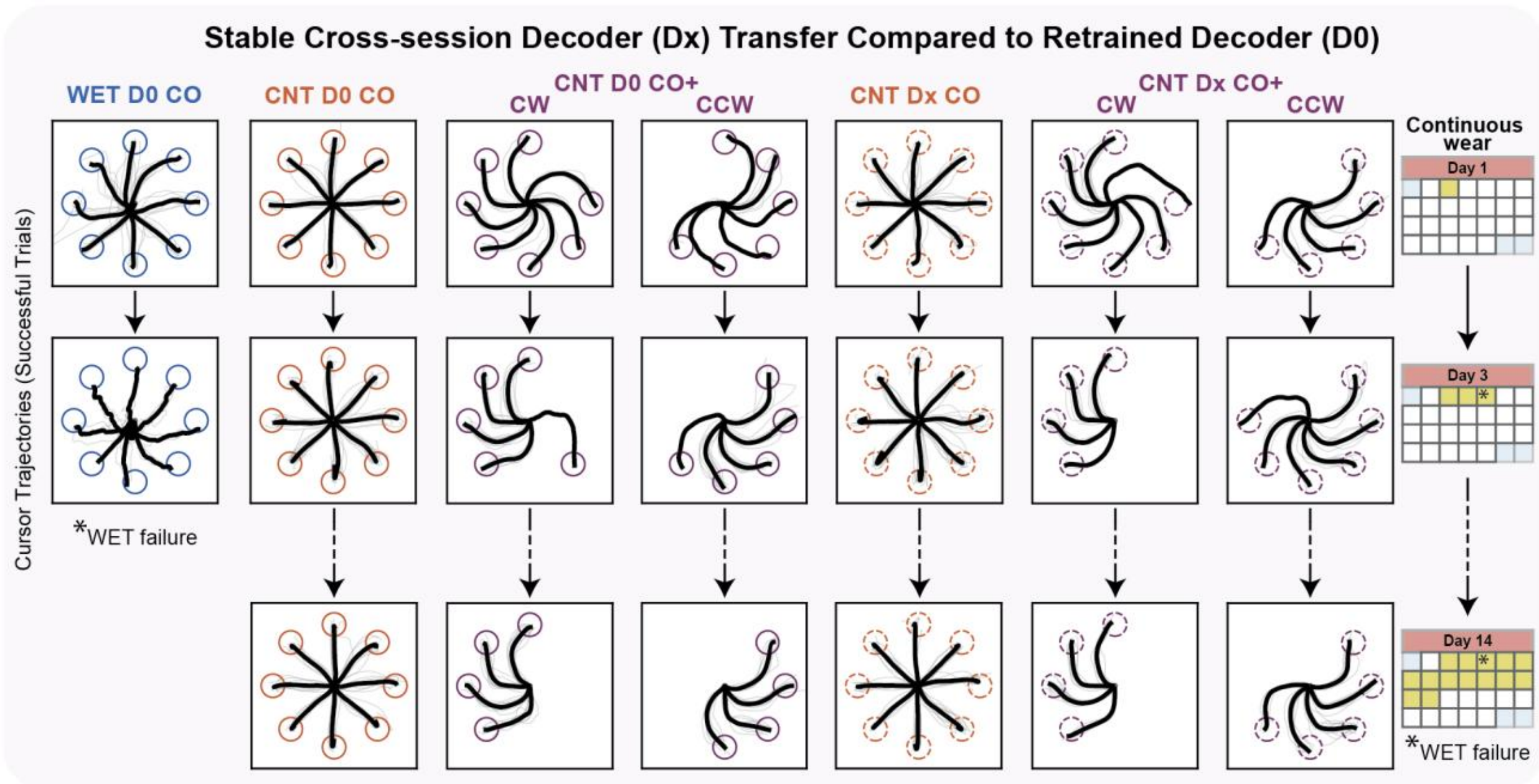


**Supplementary Figure S23 |** Cross-session decoder can be stably transferred to new sessions and supports stable closed-loop control over 14 days of continuous wear.

Cursor trajectories from successful trials in the center-out (CO) and center-out with obstacle avoidance (CO+) tasks using either a retrained current-session decoder ($D_0$) or a decoder transferred from a previous session ($D_x$). CO+ trajectories are shown separately for clockwise (CW) and counterclockwise (CCW) movements, because of the two possible paths around the obstacle between the home position and the target, depending on users' choice. CNT electrodes maintained directed and well-defined trajectories with both $D_0$ and $D_x$ throughout the two-week testing period, supporting stable performance in the CO task as well as in both CW and CCW CO+ tasks. Gray traces indicate individual successful trials; black traces indicate average trajectories.

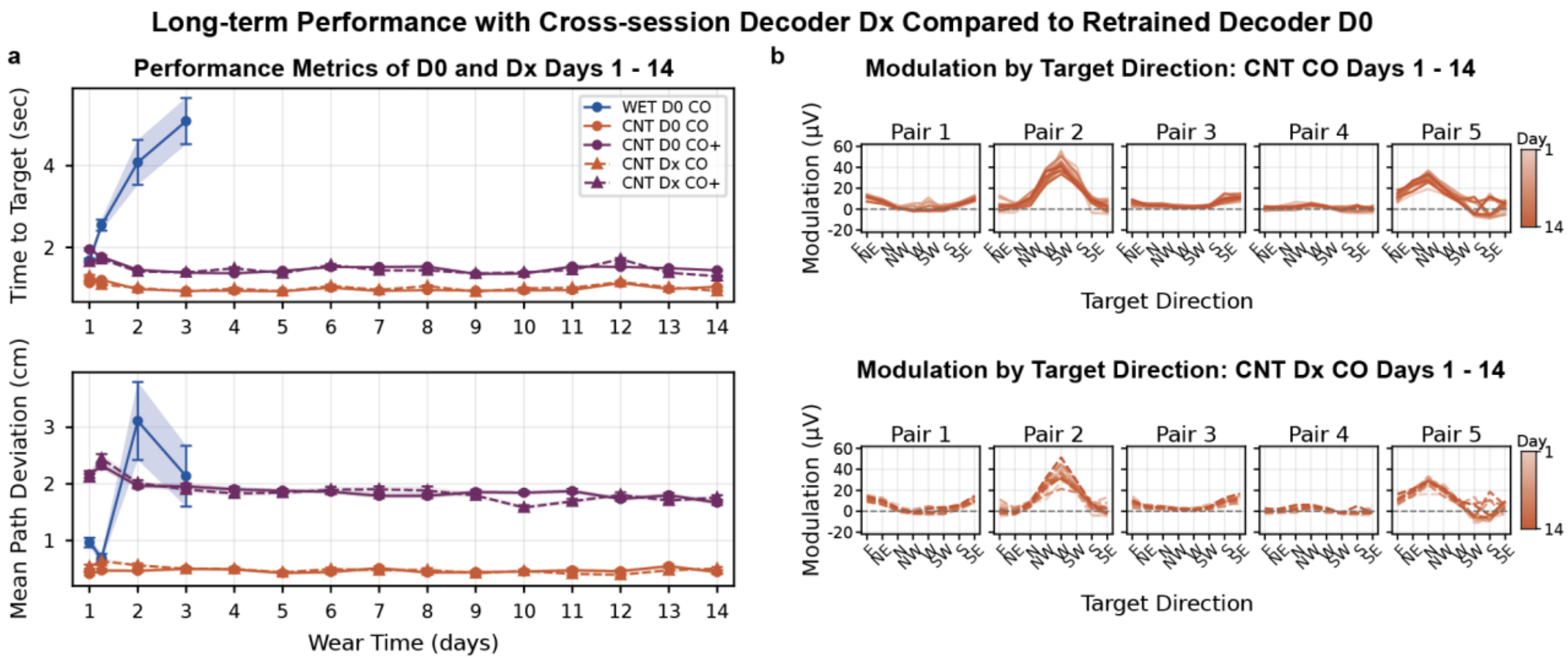


**Supplementary Figure S24 |** Cross-session decoder transfer maintains stable myoelectric interface performance and EMG modulation curves by target direction over 14 days of continuous CNT electrode wear.

**a,** Myoelectric interface performance metrics, time to target and mean path deviation, in the center-out (CO) and center-out with obstacle avoidance (CO+) tasks using either a retrained current-session decoder ($D_0$) or a cross-session decoder transferred from a previous session ($D_x$). CNT electrodes maintained stable time-to-target and path-deviation performance over 14 days, and performance with the transferred decoder $D_x$ remained comparable to the retrained decoder $D_0$.

**b,** EMG modulation curves by target directions from five CNT electrode pairs during CO tasks using both $D_0$ and $D_x$ across Days 1–14. Modulation patterns remained consistent across target directions and electrode pairs over the two-week testing period and the transferred decoder $D_x$ performed similarly to the retrained decoder $D_0$.

**Supplementary References:**


1. Lee, S. *et al.* Fabric-based lamina emergent MXene-based electrode for electrophysiological monitoring. *Nat. Commun.* **15**, 5974 (2024).
2. Driscoll, N. *et al.* MXene-infused bioelectronic interfaces for multiscale electrophysiology and stimulation. *Sci. Transl. Med.* **13**, eabf8629 (2021).
3. Yang, X. *et al.* All-Nanofiber-Based Janus Epidermal Electrode with Directional Sweat Permeability for Artifact-Free Biopotential Monitoring. *Small Weinh. Bergstr. Ger.* **18**, e2106477 (2022).
4. Yun, I. *et al.* Stable Bioelectric Signal Acquisition Using an Enlarged Surface-Area Flexible Skin Electrode. *ACS Appl. Electron. Mater.* **3**, 1842–1851 (2021).
5. Qiu, J. *et al.* A Bioinspired, Durable, and Nondisposable Transparent Graphene Skin Electrode for Electrophysiological Signal Detection. *ACS Mater. Lett.* **2**, 999–1007 (2020).
6. Jang, H. *et al.* Graphene e-tattoos for unobstructive ambulatory electrodermal activity sensing on the palm enabled by heterogeneous serpentine ribbons. *Nat. Commun.* **13**, 6604 (2022).
7. Hossain, M. M., Li, B. M., Sennik, B., Jur, J. S. & Bradford, P. D. Adhesive free, conformable and washable carbon nanotube fabric electrodes for biosensing. *Npj Flex. Electron.* **6**, 97 (2022).
8. Oh, J. *et al.* Flexible Dry Electrode Based on a Wrinkled Surface That Uses Carbon Nanotube/Polymer Composites for Recording Electroencephalograms. *Materials* **17**, (2024).
9. Jung, H.-C. *et al.* CNT/PDMS Composite Flexible Dry Electrodesfor Long-Term ECG Monitoring. *IEEE Trans. Biomed. Eng.* **59**, 1472–1479 (2012).
10. Gogurla, N., Kim, Y., Cho, S., Kim, J. & Kim, S. Multifunctional and Ultrathin Electronic Tattoo for On-Skin Diagnostic and Therapeutic Applications. *Adv. Mater.* **33**, 2008308 (2021).
11. Taccola, S. *et al.* Toward the Use of Temporary Tattoo Electrodes for Impedancemetric Respiration Monitoring and Other Electrophysiological Recordings on Skin. *Sensors* **21**, (2021).
12. Zhang, L. *et al.* Fully organic compliant dry electrodes self-adhesive to skin for long-term motion-robust epidermal biopotential monitoring. *Nat. Commun.* **11**, 4683 (2020).
13. Ferrari, L. M., Ismailov, U., Badier, J.-M., Greco, F. & Ismailova, E. Conducting polymer tattoo electrodes in clinical electro- and magneto-encephalography. *Npj Flex. Electron.* **4**, 4 (2020).
14. Ma, C. *et al.* Compliant and breathable electrospun epidermal electrode towards artifact-free electrophysiological monitoring. *Chem. Eng. J.* **490**, 151118 (2024).
15. Du, X. *et al.* Ultrathin Bioelectrode Array with Improved Electrochemical Performance for Electrophysiological Sensing and Modulation. *ACS Nano* **18**, 34971–34985 (2024).
16. Weder, M. *et al.* Embroidered Electrode with Silver/Titanium Coating for Long-Term ECG Monitoring. *Sensors* **15**, 1750–1759 (2015).
17. Wang, Y. *et al.* Electrically compensated, tattoo-like electrodes for epidermal electrophysiology at scale. *Sci. Adv.* **6**, eabd0996 (2020).

18. Xie, R. *et al.* Strenuous exercise-tolerance stretchable dry electrodes for continuous multi-channel electrophysiological monitoring. *Npj Flex. Electron.* **6**, 75 (2022).
19. Nawrocki, R. A. *et al.* Self-Adhesive and Ultra-Conformable, Sub-300 nm Dry Thin-Film Electrodes for Surface Monitoring of Biopotentials. *Adv. Funct. Mater.* **28**, 1803279 (2018).
20. Harimurti, S. *et al.* Janus electrode with stable asymmetric wettability for robust biosignal monitoring on sweaty skin. *Mater. Today* **74**, 94–108 (2024).
21. Liu, X. *et al.* Highly robust ECG electrodes constructed from semi-liquid metal fibers for reliable emergency rescue monitoring. *Npj Flex. Electron.* **10**, 56 (2026).
22. Xu, Y. *et al.* Porous liquid metal–elastomer composites with high leakage resistance and antimicrobial property for skin-interfaced bioelectronics. *Sci. Adv.* **9**, eadf0575 (2023).
23. Li, S. *et al.* Hundred-Nanometer-Thick Stretchable Liquid Metal Films for Ultra-Conformal Bioelectrodes. *Adv. Sci.* **13**, e12762 (2025).
24. Yang, G. *et al.* Adhesive and Hydrophobic Bilayer Hydrogel Enabled On-Skin Biosensors for High-Fidelity Classification of Human Emotion. *Adv. Funct. Mater.* **32**, 2200457 (2022).
25. Zhang, Z. *et al.* A 10-micrometer-thick nanomesh-reinforced gas-permeable hydrogel skin sensor for long-term electrophysiological monitoring. *Sci. Adv.* **10**, eadj5389 (2024).
26. Yang, G. *et al.* A Nepenthes-Inspired Hydrogel Hybrid System for Sweat-Wicking Electrophysiological Signal Recording during Exercises. *Adv. Funct. Mater.* **35**, 2417841 (2025).
27. Niu, X. *et al.* Fructus Xanthii-Inspired Low Dynamic Noise Dry Bioelectrodes for Surface Monitoring of ECG. *ACS Appl. Mater. Interfaces* **14**, 6028–6038 (2022).
28. Stauffer, F. *et al.* Skin Conformal Polymer Electrodes for Clinical ECG and EEG Recordings. *Adv. Healthc. Mater.* **7**, e1700994 (2018).
29. Kaveh, R., Schwendeman, C., Pu, L., Arias, A. C. & Muller, R. Wireless ear EEG to monitor drowsiness. *Nat. Commun.* **15**, 6520 (2024).
30. Fulem, M. *et al.* Recommended vapor pressure and thermophysical data for ferrocene. *J. Chem. Thermodyn.* **57**, 530–540 (2013).
31. West, W. A. & Menzies, A. W. C. The Vapor Pressures of Sulphur between 100° and 550° with related Thermal Data. *J. Phys. Chem.* **33**, 1880–1892 (1929).
32. Wang, L. & Lu, N. Conformability of a Thin Elastic Membrane Laminated on a Soft Substrate With Slightly Wavy Surface. *J. Appl. Mech.* **83**, (2016).
33. Meester, R. & Roy, R. *Continuum Percolation*. (Cambridge University Press, 1996).
34. Jeulin, D. Boolean Random Sets. in *Morphological Models of Random Structures* (ed. Jeulin, D.) 165–231 (Springer International Publishing, Cham, 2021). doi:10.1007/978-3-030-75452-5_6.
35. Hug, D., Last, G. & Weil, W. Boolean models. Preprint at https://doi.org/10.48550/arXiv.2308.05861 (2023).
36. De, S., King, P. J., Lyons, P. E., Khan, U. & Coleman, J. N. Size Effects and the Problem with Percolation in Nanostructured Transparent Conductors. *ACS Nano* **4**, 7064–7072 (2010).
