# Supplementary material for: Multi-day recalibration-free electrophysiology interface based on carbon nanotube thin film dry electrodes
Source: Nat Commun. 2026 Oct 13;17:11583. doi: 10.1038/s41467-026-78169-6 (PMC13751614; doi:10.1038/s41467-026-78169-6)
Supplement: Supplementary file 2 [file 41467_2026_78169_MOESM2_ESM.pdf]

<u>Description of Additional Supplementary Files</u>

Supplementary Movie 1 | Roll-to-roll collection of CNT thin films.
Video showing the continuous roll-to-roll collection process, in which the CNT film is continuously drawn from the outlet of the furnace (shown at normal speed).

Supplementary Movie 2 | Degradation of myoelectric cursor control with wet electrodes over 48 h.
Video comparing cursor trajectories obtained with wet electrodes at 0 h and 48 h using the initial decoder (D0), illustrating degradation in control performance over time without decoder retraining.

Supplementary Movie 3 | Long-term stability of myoelectric cursor control with CNT electrodes.
Video comparing cursor trajectories obtained with CNT electrodes at 0 h, 48 h, and 312 h using the initial decoder (D0), demonstrating sustained control performance over extended wear without decoder retraining.
