# Supplementary material for: Multi-day recalibration-free electrophysiology interface based on carbon nanotube thin film dry electrodes
Source: Nat Commun. 2026 Oct 13;17:11583. doi: 10.1038/s41467-026-78169-6 (PMC13751614; doi:10.1038/s41467-026-78169-6)
Supplement: Supplementary file 3 [file 41467_2026_78169_MOESM6_ESM.pdf]

nature portfolio

Corresponding author(s): Ana C. Arias

Last updated by author(s): Jul 5, 2026

Reporting Summary

Nature Portfolio wishes to improve the reproducibility of the work that we publish. This form provides structure for consistency and transparency in reporting. For further information on Nature Portfolio policies, see our Editorial Policies and the Editorial Policy Checklist.

Statistics

For all statistical analyses, confirm that the following items are present in the figure legend, table legend, main text, or Methods section.

n/a	Confirmed	
☐	☒	The exact sample size (*n*) for each experimental group/condition, given as a discrete number and unit of measurement
☐	☒	A statement on whether measurements were taken from distinct samples or whether the same sample was measured repeatedly
☐	☒	The statistical test(s) used AND whether they are one- or two-sided<br>*Only common tests should be described solely by name; describe more complex techniques in the Methods section.*
☒	☐	A description of all covariates tested
☐	☒	A description of any assumptions or corrections, such as tests of normality and adjustment for multiple comparisons
☐	☒	A full description of the statistical parameters including central tendency (e.g. means) or other basic estimates (e.g. regression coefficient) AND variation (e.g. standard deviation) or associated estimates of uncertainty (e.g. confidence intervals)
☐	☒	For null hypothesis testing, the test statistic (e.g. *F*, *t*, *r*) with confidence intervals, effect sizes, degrees of freedom and *P* value noted<br>*Give P values as exact values whenever suitable.*
☒	☐	For Bayesian analysis, information on the choice of priors and Markov chain Monte Carlo settings
☒	☐	For hierarchical and complex designs, identification of the appropriate level for tests and full reporting of outcomes
☒	☐	Estimates of effect sizes (e.g. Cohen's *d*, Pearson's *r*), indicating how they were calculated

Our web collection on statistics for biologists contains articles on many of the points above.

Software and code

Policy information about availability of computer code

Data collection	Data collection for myoelectric experiments used RZ2 BioAmp Processor with SIM Subject Interface Module.
Data analysis	Data analysis and figures were made in Matlab (2020b), Python (3.15.5), Origin (2023b) Custom code is available upon request.

For manuscripts utilizing custom algorithms or software that are central to the research but not yet described in published literature, software must be made available to editors and reviewers. We strongly encourage code deposition in a community repository (e.g. GitHub). See the Nature Portfolio guidelines for submitting code & software for further information.

Data

Policy information about availability of data

All manuscripts must include a data availability statement. This statement should provide the following information, where applicable:

- Accession codes, unique identifiers, or web links for publicly available datasets
- A description of any restrictions on data availability
- For clinical datasets or third party data, please ensure that the statement adheres to our policy

All data supporting the findings of this study are available within the article and its supplementary files. Any additional requests for information can be directed to, and will be fulfilled by Ana C. Arias.

Research involving human participants, their data, or biological material

Policy information about studies with human participants or human data. See also policy information about sex, gender (identity/presentation), and sexual orientation and race, ethnicity and racism.

Reporting on sex and gender	Sex and gender were not considered as experimental variables in the study design because the human-participant experiments were conducted as pilot study for noninvasive device feasibility and benchmarking studies of device performance. Participants were adult volunteers who provided informed consent. Sex and/or gender information was self-reported by participants where available. The study was not designed or powered to assess sex- or gender-dependent differences. Therefore, sex- or gender-disaggregated source data and sex-/gender-based analyses are not provided.
Reporting on race, ethnicity, or other socially relevant groupings	Race, ethnicity, and other socially relevant grouping variables were not collected, used for participant classification, or included in the analyses.
Population characteristics	Human-participant studies were performed in healthy adult volunteers between 18 and 40 for noninvasive skin-mounted electrophysiological recording and wearability testing. The cohort was used for device feasibility and within-subject/device-comparison studies rather than population-level inference and was not recruited as a clinical population.
Recruitment	Participants were recruited by convenience sampling from the University of California, Berkeley research community. This recruitment strategy may introduce self-selection bias and limits population-level generalizability due to participants' age and health conditions. These limitations are not expected to alter the main conclusions because the primary outcomes were engineering performance metrics and within-subject/device comparisons rather than estimates of population prevalence or clinical efficacy.
Ethics oversight	University of California, Berkeley Institutional Review Board

Note that full information on the approval of the study protocol must also be provided in the manuscript.

Field-specific reporting

Please select the one below that is the best fit for your research. If you are not sure, read the appropriate sections before making your selection.

☒ Life sciences ☐ Behavioural & social sciences ☐ Ecological, evolutionary & environmental sciences

For a reference copy of the document with all sections, see nature.com/documents/nr-reporting-summary-flat.pdf

Life sciences study design

All studies must disclose on these points even when the disclosure is negative.

Sample size	No statistical methods were used to predetermine sample size. Sample sizes were chosen based on the feasibility of long-duration wearable-device testing, the amount of material available for repeated fabrication and characterization, and sample sizes commonly used in early-stage bioelectronic device validation studies. The study was designed to evaluate engineering feasibility, device stability, and within-subject/device comparisons rather than to estimate population-level clinical effects. All reported sample sizes are provided in the relevant figure captions and Methods sections.
Data exclusions	Data collected during early device validation and study protocol development were not included in the final analyses. These preliminary recordings were used to establish device handling, electrode placement, recording parameters, task design, and analysis workflows before the finalized experimental protocol was implemented. Final analyses were performed only on data collected using the finalized device configuration and study procedures.
Replication	Experimental findings were verified through repeated measurements across independently prepared CNT films, multiple devices, and repeated recording sessions. The number of repetition is specified in the relevant main text, figure captions and Methods sections.
Randomization	Randomization is not applicable to the human wearable-device experiments.
Blinding	Investigators were not blinded to group allocation during data collection or analysis. Blinding was not practical for the wearable-device experiments because CNT, wet, and commercial dry electrodes were visually and procedurally distinguishable during placement and recording. The main outcomes were quantitative measurements, including impedance, signal-to-noise ratio, motion artifacts, electrophysiological signal features, and task-performance metrics, analyzed using the same procedures across conditions.

Reporting for specific materials, systems and methods

We require information from authors about some types of materials, experimental systems and methods used in many studies. Here, indicate whether each material, system or method listed is relevant to your study. If you are not sure if a list item applies to your research, read the appropriate section before selecting a response.

Materials & experimental systems

n/a	Involved in the study
☐	☒ Antibodies
☐	☒ Eukaryotic cell lines
☒	☐ Palaeontology and archaeology
☐	☒ Animals and other organisms
☒	☐ Clinical data
☒	☐ Dual use research of concern
☒	☐ Plants

Methods

n/a	Involved in the study
☒	☐ ChIP-seq
☒	☐ Flow cytometry
☒	☐ MRI-based neuroimaging

Antibodies

Antibodies used

Anti-CD3 [SP7] (ab16669) and anti-CD68 (ab31630) purchased from Abcam were used to assess biocompatibility. Both antibodies were diluted 1:100 in the final solution for immunostaining.

Validation

Validation statements for the above antibodies can be found through the following links to the manufacturer's website.
1. https://www.abcam.com/en-us/products/primary-antibodies/cd3-epsilon-antibody-sp7-ab16669
2. https://www.abcam.com/en-us/products/primary-antibodies/cd68-antibody-ed1-ab31630

Eukaryotic cell lines

Policy information about cell lines and Sex and Gender in Research

Cell line source(s)

Normal adult human dermal fibroblasts (HDF) cells were obtained from Lonza

Authentication

Not performed; commercially sourced HDF cells

Mycoplasma contamination

Mycoplasma contamination was not observed in the cell cultures.

Commonly misidentified lines
(See ICLAC register)

N/A

Animals and other research organisms

Policy information about studies involving animals; ARRIVE guidelines recommended for reporting animal research, and Sex and Gender in Research

Laboratory animals

All animal experiments were conducted in accordance with institutional guidelines for the care and use of laboratory animals and were approved by the Institutional Animal Care and Use Committee (IACUC) of the California Institute of Technology (Caltech) under an approved animal protocol.C57BL/6 wild type male mice were obtained from Jackson Laboratory. Mice were housed under specific-pathogen-free conditions in the animal facilities at the California Institute of Technology. Animals were maintained on a 12-h light/12-h dark cycle at an ambient temperature of approximately 22 ± 2 °C and relative humidity of 40–60%. Mice were housed in standard laboratory cages with appropriate bedding and environmental enrichment and had ad libitum access to standard laboratory chow and water. Mice used for all experiments were approximately 8 weeks of age.

Wild animals

The study did not involve wild animals

Reporting on sex

Biological sex was not investigated as an experimental variable in the animal study. The in vivo experiment was designed to evaluate local tissue compatibility and inflammatory response to the device under a defined implantation/contact condition.

Field-collected samples

The study did not involve field-collected samples.

Ethics oversight

California Institute of Technology, Institutional Animal Care and Use Committee

Note that full information on the approval of the study protocol must also be provided in the manuscript.

Plants

Seed stocks	N/A
Novel plant genotypes	N/A
Authentication	N/A
