# Supplementary material for: Multi-day recalibration-free electrophysiology interface based on carbon nanotube thin film dry electrodes
Source: Nat Commun. 2026 Oct 13;17:11583. doi: 10.1038/s41467-026-78169-6 (PMC13751614; doi:10.1038/s41467-026-78169-6)
Supplement: Supplementary file 4 [file 41467_2026_78169_MOESM7_ESM.pdf]

nature portfolio

Peer Review File

Multi-day Recalibration-free Electrophysiology Interface Based on Carbon Nanotube Thin Film Dry Electrodes

Corresponding Author: Professor Ana Arias

This file contains all reviewer reports in order by version, followed by all author rebuttals in order by version.

Version 0:

Reviewer comments:

Reviewer #1

(Remarks to the Author)
Summary and overview:
The manuscript reports on skin electrodes for long-term monitoring without the need for frequent recalibration. The authors fabricate, characterize and apply carbon nanotube (CNT) thin film dry electrodes to investigate the stability of multi-day electromyogram recording and the necessity of machine-learning (ML) model retraining. They report that thanks to both low impedance and stable electrode-skin interface, no ML model retraining is needed, which represents an important advance for ambulatory ML-driven wearable systems. Overall, the study aims to contribute to unobtrusive electrophysiology interfaces for long-term monitoring, with a holistic paradigm; from materials to system integration and ML training in real-wear conditions and comparison with commercial technologies.
The manuscript contains interesting results and the experiments and analysis appear carefully conducted. The originality seems to stem primarily from the ML methodology applied to the myoelectric monitoring, rather than from the material system itself. The central conclusion that CNT electrodes reduce the need of retraining and offer comfortable electrodes use, is supported by the material properties (conformality, contact density, and sheet resistance) that result in low contact impedance, high signal-to-noise ratio and reduced motion artifacts. The materials properties have been thoroughly investigated in the study and explained thanks to data fitting and physical interpretation. With some clarifications and additional discussion, it could be suitable for publication.

Major comments:
1. Novelty. The key demonstration of this study is the multi-day ML inference stability in naturalistic conditions. While the authors provide two tables in Supplementary Information comparing the performance of dry electrodes based on nanomaterials and continuous conductive films, the degree of novelty relative to previous studies should be clarified more explicitly in the main article. In particular, it would be helpful to understand whether the main advance lies primarily in the machine-learning framework or whether there are additional novel aspects related to the materials fabrication or the physical interpretation.
In addition, several recent studies have reported dry electrodes capable of stable electrophysiological monitoring under long-term wear and/or sweat exposure that do not appear to be included in the benchmarking. Including and discussing these works would help place the present results in context. Some examples are Du et al. ACS Nano 18, 34971–34985 (2024), Harimurti et al. Mater. Today 74, 94–108 (2024), Ma et al. Chem. Eng. J. 490, 151118 (2024).

2. Sheet resistance protocol measurement. Regarding the optimization of electrode impedance, the authors explore the parameters governing the skin-contact impedance of ultrathin network-based electrodes and identify three distinct operational regimes (ultra-thin to thinner networks, impedance reduction; thicker/less conformal films, impedance increase; and thick films, impedance saturation). These trends are supported by fitting with well-described models provided in the Supplementary Information.
A key parameter in this analysis is the sheet resistance, which the authors report measuring using a four-point probe station. While this technique is generally reliable for continuous thin films, its accuracy can be more challenging for percolated nanomaterial networks, particularly for sparse networks such as those reported in this study (see Table S5). In such systems, probe spacing relative to the characteristic network length scales, as well as local heterogeneity in the network density, may lead to significant variability in the measured values. In addition, the contact pressure applied by the probes can locally modify the network morphology or junction resistance, potentially affecting the measurement. It would therefore be helpful if the authors could provide additional details on the sheet resistance measurements and comment on the reliability of the

method in this context. For example, did the authors compare the four-point probe measurements with alternative approaches, such as two-point measurements using side contacts combined with geometrical estimation? Additional clarification would strengthen the robustness of the reported impedance analysis.

3. CNT interconnection. The manuscript introduces the concept of as-deposited macroscopically entangled networks enabled by the specific fabrication technique (floating catalyst chemical vapor deposition). The authors suggest that this structure enhances the mechanical robustness of the CNT-based electrodes and limits the presence of free or respirable nanotube fragments. Moreover, as described in Supplementary Method S1, films of varying thickness were not synthesized directly but assembled layer-by-layer to ensure intrinsic materials properties.
However, it would be helpful if the authors could provide additional evidence or discussion supporting the claim that the single-layer networks are already well interconnected or entangled directly during synthesis, and that the multilayer constructs maintain good interconnection with low contact electrical resistance between the layers. For example, do the authors have experimental characterization (e.g., microscopy, mechanical tests, electrical testing) or literature references demonstrating the degree of interconnection within the as-deposited networks? Clarifying this point would be important, as the interpretation of the electrode robustness appears to rely on the assumption of a well-connected entangled network.

4. ML-related data reproducibility. In the sections on electrophysiological recordings and ML inference stability testing, the experiments appear to have been conducted on a single subject (as indicated in the Methods). Given that the multi-day decoder stability in myoelectric control represents a central claim of the study, it would be helpful if the authors could comment on the reproducibility of these results. For example, were additional trials performed (e.g., repeated experiments, different electrode sets, or other users), or can the authors provide further discussion or references supporting the robustness of this approach?

5. Sweat effect. The authors suggest that the porosity of the CNT-based electrodes contributes to their stability under conditions relevant to wearable applications, and they provide measurements of water vapor transmission rate to demonstrate breathability. The authors also mention that, thanks to their porous nature, CNT electrodes are resistant to sweat and body heat. However, since these electrodes are intended for long-term skin contact, it would be important to clarify how exposure to sweat may affect their stability and performance. While investigating the effects of sweat may not have been the primary objective of the study, additional discussion supported by experimental data or relevant literature on sweat exposure would strengthen the assessment of their suitability for long-term wearable use.

Minor comments:
1. The thickness and electrical-optical properties of the electrodes applied in the electrophysiology monitoring and the multi-day decoder test should be explicitly mentioned.

2. Units should be specified in Table S5. The average values of T and R could also be reported with a consistent number of significant digits.

3. Regarding the use of the term Boolean deposition model, please provide relevant references supporting this terminology and examples where similar approaches (of areal coverage and thickness statistics based on a binary system) have been used in percolative networks, to clarify the statement made in the Supplementary Information.

4. Photographs (e.g., in the Supplementary Information) of the applied skin electrodes and wiring in the electrophysiological recordings and multiday decoder stability test, particularly the thicker leads connected to the electrodes, would help clarify the overall experimental setup, even though the interconnection method is not the main focus of the study.

Reviewer #2

(Remarks to the Author)
Multi-day Recalibration-free Electrophysiology Interface Based on Carbon Nanotube Thin Film Dry Electrodes

Summary

This manuscript presents an innovative solution to the long standing challenges of instability, noise susceptibility, and signal drift in contemporary electrophysiological electrodes. These interfaces are essential for capturing a wide range of biosignals, including electrocardiography (ECG) for cardiac health, electromyography (EMG) for muscle activity, and electroencephalography (EEG) for monitoring brain states, which are increasingly integrated into high dimensional representations of user physiology. A particularly noteworthy aspect of the work is the use of micron thin dry electrodes based on carbon nanotube (CNT) thin films, a material platform that offers mechanical flexibility, conformal skin contact, and enhanced signal fidelity without the need for conductive gels.

The topic is modern and interdisciplinary, effectively integrating materials science with bioelectronic engineering. The long term, recalibration free signal stability demonstrated in the study is particularly impressive and highlights the potential of these CNT-based electrodes for broader use in practical bioelectronic systems.
The figures and diagrams accurately reflect the experimental workflow and outcomes, and the results are presented clearly and coherently. The experiments are well designed, and the data are carefully analyzed, which strengthens the credibility of the conclusions. The manuscript convincingly articulates the need for research in this direction and clearly emphasizes the advantages of the proposed approach.

Minor comments:

Line 115, Page 3: "…three coupled parameters that govern electrode-skin contact quality." - Please specify which parameters are being referred to.

Lines 141 - 160, Page 5: The manuscript does not clearly state which synthesis conditions were ultimately used to fabricate the CNT films employed in the electrophysiology experiments. Supplementary Tables S4 - S5 list representative conditions, and Fig. 2e - f identifies an optimal parameter window, but the exact growth parameters (e.g., gas flow rates, furnace temperature, draw speed) corresponding to the "optimized conditions" are not explicitly provided. For reproducibility, please include the precise synthesis parameters used for all CNT films. Which furnace temperature was optimal? Was the temperature held constant, or do variations in the 1000 - 1200 °C range not affect CNT quality?
Synthesis stability: It may be beneficial to move the synthesis stability assessment from the Supplementary Information into the main text. Demonstrating stability under the selected conditions would directly support the claim of scalability. It may also be useful to mention that sheet resistance drift was quantified and to briefly describe the issue of reactor clogging - how often does it occur, what causes it, and can it be prevented?

Lines 184 - 188, Page 5 - 6: How does the material perform during intensive patient movement, sweating, or elevated temperatures? Is this relevant for clinical or ambulatory applications?

Line 227, Page 7: The manuscript states that film thickness was controlled by tuning growth conditions, whereas Supplementary Method S1 clarifies that thickness was achieved exclusively through layer by layer stacking of identical films. This discrepancy should be corrected, and the main text should explicitly state that films of varying thickness were not synthesized directly. This is important for readers' understanding of the fabrication process.

Line 248, Page 8: Is it important to measure impedance at frequencies other than 10 Hz? What are the relevant frequency ranges for EEG, EMG, and ECG?

Line 269, Page 9: Could you provide quantitative data on CNT migration within tissues? Did the implantation tests include an analysis of material degradation?

Line 293, Page 10: Would it be valuable to extend the skin compatibility (irritation) test beyond 3 h, for example to 48 h? This would allow a more direct comparison with wet electrodes, which were tested for 48 h, and would better support the claim of "long term skin compatibility".

Line 304, Page 11: Would it be useful to include a control condition using other types of dry electrodes?

Line 342, Page 13: Please clarify why these electrode placement sites were chosen. Could the test be performed at other locations?

Line 346, Page 13: During the multi day stability tests, were the electrodes removed and reapplied (e.g., during showering)? If so, please provide data. Did electrode displacement occur, or were they kept in the exact same position throughout?

Line 357, Page 13: Was the multi day MI test performed on only one participant?
Sample size: The number of samples (n) for SEM, TEM, Raman, WVTR, and mechanical testing is not provided. Please include this information for reproducibility.
Impedance statistics: Please provide full statistical information for impedance measurements (n, SD, statistical tests). In Fig. 3h, the number of repetitions is missing.
Limitations: Please discuss potential limitations of the study, such as the absence of clinical testing and the lack of long term (>72 h) evaluations.

Verdict: Acceptance with minor revisions.

Reviewer #3

(Remarks to the Author)
Yunhan Wen et al. reported the preparation, characterization and application of carbon nanotube (CNT) thin film dry electrodes, and realized a recalibration-free myoelectric interface for long-term electrophysiological monitoring. The study content is detailed and the technical route is relatively complete. However, the manuscript still needs major revision in several aspects such as structural organization, data interpretation and comparative analysis. Specific comments are as follows:

1. The overall structure of the manuscript hinders readability. For instance, the Introduction is overly lengthy and should be condensed. The paragraph immediately following the "Results and Discussion" heading reads more like a summary of the entire study and could be integrated into the end of the Introduction (indeed, it partially overlaps with the final two paragraphs of the Introduction). If the authors prefer to retain this paragraph in the Results section, the corresponding conclusions must be supported by specific data. For example, when reading the sentences, "The biocompatibility of the CNT film is validated through in vitro cytotoxicity assays, skin patch test, and subcutaneous implantation test on mice models. Electrode performance in electrophysiological recordings of ECG, EEG, and EMG signals, demonstrated improved signal fidelity

compared to conventional wet electrodes," no citations to corresponding figures or data are provided. It is strongly recommended that the authors add detailed citations to figures and data throughout the main text to help readers quickly locate supporting evidence, particularly for the "Electrode Impedance and Optimization" section and Figure 3.

2. The authors compared the CNT electrodes with PEDOT, metal nanowire, and Ag/AgCl wet electrodes, but neglected the comparison with many emerging flexible electrodes in recent years, such as conductive hydrogel electrodes, liquid metal flexible electrodes, and flexible electrodes fabricated by semiconductor micro/nano processes. It is suggested to supplement a multi-dimensional comparison with these electrodes in terms of impedance, signal-to-noise ratio, wearing duration, biocompatibility, etc., to clarify the core competitive advantages and shortcomings of the CNT electrodes.

3. Related to the above point, the authors mention in Supplementary Tables S2-S3 that the tensile performance of some metal electrodes can reach 500%. However, the comparison in Figure 2k only includes metal nanowire electrodes. This selective comparison, which appears to highlight the advantages of CNT thin films, should be revised to include a broader and more representative range of devices.

4. All experiments were conducted under normal, static conditions, with the only motor test being repetitive wrist movement. The performance stability of the electrodes under more challenging scenarios (e.g., during strenuous exercise or heavy sweating) was not investigated. It is recommended to supplement the study with performance tests under such conditions to better define the application boundaries of the electrodes.

5. While the authors compared the skin irritation of different electrodes after continuous wear in the biocompatibility assessment, they did not investigate the long-term electrical stability of the CNT electrodes after prolonged use. It is crucial to characterize the electrical properties after long-term wearing experiments, analyze potential failure mechanisms, and verify whether the claimed "low skin–electrode impedance and high signal fidelity" can be maintained over extended periods.

6. The biocompatibility assessment after subcutaneous implantation, which only used CD3 and CD68 markers, is insufficient. Given that CNTs have the potential to be phagocytosed by macrophages and retained in organs like the liver and spleen, a more comprehensive evaluation is necessary. It is suggested to include the detection of inflammatory factors such as TNF-α, IL-1β, and IL-6, quantify the CNT content in various organs, and compare the structural and morphological changes of the CNT thin films before and after implantation.

7. Several issues exist in the figures and statistical analysis:
(1) Fig. 1b: The specific type of skin replica used is not specified, and the SEM image lacks detailed information. It is recommended to provide SEM images of the skin replica with half the area covered by the CNT thin film and the other half uncovered, including magnified images of the edge between the two areas. This would more clearly demonstrate the conformability of the CNT film to the skin.
(2) Fig. 2e: Please add units to the color gradient scale.
(3) Fig. 3: The text describes Figure 3b before Figure 3a, which is inconsistent with the figure's sequence. Please reorder the text or the figure panels accordingly.
(4) Fig. 3d: The SEM image quality is poor. The specific type of skin surface used for the SEM test is not indicated, and the image fails to clearly illustrate why the CNT film with 30% transmittance cannot conform well to the skin's micro-valleys.
(5) Statistics: The statistical analysis methods and sample sizes (n values) are not clearly stated for some experimental data, which diminishes the rigor of the results.

Reviewer #4

(Remarks to the Author)
This manuscript presents a comprehensive engineering effort to develop carbon nanotube (CNT) thin-film-based dry electrodes for long-term electrophysiological monitoring.The work is technically sound, the experimental data are robust, and the demonstration of a 48-hour, recalibration-free myoelectric interface is a notable and practically relevant result. The systematic approach from scalable material synthesis and optimization of the electrode-skin interface to final system-level validation is a strength. However, as the editor and reviewer have highlighted, the degree of conceptual novelty requires careful scrutiny to meet the high bar of Nature Communications.While the individual components (CNT films, dry electrodes, myoelectric interfaces) are not new,the integration and the specific demonstration of long-term signal stability for ML inference without retraining could constitute an advance.Therefore, my major critiques center on clarifying and strengthening this claim of novelty, ensuring robust comparisons, and bolstering the mechanistic insights.
Major comments:
1.The manuscript demonstrates continuous electrode wear for three days and interprets this as evidence of long-term stability. However, several previously reported dry electrode materials, such as PEDOT:PSS and silver nanowire networks, have also demonstrated stable electrophysiological recordings over multiple days. Therefore, it is unclear whether a three-day test is sufficient to support the claim of “long-term stability.”Could the authors clarify the expected performance limits of the proposed electrode? If the system requires retraining after only three days of operation, the practical advantage over existing dry electrodes may be limited. Additional validation over longer time scales (for example, several weeks)would strengthen the claim of long-term stability.
2.The authors should provide a more nuanced discussion in the Introduction and/or Discussion that directly compares their work with the state-of-the-art in "stable" dry electrodes. For instance, while PEDOT and other nanostructured dry electrodes are mentioned, what is the current best record for stable, recalibration-free operation in similar myoelectric or other

electrophysiology tasks? Although table S2 and S3 provide a comparison, it is not clear enough. A dedicated table or expanded narrative comparing key metrics (duration, performance decay, required retraining frequency) with prior art would solidify the manuscript's contribution, especially the current best record in this field. Without this, the work risks being perceived as an incremental improvement in material performance rather than a paradigm shift in interface stability.
3.The manuscript reports stable electrical performance under 30% strain. Is 30% the maximum tensile strain that the CNT film can sustain? During the stretching process, the relative resistance change appears to decrease. Could this behavior arise from CNT fracture followed by re-alignment or densification of the network, which may create additional conductive pathways? If nanotube fracture does occur, it raises a potential concern regarding the release of nanoscale fragments. In the absence of a protective encapsulation layer, could residual CNT fragments remain on the skin surface after repeated deformation? If so, this may have implications for the long-term biocompatibility and safety of the electrode.
4.The manuscript reports mechanical stability under 30% strain, but it is unclear which film thickness (or optical transmittance) corresponds to this performance. Could the authors specify the transmittance (or thickness) of the CNT film that achieves this level of stretchability? Furthermore, when the film thickness is increased to reduce contact resistance, the mechanical properties (e.g., flexibility, stretchability, and conformability) may change accordingly.Have the authors systematically evaluated how film thickness affects these mechanical properties? Such variations may influence the electrode's ability to maintain conformal contact with the skin and may ultimately affect its long-term stability during practical use.
5.Human skin is generally considered a low surface-energy interface, which may make the direct and damage-free transfer of ultrathin CNT films challenging. Could the authors clarify the specific method used to transfer the CNT electrodes onto the skin? If a transfer medium such as temporary tattoo paper is used, it would be helpful to understand whether this intermediate layer affects key electrode properties, such as breathability, mechanical compliance, or electrical contact with the skin.
6.As for the depth of multi-day demonstration,the performance decay of the wet electrodes is attributed to gel dehydration/signal drift. Could the authors provide direct, simultaneous measurements of the impedance or a simple signal quality metric (e.g., SNR, baseline noise) for both CNT and wet electrodes over the same long-term period, such as several weeks? This would directly correlate the observed performance stability/degradation with the hypothesized interfacial stability/drift. Figure 3h shows impedance variation under disturbance, but not long-term drift.
7.The manuscript attributes stability to the material properties (porosity and Van der Waals adhesion). While plausible, the analysis is somewhat phenomenological.A more mechanistic investigation is needed. For example:The role of Van der Waals adhesion in maintaining conformal contact is emphasized. Are there quantitative data on adhesion energy or measurements of contact conformity (e.g., via optical coherence tomography) after several days of wearing, including during/safter activities like hand washing or sweating?
8.The claim that CNTs are electrochemically stable in sweat/body heat is not supported by new data. Please provide potentiostatic electrochemical impedance spectroscopy (EIS) data for the CNT film in an artificial sweat solution at 37°C over serveral days, comparing it to a benchmark material like PEDOT:PSS, and some other commonly used nanomaterials.
9.The description of decoders D1-D3 is confusing. It states they were trained "for comparison" (Line 347). The conclusion that retraining yields no improvement for CNT electrodes is vital. This should be shown directly in the main figure, perhaps by adding a panel comparing the performance of D0 vs. D1/D2/D3 at the 6h, 24h, 48h time points for CNT and wet electrodes side-by-side.
10.As for the task complexity,the center-out task is standard. To better showcase the stability's importance, could the authors include a more dexterous task (e.g, a virtual keyboard typing task requiring discrete selections) at the beginning and end of the 48-hour period using the sameinitial decoder (D0)? This would powerfully demonstrate the real-world utility of a "set-and-forget" interface.
Minor Comments:
1.Abstract/Title: The title and abstract emphasize "Multi-day Recalibration-free." The data only supports 48 hours (2 days). "The authors should be precise in the text (e.g., "two days").
2.Fig.2k:The radar plot is useful. Please ensure the axes are clearly defined in the caption or figure. What is the source of the data for PEDOT and metal nanowire benchmarks? Cite appropriately.

Version 1:

Reviewer comments:

Reviewer #1

(Remarks to the Author)
The authors responded to every raised question in a rigorous manner, performing additional experiments and clarifying in the text the remaining limitations and prospects. In my opinion, the manuscript is now complete and suitable for publication.

Reviewer #2

(Remarks to the Author)
The manuscript has been significantly strengthened, particularly through the addition of extended 14-day wear tests, physiological stress evaluations (e.g., sweating, shower conditions), and the clarification of both synthesis parameters and biocompatibility boundaries. Furthermore, all Supplementary Information has been appropriately integrated and cited within the main text.

Reviewer #3

(Remarks to the Author)
The authors have addressed the comments raised by reviewers. Revisions have been carried out to resolve core issues, including the description of material fabrication parameters and comparative analysis against literature, as well as recordings of multiple electrophysiological signals. New experimental data covering scenarios of sweating, physical exercise, and hot shower exposure have been supplemented, and critical experimental information such as sample sizes and statistical parameters has been completed.
As for the limitations that remain unaddressed in the current work, including the absence of real-time myoelectric decoding data captured synchronously during strenuous sweating exercise, failure to record signals immediately after workouts, and the limitation of a single human participant in trials, the authors have outlined these drawbacks within the Limitations section of the manuscript. It is recommended that the authors add analytical discussion on the potential causes of these deficiencies, together with targeted key optimization strategies and future research directions. Overall, the manuscript has achieved substantial improvements in experimental integrity and the rigor of its supporting arguments.

Reviewer #4

(Remarks to the Author)
The authors have adequately addressed the previous concerns and have improved the overall quality of the manuscript. Therefore, this reviewer recommend it for publication.

Open Access This Peer Review File is licensed under a Creative Commons Attribution 4.0 International License, which permits use, sharing, adaptation, distribution and reproduction in any medium or format, as long as you give appropriate credit to the original author(s) and the source, provide a link to the Creative Commons license, and indicate if changes were made.
In cases where reviewers are anonymous, credit should be given to 'Anonymous Referee' and the source.
The images or other third party material in this Peer Review File are included in the article's Creative Commons license, unless indicated otherwise in a credit line to the material. If material is not included in the article's Creative Commons license and your intended use is not permitted by statutory regulation or exceeds the permitted use, you will need to obtain permission directly from the copyright holder.
To view a copy of this license, visit https://creativecommons.org/licenses/by/4.0/

General Acknowledgements to the Reviewers

Before addressing the individual comments, we would like to collectively thank the reviewers for dedicating their time and expertise to evaluating our manuscript. We deeply appreciate the rigorous review process and the highly constructive feedback provided. Your detailed corrections and insightful suggestions have been invaluable, and we genuinely believe that incorporating your recommendations has significantly improved the clarity, depth, and overall quality of this work. **Please find our detailed, point-by-point responses below.**

Reviewer #1 (Remarks to the Author):

Summary and overview:

The manuscript reports on skin electrodes for long-term monitoring without the need for frequent recalibration. The authors fabricate, characterize and apply carbon nanotube (CNT) thin film dry electrodes to investigate the stability of multi-day electromyogram recording and the necessity of machine-learning (ML) model retraining. They report that thanks to both low impedance and stable electrode-skin interface, no ML model retraining is needed, which represents an important advance for ambulatory ML-driven wearable systems. Overall, the study aims to contribute to unobtrusive electrophysiology interfaces for long-term monitoring, with a holistic paradigm; from materials to system integration and ML training in real-wear conditions and comparison with commercial technologies.

The manuscript contains interesting results and the experiments and analysis appear carefully conducted. The originality seems to stem primarily from the ML methodology applied to the myoelectric monitoring, rather than from the material system itself. The central conclusion that CNT electrodes reduce the need of retraining and offer comfortable electrodes use, is supported by the material properties (conformality, contact density, and sheet resistance) that result in low contact impedance, high signal-to-noise ratio and reduced motion artifacts. The materials properties have been thoroughly investigated in the study and explained thanks to data fitting and physical interpretation. With some clarifications and additional discussion, it could be suitable for publication.

Major comments:

1. Novelty. The key demonstration of this study is the multi-day ML inference stability in naturalistic conditions. While the authors provide two tables in Supplementary Information comparing the performance of dry electrodes based on nanomaterials and continuous conductive films, the degree of novelty relative to previous studies should be clarified more explicitly in the main article. In particular, it would be helpful to understand whether the main advance lies primarily in the machine-learning framework or whether there are additional novel aspects related to the materials fabrication or the physical interpretation.

In addition, several recent studies have reported dry electrodes capable of stable electrophysiological monitoring under long-term wear and/or sweat exposure that do not appear to be included in the benchmarking. Including and discussing these works would help place the present results in context. Some examples are Du et al. ACS Nano 18, 34971–34985 (2024), Harimurti et al. Mater. Today 74, 94–108 (2024), Ma et al. Chem. Eng. J. 490, 151118 (2024).

Author Response: In the revised manuscript, we have clarified that the main advance is not a new ML algorithm alone, nor simply the use of CNTs as dry electrodes. Rather, the contribution lies on a system that integrates: (i) a roll-to-roll scalable gas-phase-synthesis method of CNT network that is ultrathin, intrinsically percolated, and low sheet resistance; (ii) a physics-based electrode–skin impedance optimization framework; and (iii) a system-level validation of long-term interface stability using ML-relevant task metrics.

To elaborate the distinct contributions of this work, the CNT films used here differ from many solution-processed CNT or FCCVD CNT because our modified FCCVD process produces a binder-free, continuous, and free-standing CNT aerogel network that is already interconnected during synthesis. This avoids surfactant or ultrasound techniques for dispersion and helps lower the junction resistance. We have also introduced peripheral gas flow into the reactor to achieve direct synthesis of CNT aerogel (rather than aerosol), which is key to continuous roll-to-roll manufacturing of free-standing CNT films. These synthesis optimizations allow the electrodes to combine low sheet resistance, porosity, conformability, and scalable manufacturing.

We also established a theoretical framework to study the roles of nanoscale contact density, macroscopic conformability, and sheet resistance in the electrode–skin contact impedance. By experimentally and theoretically identifying distinct impedance regimes, the study provides a design map for porous thin-film dry electrodes.

For the long-term stability characterization, a key distinction of this work is that we evaluate electrode stability using task-level metrics that are more representative of real-world ML-driven interfaces and more stringent than conventional electrophysiological benchmarks. Prior studies commonly define long-term stability by the continued ability to record ExG signals, maintain low impedance or SNR, or preserve visually similar waveforms. While important, these metrics do not necessarily ensure stable downstream performance in multichannel ML systems. In high-channel-count systems, each channel must remain stable not only individually, but also relative to each other. Small channel-specific changes in electrode–skin impedance, signal amplitude, baseline noise, or motion artifacts can shift the multichannel feature distribution and substantially degrade task-level performance, even when individual waveforms remain distinguishable. A recent HDEMG study[1] similarly showed that despite modest average impedance changes and stable SNR, statistically significant electrode-specific impedance variability was observed. Although these changes did not significantly affect the overall SNR, they decreased the accuracy of gesture recognition for classifiers that rely on consistent patterns of sensed myoelectric activity over time. Recalibration after approximately 1 h restored much of the lost accuracy. This supports our use of ML task performance as a more stringent metric of electrode–interface stability than impedance or SNR alone. In addition, the probability of system failure increases significantly with the number of electrodes. Our myoelectric interface has 10 unipolar channels (5 bipolar pairs) targeting five distinct muscle groups. Achieving stable task performance across this multi-channel array for 14 days without retraining is a significantly higher bar than single-channel SNR stability.

This distinction in ML-level stability metrics is especially important for continuous myoelectric decoding. Although some studies have demonstrated multi-day gesture classification, this task maps EMG activity to a finite set of discrete labels and can therefore tolerate some signal drift, as long as the class boundaries remain separable. On the other hand, continuous decoding directly maps EMG features to real-time cursor kinematics such as direction and velocity. As a regression task performed in closed loop, it is more sensitive to small changes in signal amplitude, timing, and inter-channel relationships. Users also adapt their muscle activation in real time based on decoder output. Therefore, electrode drift can disrupt not only the input feature distribution, but also the user–decoder control strategy established during training. In this study, the Kalman filter decoder was trained using closed-loop decoder adaptation, making decoder stability without recalibration a particularly stringent benchmark. We fixed the decoder after initial training, so that later performance reflected electrode stability rather than ongoing algorithmic adaptation. Using a light-weight, train-once decoder also better matches practical wearable use, where

computational complexity adds constraints on processing and power. The ability of the CNT electrodes to preserve decoder performance over 14 days without retraining therefore demonstrates a level of long-term stability beyond conventional signal-quality metrics or discrete classification accuracy.

We have revised the Introduction to clarify the novelty and expanded benchmarking to include additional more state-of-the-art works (including Du et al., Harimurti et al., and Ma et al.) and other emerging techniques (such as liquid metal, hydrogel, and micromachined electrodes) in Supplementary Tables S2-S4.

2. Sheet resistance protocol measurement. Regarding the optimization of electrode impedance, the authors explore the parameters governing the skin-contact impedance of ultrathin network-based electrodes and identify three distinct operational regimes (ultra-thin to thinner networks, impedance reduction; thicker/less conformal films, impedance increase; and thick films, impedance saturation). These trends are supported by fitting with well-described models provided in the Supplementary Information.

A key parameter in this analysis is the sheet resistance, which the authors report measuring using a four-point probe station. While this technique is generally reliable for continuous thin films, its accuracy can be more challenging for percolated nanomaterial networks, particularly for sparse networks such as those reported in this study (see Table S5). In such systems, probe spacing relative to the characteristic network length scales, as well as local heterogeneity in the network density, may lead to significant variability in the measured values. In addition, the contact pressure applied by the probes can locally modify the network morphology or junction resistance, potentially affecting the measurement. It would therefore be helpful if the authors could provide additional details on the sheet resistance measurements and comment on the reliability of the method in this context. For example, did the authors compare the four-point probe measurements with alternative approaches, such as two-point measurements using side contacts combined with geometrical estimation? Additional clarification would strengthen the robustness of the reported impedance analysis.

Author Response: We thank the reviewer for this insightful comment regarding the reliability of sheet resistance measurements in sparse percolated CNT networks.

To further assess the robustness of the reported sheet resistance values, we compared measurements obtained using three independent methods: (i) a commercial Jandel four-point

probe system with 1.0 mm probe spacing, which was used for the primary measurements reported in the manuscript; (ii) a custom-built four-point probe system with a larger probe spacing of 2.54 mm; and (iii) a contactless eddy-current-based sheet resistance measurement system (Delcom Instruments).

The HRI custom-built 2.54 mm spaced probe was used to evaluate whether the measured resistance was sensitive to local network heterogeneity or probe spacing relative to the characteristic length scales of the CNT network. The Delcom contactless eddy-current measurements further confirmed whether contact pressure applied by the probes locally modified the network morphology or junction resistance, potentially affecting the measurement.

As shown below, the results from those two systems were plotted vs our standard four-point probe system by Jandel. We found roughly linear relationships between this system, with a correlating slope of 1.23 ± 0.06 for the contactless system and 1.19 ± 0.07 for the HRI custom-built 2.54 mm spaced probes. These results provide two independent validations of the reported sheet resistance values. The agreement between the two four-point probe systems with substantially different probe spacings indicates that the measurements are representative of the film as a whole rather than localized artifacts arising from network heterogeneity. In addition, the correlation between contact-based and contactless measurements suggests that probe-induced compression of the CNT network has a minimal effect on the measured resistance.

In addition, sheet resistance measurements were collected at multiple locations across each film and across independently synthesized samples. Consistent with typical thin-film measurements, greater variation was only observed near the film edges, whereas measurements across the central region of the film remained highly uniform. This observation is observed in all four point probe measurements.

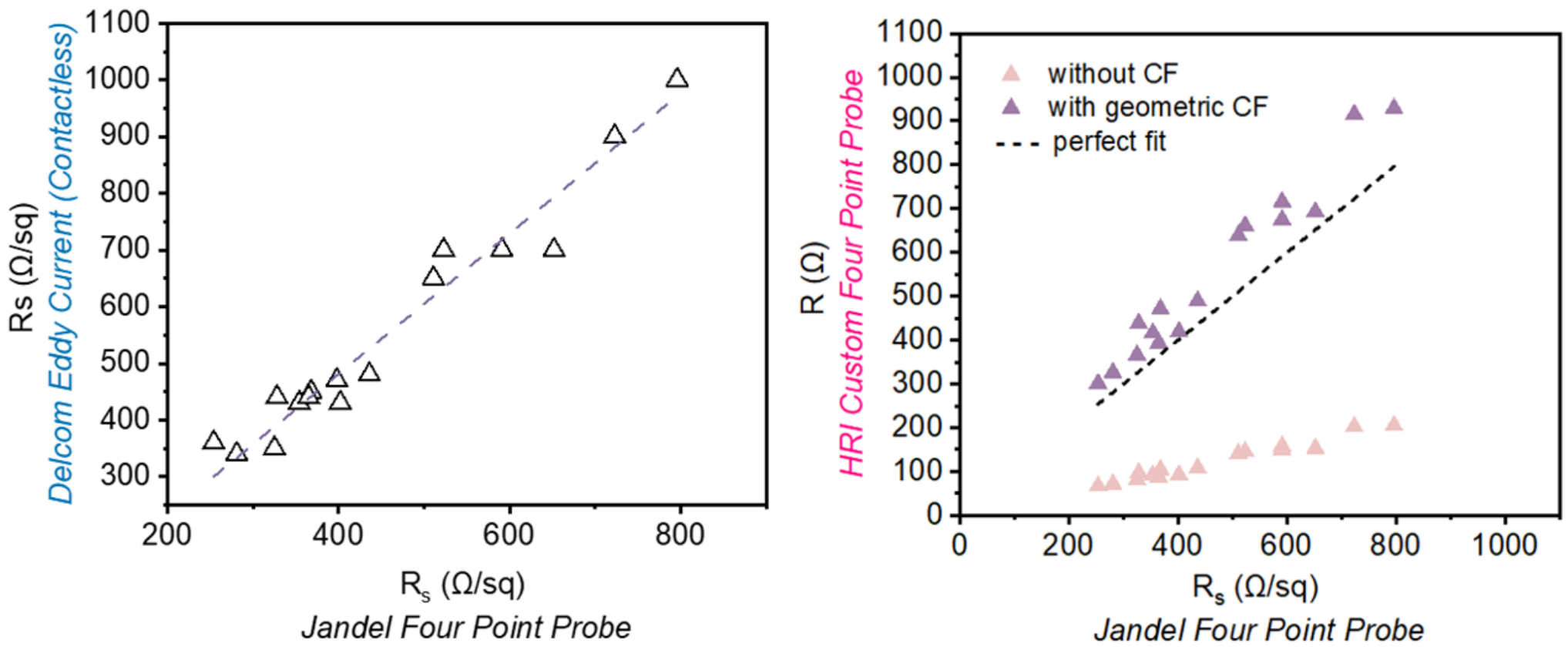

For the HRI custom-built 2.54 mm spaced four point probes, a geometric correction factor is necessary to compare custom probes to the commercial four-point probes. We used the equation,

$$R_s = CF * \frac{\pi}{\ln 2} \frac{\Delta V}{i}$$

and found the additional correction factor to be 0.93.

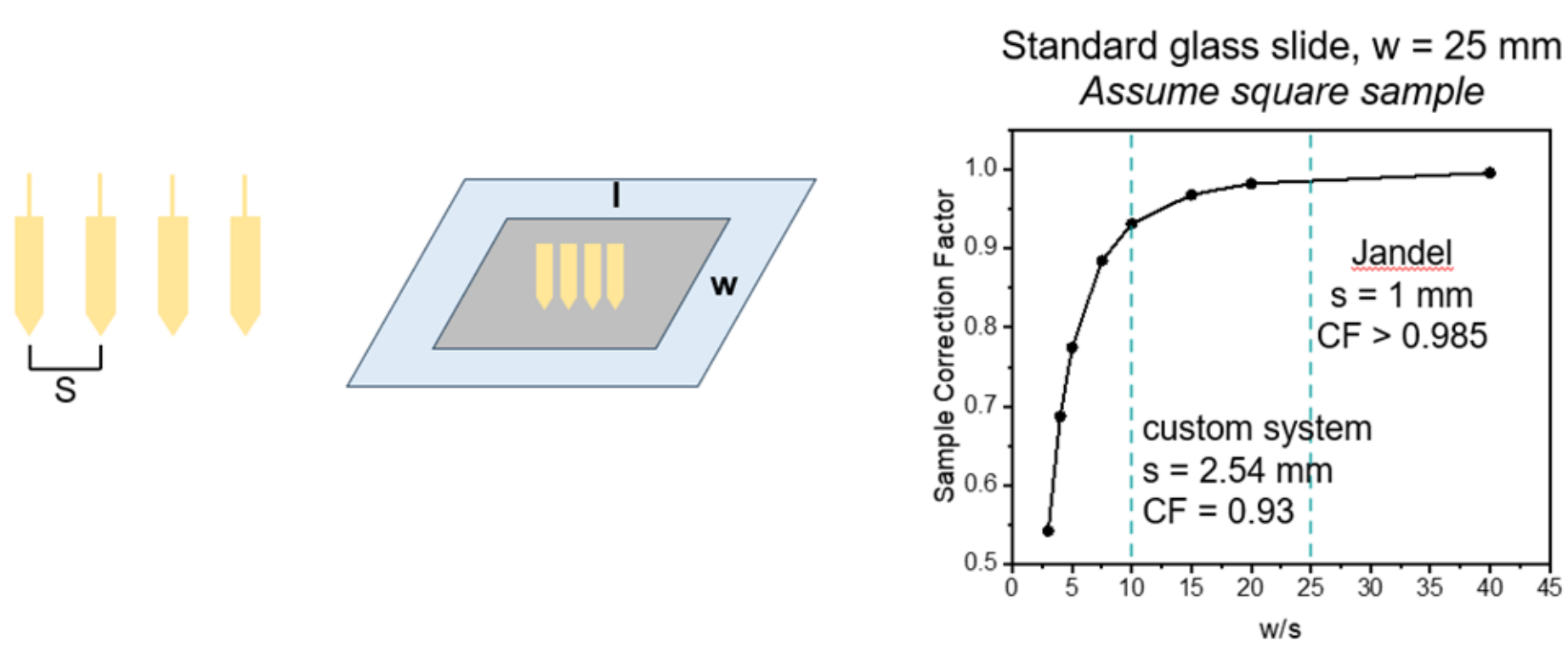

3. CNT interconnection. The manuscript introduces the concept of as-deposited macroscopically entangled networks enabled by the specific fabrication technique (floating catalyst chemical vapor deposition). The authors suggest that this structure enhances the mechanical robustness of the CNT-based electrodes and limits the presence of free or respirable nanotube fragments. Moreover, as described in Supplementary Method S1, films of varying thickness were not synthesized directly but assembled layer-by-layer to ensure intrinsic materials properties.

However, it would be helpful if the authors could provide additional evidence or discussion supporting the claim that the single-layer networks are already well interconnected or entangled directly during synthesis, and that the multilayer constructs maintain good interconnection with

low contact electrical resistance between the layers. For example, do the authors have experimental characterization (e.g., microscopy, mechanical tests, electrical testing) or literature references demonstrating the degree of interconnection within the as-deposited networks? Clarifying this point would be important, as the interpretation of the electrode robustness appears to rely on the assumption of a well-connected entangled network.

Author Response: We agree that clarifying CNT interconnection both within individual layers and across stacked layers is important for supporting our layer-by-layer assembly approach and our interpretation of FCCVD CNT films as directly synthesized, aerogel-like interconnected networks.

First, the interconnected nature of the as-deposited CNT films is what enables our roll-to-roll collection process. In our FCCVD process, CNT films are collected as an aerogel-like, entangled network rather than as individually deposited nanotubes. As the CNT aerogel exits the reactor, it retains a continuous, self-supporting tubular structure that can be mechanically guided and continuously drawn by a motorized roll-to-roll system directly onto the receiving substrate. This continuous collection process would not be possible if the CNTs were present as disconnected aerosol particles or as a collection of non-interconnected nanotubes. We have included a video (Movie S1) to further illustrate the collection process.

SEM and TEM characterization in Fig. 2c also shows a continuous, percolated CNT morphology, supporting the presence of an interconnected network within each deposited layer. Below, we have also added SEM images of a single CNT layer before and after densification by Ethanol. These images show that the entangled network morphology is present before densification and preserved after densification, further supporting that each deposited layer consists of a mechanically continuous and electrically percolated CNT network.

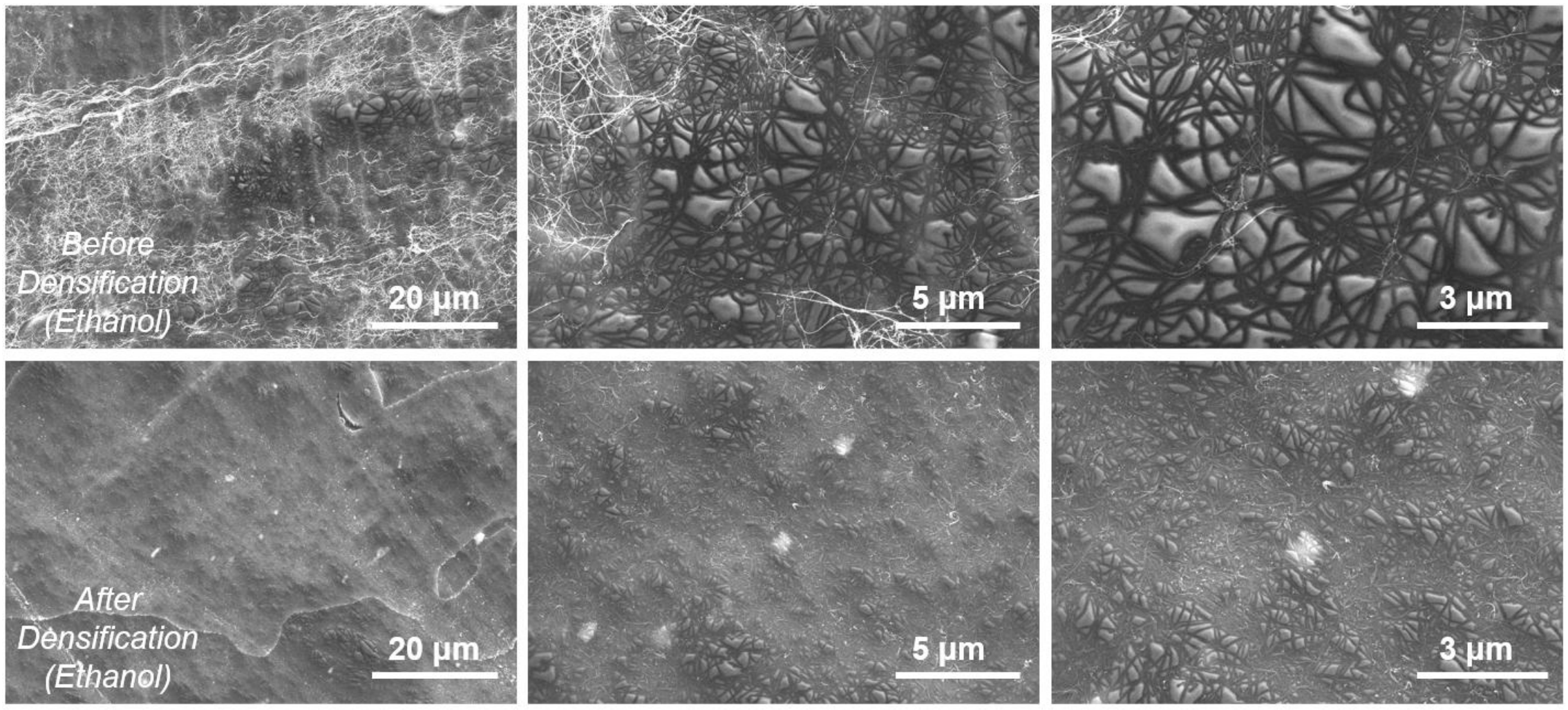

Supplementary Figure S6 | SEM images of entangled CNT network morphology of a single deposited layer. The as-deposited CNT film already exhibits an entangled, interconnected network morphology before densification. After ethanol densification, the film becomes more compact while retaining the continuous CNT network structure. The preserved network morphology supports that each deposited CNT layer forms a mechanically continuous and electrically percolated CNT film rather than isolated CNT aggregates. Scale bars: 20 µm, 5 µm, and 3 µm.

Second, the electrical behavior of the multilayer films supports effective interlayer coupling. As shown in the semi-log plot of sheet resistance vs. layer number below, the resistance decrease from 1-3 layers is non-linear; this would suggest new CNT junctions and bridging pathways, not simply disconnected parallel sheets. The plot becomes linear from 5-8 layers, suggesting the network is saturated. This is consistent with our SEM images of the films; beyond 3 layers, the structure appears more as a continuous CNT film than a sparse percolative network. As we continue to add layers, we can then consider new junctions to contribute less to the overall conductivity, and the behavior to trend towards bulk properties of a CNT film. Based on the behavior from 1-3 layers, we can conclude that the layers are electrically coupled and contribute to a common conductive network.

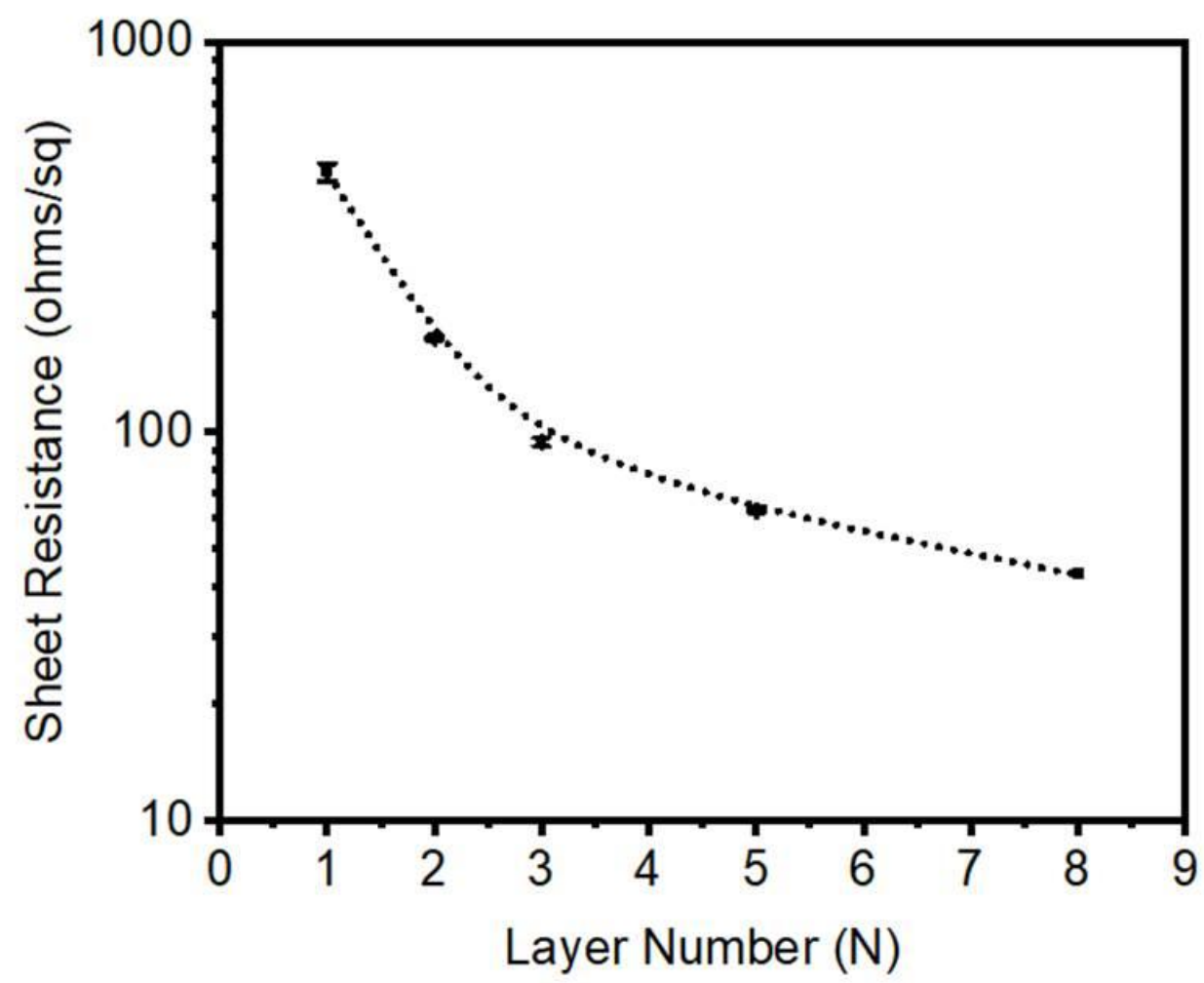

Supplementary Figure S11 | h, Sheet resistance as a function of layer number, showing consistency between experimental and theoretical values. The results confirm that stacking identical CNT layers yields a predictable exponential reduction in sheet resistance.

Third, this interpretation is consistent with the observed impedance behavior of the electrodes. Increasing the number of CNT layers initially reduces the electrode-skin impedance, indicating that additional layers contribute electrically to the overall conductive pathway rather than acting as electrically inactive overlays. If the stacked layers were poorly interconnected, the impedance would be expected to remain high or exhibit large variability with layer number. Instead, the smooth dependence of both sheet resistance and electrode-skin impedance on film thickness supports effective interlayer conduction.

4. ML-related data reproducibility. In the sections on electrophysiological recordings and ML inference stability testing, the experiments appear to have been conducted on a single subject (as indicated in the Methods). Given that the multi-day decoder stability in myoelectric control represents a central claim of the study, it would be helpful if the authors could comment on the reproducibility of these results. For example, were additional trials performed (e.g., repeated experiments, different electrode sets, or other users), or can the authors provide further discussion or references supporting the robustness of this approach?

Author Response: We performed substantial repeated experiments (Fig. S21, shown below) using independently fabricated CNT electrode sets placed at the same forearm locations on the same subject. These repeated trials were conducted to determine whether the recalibration-free

behavior observed in the original experiment was reproducible across electrode sets and repeated applications. The added data show that CNT electrodes maintained stable signal quality and decoder performance across repeated sessions, supporting that the observed stability is not specific to a single electrode set or isolated recording.

In the revised manuscript, we also expanded the scope of validation by including longer continuous wear experiments of up to two weeks and a more dexterous myoelectric control task. These additions test the CNT interface under more demanding conditions than the original 48 h center-out task and further support the robustness of the electrode interface for multi-session myoelectric decoding. At the same time, we recognize that these added experiments remain limited in participant number. Therefore, we have revised the manuscript to clarify that the present study is a pilot study to demonstrate within-participant reproducibility and electrode-set reproducibility, while larger multi-participant studies are needed to establish population-level generalizability. We are actively developing a protocol for such studies, and we now explicitly state this limitation and future direction in the revised manuscript.

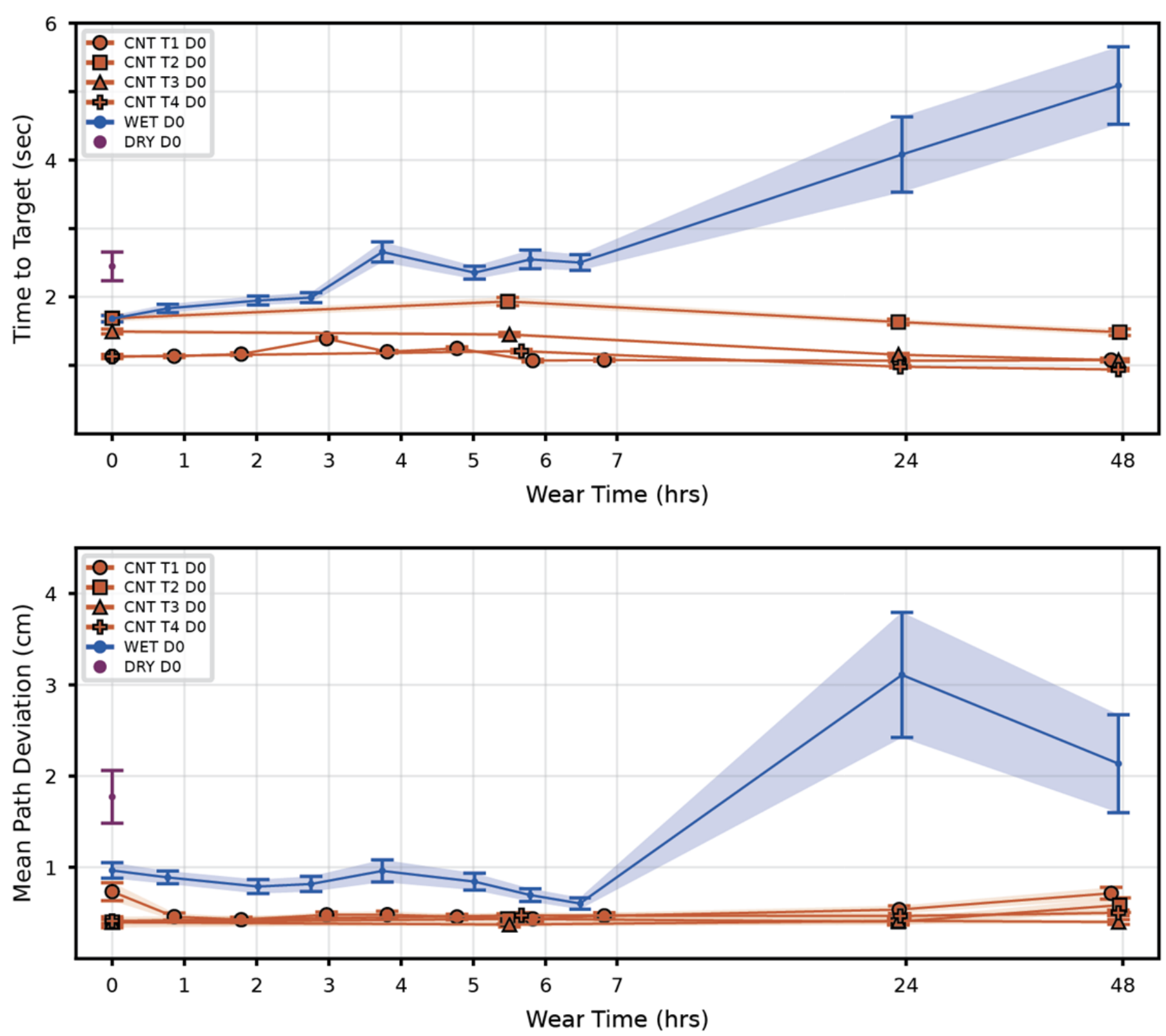

Supplementary Figure S21 | Reproducibility across independent CNT electrode sessions.

The center-out task was evaluated using four independent CNT electrode sessions (T1–T4), with a new CNT electrode set applied for each session. Performance was quantified by time to target and mean path deviation over 48 h of wear and compared with wet and dry electrodes. Both performance metrics, time to target and path deviation, were consistent across all four CNT sessions, demonstrating reproducibility of the results.

5. Sweat effect. The authors suggest that the porosity of the CNT-based electrodes contributes to their stability under conditions relevant to wearable applications, and they provide measurements of water vapor transmission rate to demonstrate breathability. The authors also mention that, thanks to their porous nature, CNT electrodes are resistant to sweat and body heat. However, since these electrodes are intended for long-term skin contact, it would be important to clarify how exposure to sweat may affect their stability and performance. While investigating the effects of sweat may not have been the primary objective of the study, additional discussion supported by experimental data or relevant literature on sweat exposure would strengthen the assessment of their suitability for long-term wearable use.

Author Response: We performed new experiments evaluating CNT electrode performance under sweat, exercise, and shower conditions.

First, we added a sweat and exercise protocol to assess signal stability before and after activities that introduce different forms of mechanical and moisture stress. ECG signals were recorded before and after an outdoor 5 km run to evaluate performance after heavy sweating, after 2 h of indoor rock climbing to evaluate performance following repeated upper-body motion and mechanical perturbation, and after a 30 min hot shower to evaluate performance after elevated skin temperature and extreme external moisture exposure. Across the running and climbing conditions, the CNT electrodes showed minimal changes in ECG signal quality before and after activity, supporting their stability under heavy sweating and intensive movement. After the hot-shower condition, we observed an increase in signal amplitude, which we attribute to improved electrode–skin contact in the wet environment.

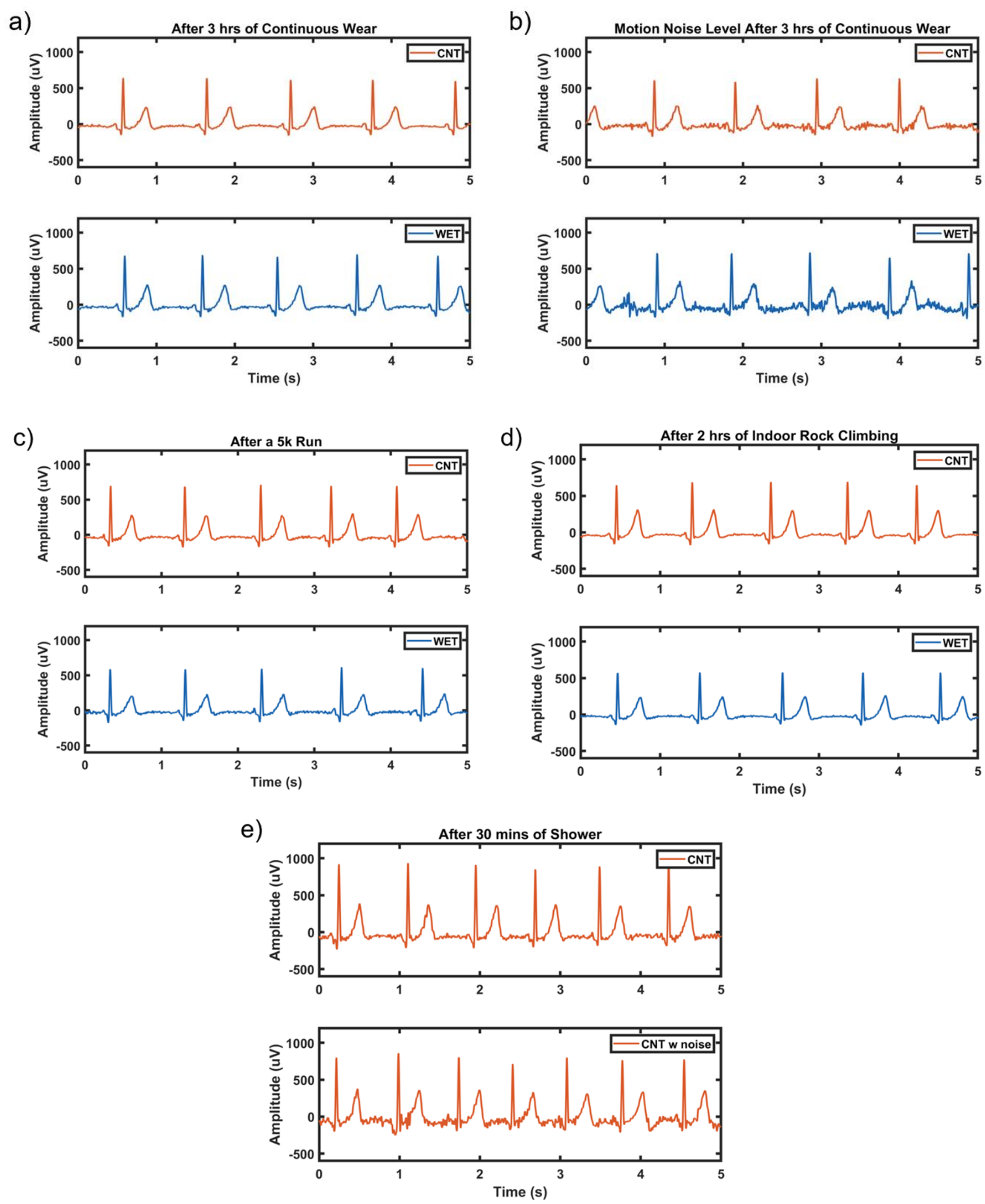

Supplementary Figure S16 | ECG signal stability after sweat, exercise, and shower. **a,** ECG signals after 3 h of continuous wear. **b,** ECG recordings during motion perturbation following 3 h of continuous wear, showing motion-noise response. **c,** ECG signals recorded after an outdoor 5 km run, used to evaluate electrode performance after heavy sweating. **d,** ECG signals recorded after 2 h of indoor rock climbing, used to evaluate stability following repeated upper-body motion, forearm muscle activation, and mechanical perturbation. **e,** CNT ECG signals recorded after a 30 min hot shower, used to evaluate performance after elevated skin temperature and extreme external moisture exposure. Wet electrodes had lost adhesion during the hot shower.

Second, we evaluated the long-term electrochemical stability of CNT films in artificial sweat at 37 °C and compared the results with a representative state-of-the-art conductive polymer electrode material, PEDOT. The CNT films remained electrochemically stable for up to 4 weeks, whereas the PEDOT films exhibited substantial changes after 2 days under the same test conditions. These results support the electrochemical stability of CNT films under warm, moisture-rich conditions relevant to sweat exposure and long-term skin contact.

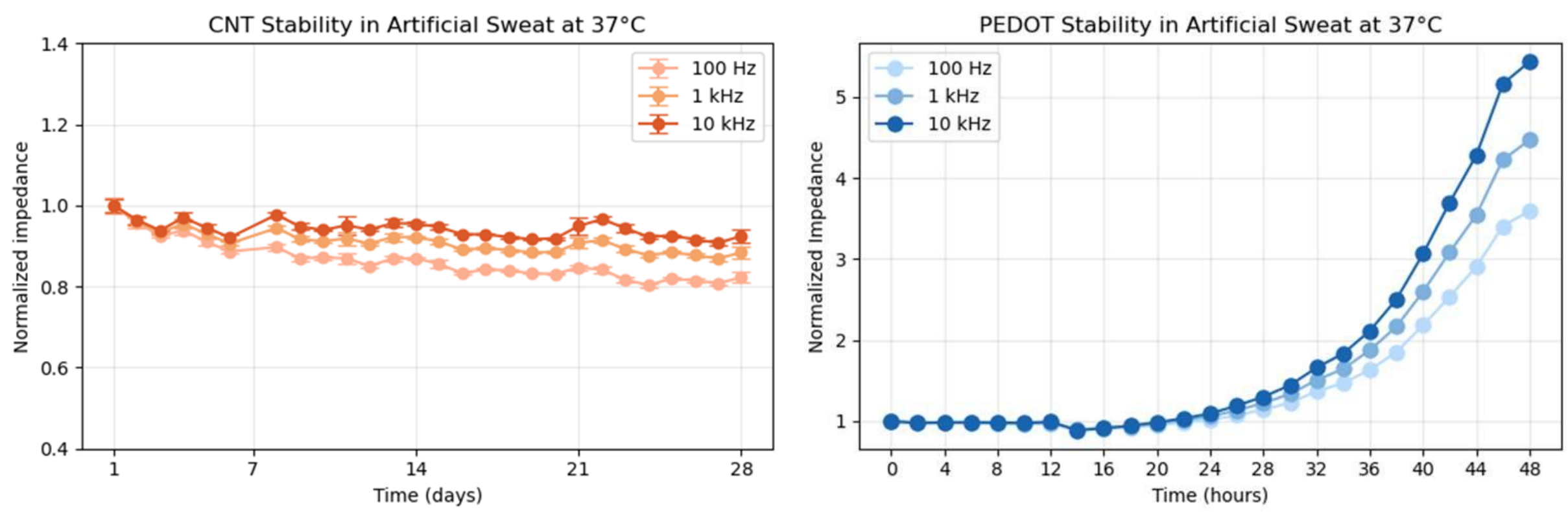

Supplementary Figure S9 | Electrochemical stability of CNT electrodes during continuous immersion in artificial sweat at 37°C compared with PEDOT electrodes.

We have also discussed this limitation in the manuscript that while the present results support CNT electrode stability under exercise, sweating, and shower exposure, future studies should systematically evaluate the effect of sweat and moisture on multichannel ML performance during active use, particularly if decoding is required during or immediately after wet conditions.

Minor comments:

1. The thickness and electrical-optical properties of the electrodes applied in the electrophysiology monitoring and the multi-day decoder test should be explicitly mentioned.

Author Response: We have clarified that the optimized CNT thickness (65%) was used in subsequent experiments on human users.

2. Units should be specified in Table S5. The average values of T and R could also be reported with a consistent number of significant digits.

Author Response: We have revised Table S5 to specify the units for all reported parameters and used a consistent number of significant digits throughout the table.

3. Regarding the use of the term Boolean deposition model, please provide relevant references supporting this terminology and examples where similar approaches (of areal coverage and thickness statistics based on a binary system) have been used in percolative networks, to clarify the statement made in the Supplementary Information.

Author Response: We have included relevant references that describe the mathematical basis, the geometric and probabilistic properties of Boolean models for stochastic geometry and continuum percolation such as 1D connected structures in material science.

4. Photographs (e.g., in the Supplementary Information) of the applied skin electrodes and wiring in the electrophysiological recordings and multiday decoder stability test, particularly the thicker leads connected to the electrodes, would help clarify the overall experimental setup, even though the interconnection method is not the main focus of the study.

Author Response: We have included photographs of the electrodes to better show the connection between the CNT film and the Ag traces (Supplementary Figure S15). For standard bench-top characterizations, such as skin–electrode impedance measurements, conductive fabric or metal foil was connected to the Ag traces using Ag paste. Alligator clips can then be connected to the conductive fabric or metal foil. For instruments using female snap connectors, such as the multiday decoder stability test, male snap connectors were attached to the electrode via Ag paste and mechanical pressure (crimping tool) to provide robust connection.

Reviewer #2 (Remarks to the Author):

Summary

This manuscript presents an innovative solution to the long standing challenges of instability, noise susceptibility, and signal drift in contemporary electrophysiological electrodes. These interfaces are essential for capturing a wide range of biosignals, including electrocardiography (ECG) for cardiac health, electromyography (EMG) for muscle activity, and electroencephalography (EEG) for monitoring brain states, which are increasingly integrated into high dimensional representations of user physiology. A particularly noteworthy aspect of the work is the use of micron thin dry electrodes based on carbon nanotube (CNT) thin films, a material platform that offers mechanical flexibility, conformal skin contact, and enhanced signal fidelity without the need for conductive gels.

The topic is modern and interdisciplinary, effectively integrating materials science with bioelectronic engineering. The long term, recalibration free signal stability demonstrated in the study is particularly impressive and highlights the potential of these CNT-based electrodes for broader use in practical bioelectronic systems.

The figures and diagrams accurately reflect the experimental workflow and outcomes, and the results are presented clearly and coherently. The experiments are well designed, and the data are carefully analyzed, which strengthens the credibility of the conclusions. The manuscript convincingly articulates the need for research in this direction and clearly emphasizes the advantages of the proposed approach.

Minor comments:

Line 115, Page 3: "…three coupled parameters that govern electrode-skin contact quality." - Please specify which parameters are being referred to.

Author Response: We thank the reviewer for pointing this out. We have revised the main text to explicitly specify the three coupled parameters: conformability, network density, and resistance.

Lines 141 - 160, Page 5: The manuscript does not clearly state which synthesis conditions were ultimately used to fabricate the CNT films employed in the electrophysiology experiments.

Supplementary Tables S4 - S5 list representative conditions, and Fig. 2e - f identifies an optimal parameter window, but the exact growth parameters (e.g., gas flow rates, furnace temperature, draw speed) corresponding to the “optimized conditions” are not explicitly provided. For reproducibility, please include the precise synthesis parameters used for all CNT films. Which furnace temperature was optimal? Was the temperature held constant, or do variations in the 1000 - 1200 °C range not affect CNT quality?

Synthesis stability: It may be beneficial to move the synthesis stability assessment from the Supplementary Information into the main text. Demonstrating stability under the selected conditions would directly support the claim of scalability. It may also be useful to mention that sheet resistance drift was quantified and to briefly describe the issue of reactor clogging - how often does it occur, what causes it, and can it be prevented?

Author Response: We have revised the Manuscript to report the synthesis conditions used to fabricate the CNT films for subsequent experiments and explained the clogging issue in the Supplementary Information (Fig. S3). We agree that synthesis-stability assessment strengthens the scalability claim. Given the broad readership of *Nature Communications* and the current length of the manuscript, we kept the synthesis-stability analysis in the Supplementary Information and referred readers to it in the main text.

For a three zone reactor, we found optimal conditions at 1195 °C, 1195 °C and 1160 °C for the top, middle and bottom zones, respectively. We found ideal concentrations of FeCp2 and sulfur to be 4×10^-10 - 1×10^-9 mol/cm^3 and 1×10^-10 - 1×10^-9 mol/cm^3, respectively. The %H2 worked well between 19%-25%, and while some CNT films showed good quality beyond 25% the deposition of carbon on the inlet tube increased. Draw speed was varied with flow rate and diameter of the collecting tube, and was matched as closely as possible with the rate of CNT film emergence from the reactor; we did not observe consistent effects on sheet resistance when varying the draw speed. Flow rate was studied from 1000 sccm – 3000 sccm; at lower flow rates, the relatively cool gas flowing into the system from the gas lines (200 °C), was heated by the tube furnace (1200 °C) to such a degree that there was not significant enough density difference to allow CNT film to sink to the bottom of the vertical tube. At high flow rates, a different problem occurred; the dilution of the reagents was significant, and evaporation of sufficient sulfur became more challenging. Excellent quality films were mostly obtained from 1800 sccm - 2200 sccm total flow.

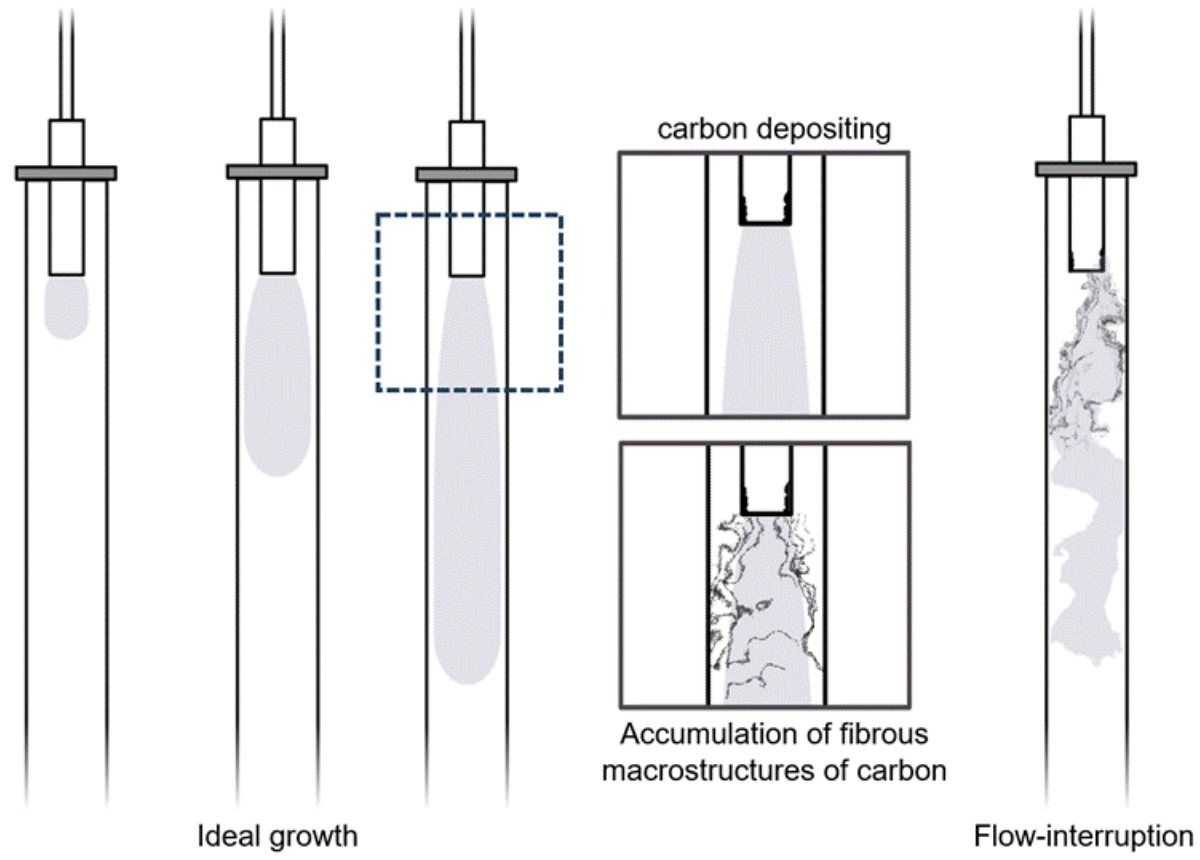

Addressing the comment about the reactor clogging, an illustration is shown in Supplementary Figure S3. Our understanding is that the clogging in the system occurs mainly due to excessive material deposition on the outlet of the inner quartz tube. This inner quartz tube connects the thin stainless-steel gas lines with the 10 cm diameter main reactor tube; it allows the gas stream to flow into the reactor while maintaining a laminar flow. At the outlet of this smaller quartz tube, ferrocene breaks down and iron species are deposited on the quartz surface. There is also decomposition of the carbon species in the presence of hydrogen and sulfur. We will call these deposited materials "fibrous macrostructures"; they contain mainly carbon and iron. The deposition/growth rate of this material is complex; it is affected by the temperature near the outlet, the type of carrier gas and total flow into the system, as well as the concentration of hydrogen and all reagents in the gas stream. As reagents decompose near the outlet of this injector, we observe a significant buildup of these macrostructures. The growing nanotube film is very thin; as such, if it becomes attached to the macrostructures near the outlet injector, it may then be put snagged. Any tension on the film will produce a tear or result in the film becoming stuck halfway in the main reactor tube. Occasionally, the clog may be allowed to build up for several minutes, and when the nanotube film is then dislodged after several minutes some of this macroscopic debris near the outlet of the inner quartz tube will be removed with it. This restores the reactor to normal conditions for some time. If the reactor is operated far outside the normal synthesis window, clogging will occur rapidly especially at higher carbon concentration in the gas stream. Within normal reaction conditions, however, the reactor can be operated for several hours before clogging becomes an issue.

Lines 184 - 188, Page 5 - 6: How does the material perform during intensive patient movement, sweating, or elevated temperatures? Is this relevant for clinical or ambulatory applications?

Author Response: Movement, sweating, and elevated temperature are highly relevant for ambulatory and clinical wearable applications. We added new experiments evaluating CNT electrode performance under sweat, exercise, and shower conditions.

As mentioned in response to review #1 comment 5, we added a sweat and exercise protocol to assess signal stability before and after activities that introduce different forms of mechanical and moisture stress. ECG signals were recorded before and after an outdoor 5 km run to evaluate performance after heavy sweating, after 2 h of indoor rock climbing to evaluate performance following repeated upper-body motion and mechanical perturbation, and after a 30 min hot shower to evaluate performance after elevated skin temperature and extreme external moisture exposure. Across the running and climbing conditions, the CNT electrodes showed minimal changes in ECG signal quality before and after activity, supporting their stability under heavy sweating and intensive movement. After the hot-shower condition, we observed an increase in signal amplitude, which we attribute to improved electrode–skin contact in the wet environment.

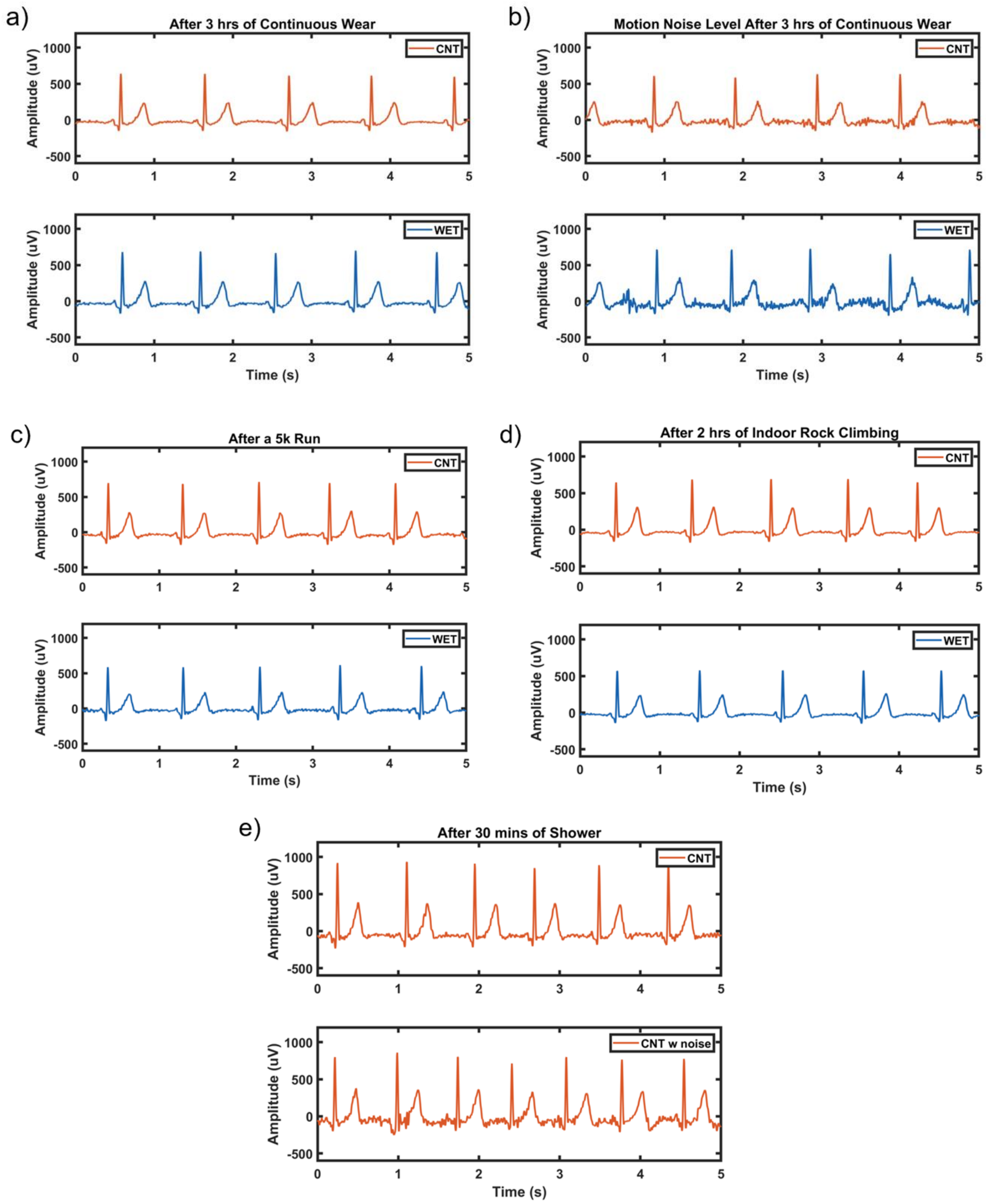

Supplementary Figure S16 | ECG signal stability after sweat, exercise, and shower. **a,** ECG signals after 3 h of continuous wear. **b,** ECG recordings during motion perturbation following 3 h of continuous wear, showing motion-noise response. **c,** ECG signals recorded after an outdoor 5 km run, used to evaluate electrode performance after heavy sweating. **d,** ECG signals recorded after 2 h of indoor rock climbing, used to evaluate stability following repeated upper-body motion, forearm muscle activation, and mechanical perturbation. **e,** CNT ECG signals recorded after a 30 min hot shower, used to evaluate performance after elevated skin temperature and extreme external moisture exposure. Wet electrodes had lost adhesion during the hot shower.

We have also discussed this limitation in the manuscript that while the present results support CNT electrode stability under exercise, sweating, and shower exposure, future studies should systematically evaluate the effect of sweat and moisture on multichannel ML performance during active use, particularly if decoding is required during or immediately after wet conditions.

Second, we evaluated the long-term electrochemical stability of CNT films in artificial sweat at 37 °C and compared the results with a representative state-of-the-art conductive polymer electrode material, PEDOT. The CNT films remained electrochemically stable for up to 4 weeks, whereas the PEDOT films exhibited substantial changes after 2 days under the same test conditions. These results support the electrochemical stability of CNT films under warm, moisture-rich conditions relevant to sweat exposure and long-term skin contact.

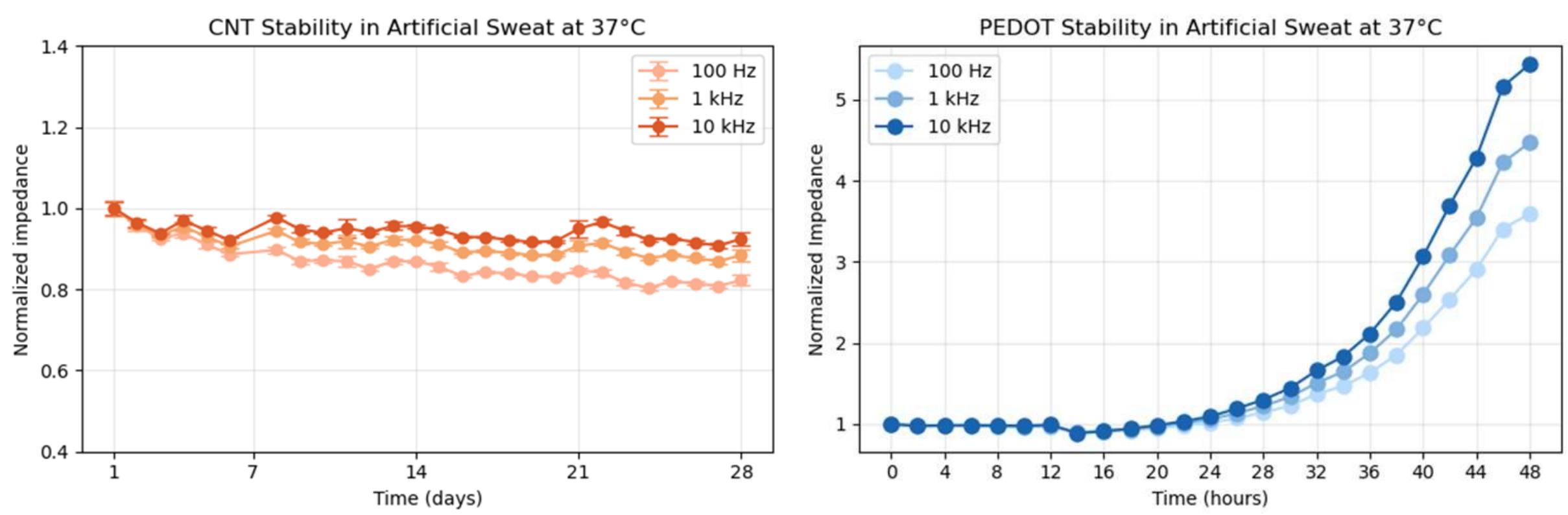

Supplementary Figure S9 | Electrochemical stability of CNT electrodes during continuous immersion in artificial sweat at 37°C compared with PEDOT electrodes.

Line 227, Page 7: The manuscript states that film thickness was controlled by tuning growth conditions, whereas Supplementary Method S1 clarifies that thickness was achieved exclusively through layer by layer stacking of identical films. This discrepancy should be corrected, and the main text should explicitly state that films of varying thickness were not synthesized directly. This is important for readers' understanding of the fabrication process.

Author Response: We have clarified the layer-by-layer assembly process in the main text, which now reads: 'To ensure that intrinsic material properties remain constant throughout the study, thicker CNT films were obtained by stacking single-layer films of 90% T (Method S1).'

Line 248, Page 8: Is it important to measure impedance at frequencies other than 10 Hz? What are the relevant frequency ranges for EEG, EMG, and ECG?

Author Response: Electrophysiological signals are typically low frequency, as summarized in the table below for common ExG signals. There's no consensus in the literature for a single standard frequency to report. For dry electrodes, the electrode–skin interface has a capacitive component, so impedance is higher at low frequencies and decreases at higher frequencies. We selected 10 Hz as the primary comparison frequency, because it lies within the relevant bandwidth of these modalities and provides a stringent low-frequency benchmark. Therefore, achieving low impedance at 10 Hz is more challenging and more informative than comparing only at higher frequencies. The full impedance spectrum was measured from 10 Hz to 1 MHz and is shown in Fig. 3b. The 10 Hz value was used in comparison figures (Fig. 3a) as a representative low-frequency metric for comparing electrode performance across conditions.

Various Electrophysiology Signals

Signal	Source	Magnitude	Frequency
**ECG**	Heart	~1–5 mV	0.05–100 Hz
**EEG**	Brain	~10–100 μV	0.5–70 Hz
**EMG**	Muscle	~1–10 mV	10–2000 Hz
**EOG**	Eye	~0.5–1 mV	DC–10 Hz

Line 269, Page 9: Could you provide quantitative data on CNT migration within tissues? Did the implantation tests include an analysis of material degradation?

Author Response: The biocompatibility of CNT material has long been an active area of research because their interactions with cells and tissues depend sensitively on size, morphology, exposure route, surface chemistry, and dispersion state[2]. We agree that macrophage uptake, migration, and systemic retention are important considerations for CNT-based devices, particularly when CNTs are present as free fragments, dispersed nanotubes, or implantable materials.

In this work, the CNT electrodes are intended for noninvasive epidermal use on intact skin without skin abrasion and phagocytosis does not typically occur under normal skin conditions. The primary goal of biocompatibility assessment in this work is to evaluate whether the specific properties of our synthesized CNT film introduces apparent local cytotoxicity, skin irritation, or tissue immune response under relevant exposure conditions, rather than to re-evaluate the systemic toxicity of CNTs as a nanomaterial class. We have revised the manuscript to clarify this scope and to avoid overgeneralizing the biocompatibility results.

Accordingly, the subcutaneous implantation experiment was included as a conservative local tissue-response stress test to reveal any unexpected tissue reaction uniquely associated with our synthesized CNT films. The films used in this study are pristine, unmodified, undispersed, and mechanically interconnected during FCCVD synthesis. One concern specific to our CNT materials is residual catalyst contamination, because metal impurities such as Fe, Ni, and Co have been associated with inflammatory responses. In our CNT films, the residual Fe catalyst particles are encapsulated within graphitic carbon shells, as shown in Fig. 2c, which reduces direct catalyst–tissue interaction. The implantation experiment serves to test whether the CNT film and the encapsulated residual catalyst produce an abnormal local immune response under direct tissue contact.

A second concern specific to our CNT electrodes is the possibility of CNT residue or fragment release during repeated mechanical deformation or removal from skin. To address this point, we added structural-stability analysis after cyclic mechanical strain. Specifically, we performed tacky-transfer tests of CNT films after 1000 stretch–release cycles at 35% strain. The 5-layer film (~60% T) corresponds to the optimized thickness used in the human electrophysiology studies. SEM images of the transfer surface showed no observable CNT fragments or CNT residues after mechanical cycling, supporting that the interconnected CNT network maintains structural integrity and does not readily shed detectable nanotube debris under the tested conditions.

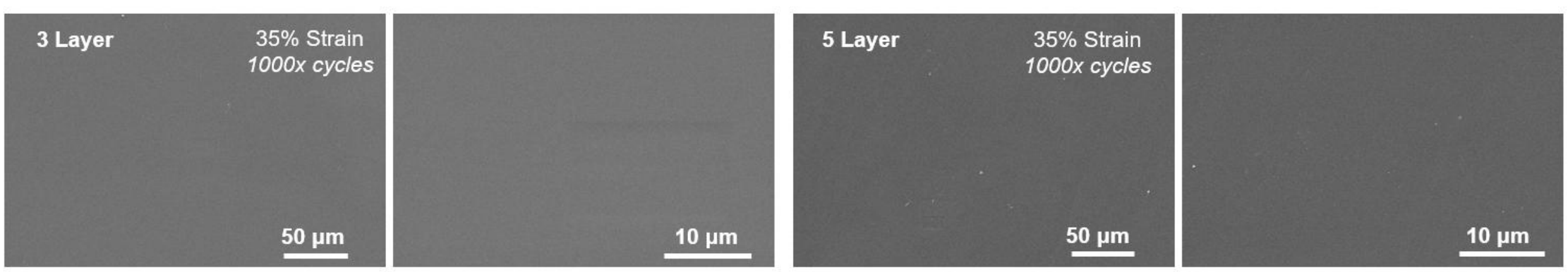

Line 293, Page 10: Would it be valuable to extend the skin compatibility (irritation) test beyond 3 h, for example to 48 h? This would allow a more direct comparison with wet electrodes, which were tested for 48 h, and would better support the claim of “long term skin compatibility”.

Author Response: We thank the reviewer for pointing out this discrepancy in wear duration. We added photographs of the skin condition after three days of continuous CNT wear to Fig. 4k. After removal of the CNT film and gentle cleaning of the skin, no visible CNT residue remained on the skin surface, and no visible erythema, irritation, or skin damage was observed.

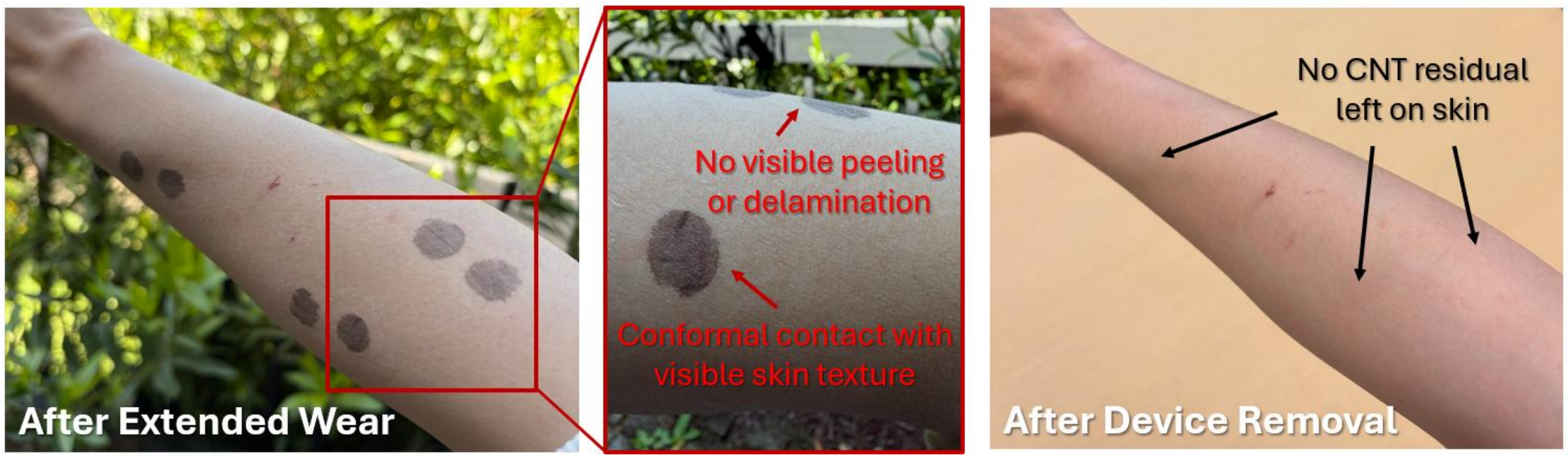

Line 304, Page 11: Would it be useful to include a control condition using other types of dry electrodes?

Author Response: We used wet Ag/AgCl electrodes as the primary control because they remain the most standardized and widely used benchmark for ECG, EMG, and EEG recordings. Dry electrodes are less standardized, and their performance depends strongly on material, geometry, attachment method, applied pressure, and recording location. The dry electrode we chose (Dryode) is the best-performing and most commercially accessible dry electrode we identified. It serves to provide a general idea of typical dry electrode performance. Research-grade dry electrodes usually have better performance and we have compiled literature benchmarking of state-of-the-art dry electrodes in the Supplementary Information (Table S2-S4), to provide a broader comparison of CNT electrodes with existing technologies.

Line 342, Page 13: Please clarify why these electrode placement sites were chosen. Could the test be performed at other locations?

Author Response: The five pairs of surface electrodes were placed to target five major muscle groups: pronator teres (pair 1), extensor pollicis longus/brevis (pair 2), flexor carpi (pair 3), extensor carpi (pair 4), and extensor digitorum (pair 5). These muscles were selected because they provide separable EMG patterns for two-dimensional myoelectric cursor control[3].

The test can in principle be performed at other anatomical locations, provided that the electrode placement captures task-relevant and sufficiently independent muscle activity. This would require future studies to use high-density sEMG mapping and identify non-redundant electrode locations and adapt the electrode layout for specific consumer, clinical or rehabilitation applications, such as impaired muscle groups in stroke patients.

Line 346, Page 13: During the multi day stability tests, were the electrodes removed and reapplied (e.g., during showering)? If so, please provide data. Did electrode displacement occur, or were they kept in the exact same position throughout?

Author Response: The electrodes were worn continuously throughout the multiday stability tests, including during shower and sleep. No electrode dislocation was observed during the study duration. We have revised the manuscript to clarify this experimental setup.

Line 357, Page 13: Was the multi day MI test performed on only one participant?

Author Response: Yes, the multi-day myoelectric-interface test was performed as a pilot study in one participant. We have clarified this in the revised manuscript. To strengthen the reproducibility of the result, we added repeated experiments using newly fabricated CNT electrode sets applied to the same forearm. At the same time, we recognize that these added experiments remain limited in participant number. Therefore, we have revised the manuscript to clarify that the present study is a pilot study to demonstrate within-participant reproducibility and electrode-set reproducibility, while larger multi-participant studies are needed to establish population-level generalizability. We are actively developing a protocol for such studies, and we now explicitly state this limitation and future direction in the revised manuscript.

Sample size: The number of samples (n) for SEM, TEM, Raman, WVTR, and mechanical testing is not provided. Please include this information for reproducibility.

Author Response: We have revised the Methods section to include the relevant (n) values for SEM, TEM, Raman, WVTR, and mechanical testing.

Impedance statistics: Please provide full statistical information for impedance measurements (n, SD, statistical tests). In Fig. 3h, the number of repetitions is missing.

Author Response: We have revised the manuscript to include n, mean +/- sd for contact impedance and n for mechanical disturbance tests (Fig. 3h).

Limitations: Please discuss potential limitations of the study, such as the absence of clinical testing and the lack of long term (>72 h) evaluations.

Author Response: We have revised the manuscript to more explicitly state the limitations and application boundaries of the present study.

The myoelectric decoding experiments were performed as pilot demonstrations. Larger-cohort user studies and clinical trials are needed to establish population-level generalizability, particularly in rehabilitation populations or users with impaired neuromuscular control. We have also addressed the concern regarding long-term evaluation. In the revised manuscript, we extended the longest continuous-wear demonstration beyond the original 48 h period to 14 days and evaluated both the original center-out cursor-control task and a more challenging obstacle-avoidance task using the same initially trained decoder. We also clarified that the practical lifetime is limited by device-level integration, including interconnect fatigue, substrate degradation, and gradual changes at the skin surface. Future development of high-performance flexible encapsulation and interconnect layers will be needed to realize a fully integrated long-term wearable device.

Verdict: Acceptance with minor revisions.

Reviewer #3 (Remarks to the Author):

Yunhan Wen et al. reported the preparation, characterization and application of carbon nanotube (CNT) thin film dry electrodes, and realized a recalibration-free myoelectric interface for long-term electrophysiological monitoring. The study content is detailed and the technical route is relatively complete. However, the manuscript still needs major revision in several aspects such as structural organization, data interpretation and comparative analysis. Specific comments are as follows:

1. The overall structure of the manuscript hinders readability. For instance, the Introduction is overly lengthy and should be condensed. The paragraph immediately following the "Results and Discussion" heading reads more like a summary of the entire study and could be integrated into the end of the Introduction (indeed, it partially overlaps with the final two paragraphs of the Introduction). If the authors prefer to retain this paragraph in the Results section, the corresponding conclusions must be supported by specific data. For example, when reading the sentences, "The biocompatibility of the CNT film is validated through in vitro cytotoxicity assays, skin patch test, and subcutaneous implantation test on mice models. Electrode performance in electrophysiological recordings of ECG, EEG, and EMG signals, demonstrated improved signal fidelity compared to conventional wet electrodes," no citations to corresponding figures or data are provided. It is strongly recommended that the authors add detailed citations to figures and data throughout the main text to help readers quickly locate supporting evidence, particularly for the "Electrode Impedance and Optimization" section and Figure 3.

Author Response: We have substantially revised the manuscript structure to focus more directly on the key motivation, knowledge gap, and novelty of the work. We also removed redundant summary text from the beginning of the Results and Discussion section. Detailed figure citations were added throughout the main text to improve readability and traceability. In particular, we revised the Electrode Impedance and Optimization section to include clearer references to the relevant panels in Fig. 3 and the Supplementary Information, so that readers can more easily locate the data supporting each conclusion.

2. The authors compared the CNT electrodes with PEDOT, metal nanowire, and Ag/AgCl wet electrodes, but neglected the comparison with many emerging flexible electrodes in recent years, such as conductive hydrogel electrodes, liquid metal flexible electrodes, and flexible electrodes fabricated by semiconductor micro/nano processes. It is suggested to supplement a multi-

dimensional comparison with these electrodes in terms of impedance, signal-to-noise ratio, wearing duration, biocompatibility, etc., to clarify the core competitive advantages and shortcomings of the CNT electrodes.

Author Response: We have included multi-dimensional comparison across several classes of flexible and dry electrodes in the Supplementary Information (Supplementary Table S2-S4). The compared metrics are form factor, contact impedance, signal quality, mechanical stability, motion noise, breathability, wear duration and ML-related performance or stability when available. The comparisons are divided into three categories: nanomaterial-based dry electrodes (Supplementary Table S2), organic and metal thin-film electrodes (Supplementary Table S3), and other emerging electrode technologies (Supplementary Table S4) such as conductive hydrogels, liquid metals, and micromachined electrodes. We have also revised the main text to summarize the key findings from these expanded comparisons.

3. Related to the above point, the authors mention in Supplementary Tables S2-S3 that the tensile performance of some metal electrodes can reach 500%. However, the comparison in Figure 2k only includes metal nanowire electrodes. This selective comparison, which appears to highlight the advantages of CNT thin films, should be revised to include a broader and more representative range of devices.

Author Response: We thank the reviewer for pointing out that the original comparison in Fig. 2k could be interpreted as selective. We agree that metal-based electrodes and other flexible electrode architectures can achieve very high tensile strain, including values approaching or exceeding several hundred percent, when structural designs, such as serpentine traces, kirigami patterns, mesh geometries, or ultrathin microfabricated layouts, are used.

Because this figure is presented in the context of CNT synthesis and material properties, we believe it is more appropriate to compare material-level properties. We have revised the Fig. 2k, its caption, and the main text to make the distinction between material properties and engineered properties. This distinction is important because structural design can dramatically improve the apparent stretchability of many otherwise stiff materials. For example, conventional metals or silicon-based materials can become highly flexible or stretchable when patterned into ultrathin, serpentine, or mesh geometries[4]. These strategies are powerful, but they reflect device architecture rather than the intrinsic mechanical behavior of the conductive material itself.

To provide more context, we pointed the readers to Supplementary Tables S2-S4, which compare more comprehensive and device-level metrics (e.g. form factor, contact impedance, signal quality, mechanical properties, motion noise, breathability, wear duration, biocompatibility, and ML-related performance where available).

Another example of such distinction is the higher sheet resistance of CNT thin films compared to metals. However, in electrophysiological recording, the electrode–skin contact impedance is often on the order of kΩ to MΩ, so sheet-resistance differences on the order of hundreds of ohms are not usually the dominant limitation once the CNT film is sufficiently conductive and well coupled to the skin. The advantage of CNT films in this work is their combination of dry conformal contact, porosity, breathability, mechanical compliance, and stable multichannel signal structure during long-term wear.

Another limitation of CNT films is that metal deposition and microfabrication methods are more mature and standardized than traditional FCCVD CNT synthesis. One contribution of this work is therefore to demonstrate a scalable method for producing free-standing, percolated CNT thin films compatible with roll-to-roll collection.

4. All experiments were conducted under normal, static conditions, with the only motor test being repetitive wrist movement. The performance stability of the electrodes under more challenging scenarios (e.g., during strenuous exercise or heavy sweating) was not investigated. It is recommended to supplement the study with performance tests under such conditions to better define the application boundaries of the electrodes.

Author Response: This is an important point for defining the practical operating conditions of the CNT electrodes. As mentioned in response to Review #1 Comment 5, we added performance tests under more challenging activity and moisture conditions, including an outdoor 5 km run, 2 h of indoor rock climbing, and a 30 min hot shower.

These tests were selected to represent distinct stressors relevant to ambulatory use. The 5 km run was used to induce heavy sweating and whole-body motion; the rock-climbing session was used to introduce repeated upper-body movement, forearm muscle activation, skin deformation, and mechanical perturbation near the recording sites; and the hot-shower condition was used to evaluate the effect of elevated skin temperature and extreme external moisture.

ECG signals were recorded before and after each activity. After both running and climbing, the CNT electrodes maintained comparable signal quality, with minimal changes in waveform morphology and signal amplitude. These results indicate that the electrodes remain functional after intensive movement and heavy sweating. After the hot shower, we observed an increase in signal amplitude, which we attribute to improved electrode–skin contact under hydrated conditions.

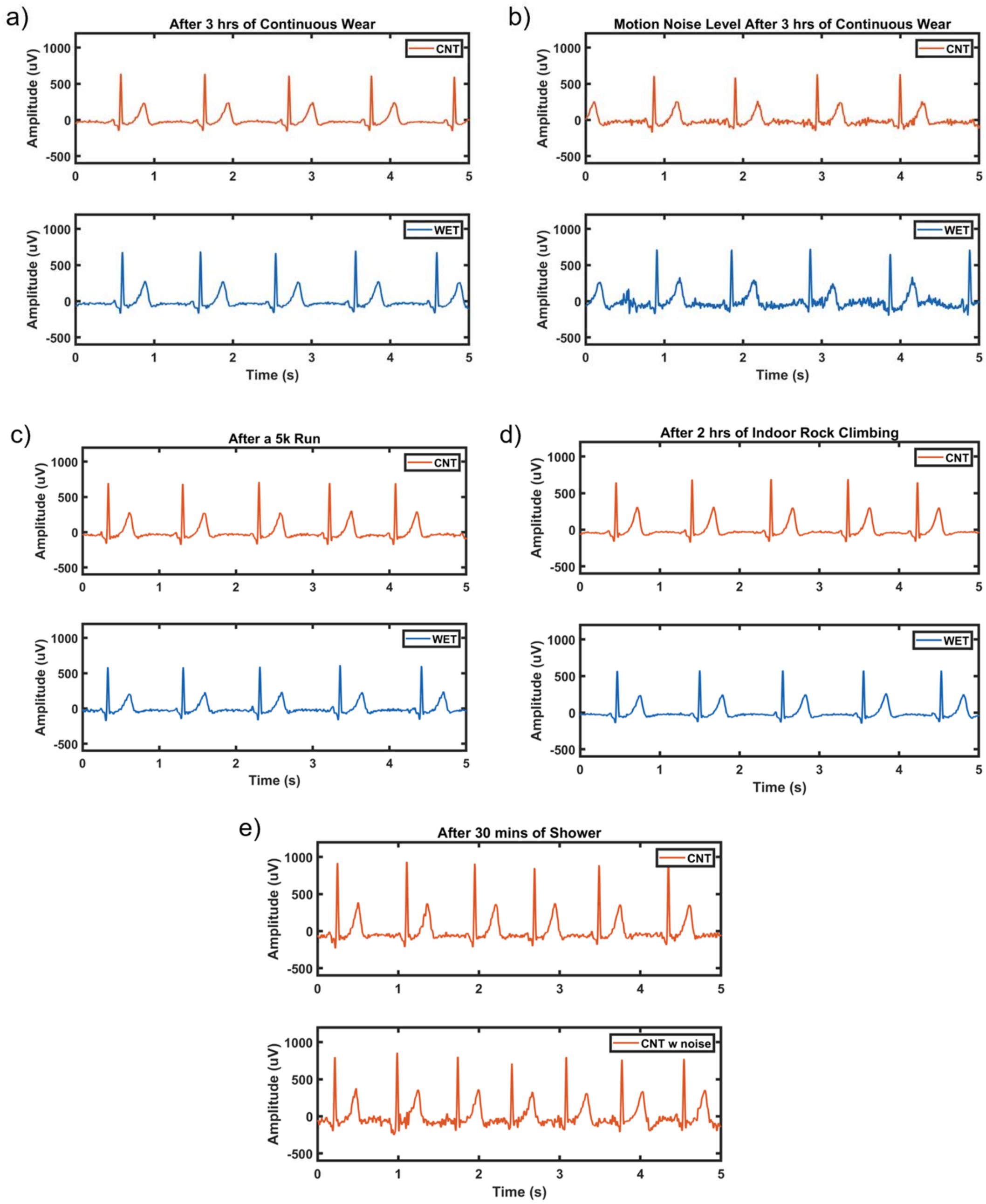

Supplementary Figure S16 | ECG signal stability after sweat, exercise, and shower. **a,** ECG signals after 3 h of continuous wear. **b,** ECG recordings during motion perturbation following 3 h of continuous wear, showing motion-noise response. **c,** ECG signals recorded after an outdoor 5 km run, used to evaluate electrode performance after heavy sweating. **d,** ECG signals recorded after 2 h of indoor rock climbing, used to evaluate stability following repeated upper-body motion, forearm muscle activation, and mechanical perturbation. **e,** CNT ECG signals recorded after a 30 min hot shower, used to evaluate performance after elevated skin temperature and extreme external moisture exposure. Wet electrodes had lost adhesion during the hot shower.

In addition, we evaluated the long-term electrochemical stability of CNT films in artificial sweat at 37 °C and compared the results with a representative state-of-the-art conductive polymer electrode material, PEDOT. The CNT films remained electrochemically stable for up to 4 weeks, whereas the PEDOT films exhibited substantial changes after 2 days under the same test conditions. These results support the electrochemical stability of CNT films under warm, moisture-rich conditions relevant to sweat exposure and long-term skin contact.

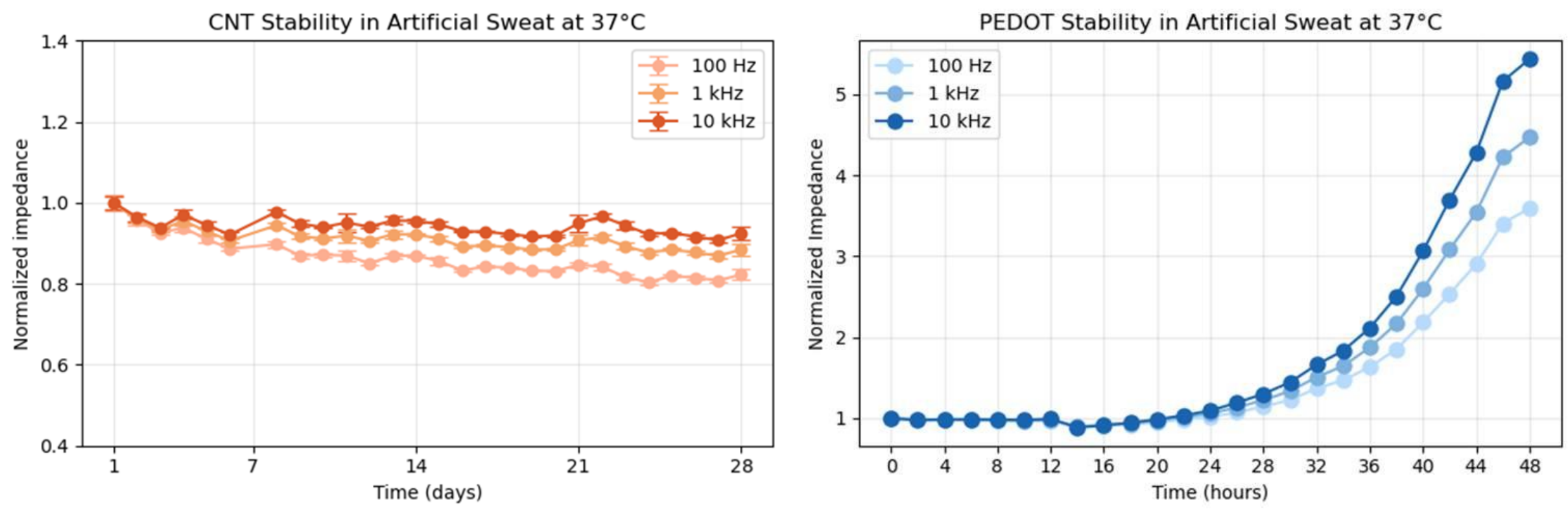

Supplementary Figure S9 | Electrochemical stability of CNT electrodes during continuous immersion in artificial sweat at 37°C compared with PEDOT electrodes.

We have also discussed this limitation in the manuscript that while the present results support CNT electrode stability under exercise, sweating, and shower exposure, future studies should systematically evaluate the effect of sweat and moisture on multichannel ML performance during active use, particularly if decoding is required during or immediately after wet conditions.

5. While the authors compared the skin irritation of different electrodes after continuous wear in the biocompatibility assessment, they did not investigate the long-term electrical stability of the CNT electrodes after prolonged use. It is crucial to characterize the electrical properties after long-term wearing experiments, analyze potential failure mechanisms, and verify whether the claimed "low skin–electrode impedance and high signal fidelity" can be maintained over extended periods.

Author Response: The reviewer raised an important point on characterizing long-term electrical stability of the proposed CNT electrodes after prolonged wear. To address this point, we added additional measurements of skin–electrode impedance and signal-to-noise ratio over an extended wear period. These measurements show that the CNT electrodes maintain low skin–electrode

impedance and stable signal quality after prolonged skin contact, supporting that the electrical performance is preserved during long-term wear.

To address this point, we added longitudinal plots of skin–electrode impedance (Fig. 5L) and showed EMG modulation variation (Fig. S19) and signal drift (Fig. S20) for CNT and wet electrodes. The CNT electrodes maintained relatively stable impedance and SNR over the extended wear period. The wet electrodes could not be evaluated for longer than 48 h duration because prolonged wear posed a significant risk of skin irritation to the participant, that we have observed in the past (Fig. 4). For the 48 h wet-electrode comparison, the wet electrodes were covered with a waterproof layer to preserve the hydrogel from swelling and losing adhesion during showering. As a result of this occlusive condition, we observed a decrease in wet-electrode impedance over time, likely due to increased hydration of the skin. However, such reduction in impedance did not translate into stable myoelectric decoding performance because of the large channel specific impedance variation over time. The wet-electrode experiments showed substantial instability within 48 h, including increased failure rate in completing the task and degraded performance metrics. This result highlights that impedance alone is not sufficient to predict ML-relevant interface stability. Stable myoelectric decoding requires preservation of the multichannel EMG feature distribution, including relative channel amplitudes, timing, noise level, and electrode-specific activation patterns. These results are consistent with a recent HDEMG study[1] showing that modest average impedance changes and stable SNR can coincide with statistically significant electrode-specific impedance variability, which significantly decreased the accuracy of gesture recognition for classifiers that rely on consistent patterns of sensed myoelectric activity over time.

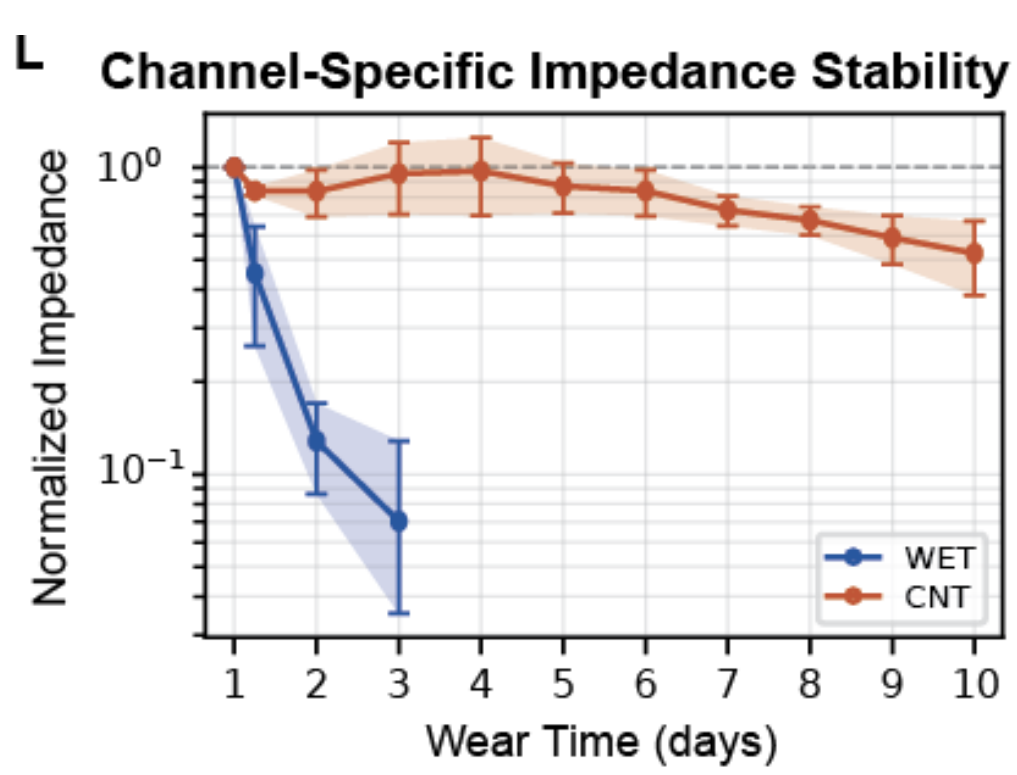

Figure 5L | Normalized channel-specific impedance variation over 10-day wear for wet (n = 8) and CNT electrodes (n = 10).

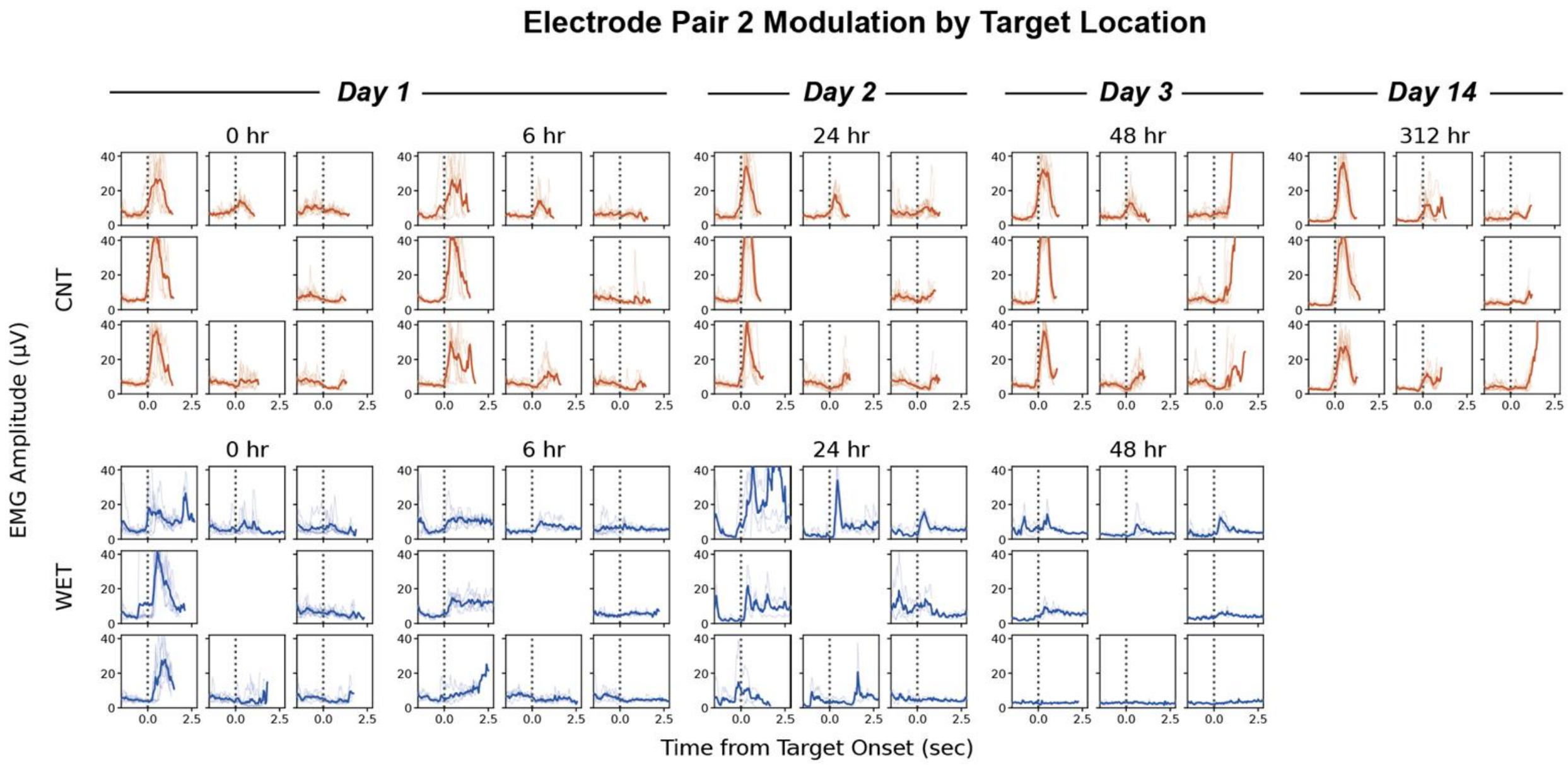

Supplementary Figure S19 | EMG modulation from electrode pair 2 over extended wear, grouped by target location. EMG RMS traces from electrode pair 2 are shown for CNT dry electrodes and wet gel electrodes across cursor control sessions at 0, 6, 24, 48, and 312 h after initial electrode placement. Wet electrodes failed after 48 hours. Each small panel corresponds to one target location, arranged according to the center-out target layout. CNT electrodes preserved clear target-dependent modulation from Day 1 to Day 14, whereas wet electrodes showed significant variation in modulation patterns from Day 1 to Day 3. Dark traces represent the average values, and lighter traces represent individual trials. The vertical dashed line marks target onset.

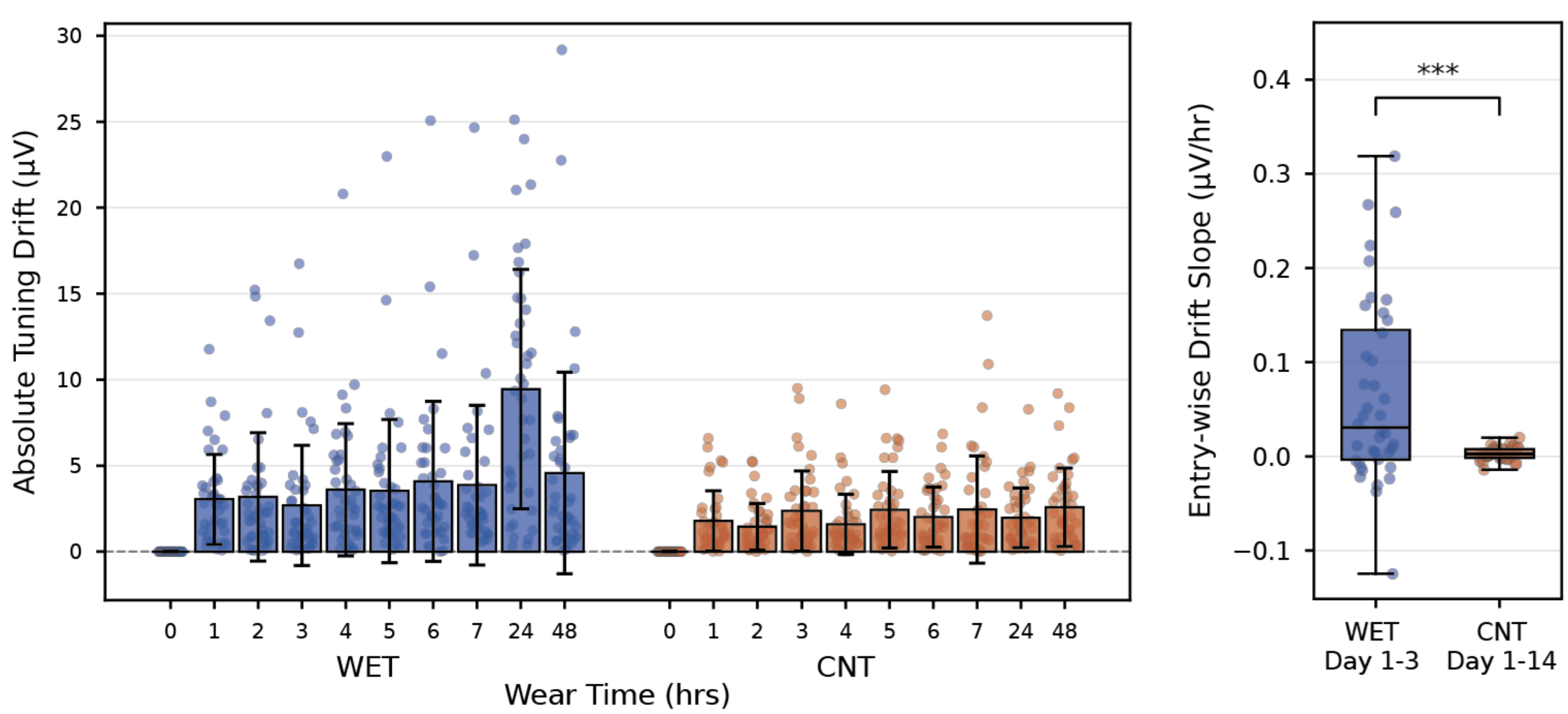

Supplementary Figure S20 | EMG tuning drift during extended wear. Left, absolute EMG tuning drift was quantified over time for wet gel electrodes and CNT dry electrodes. Points represent individual measurements. Wet electrodes showed larger and more variable tuning drift over the first 48 h, whereas CNT electrodes maintained lower drift. Right, tuning drift slopes were compared for wet gel electrodes and CNT dry electrodes. CNT electrodes were calculated across 2 weeks of wear whereas the wet electrodes were measured across 2 days and failed afterwards. CNT electrodes exhibited significantly smaller drift slopes over Day 1–14 than wet electrodes over Day 1–3, indicating improved stability of target-dependent EMG tuning during prolonged wear.

We also discussed potential failure mechanisms in the revised manuscript. Based on our observations, the CNT film itself is not the primary failure point: instead, possible long-term failure modes are more likely to arise from device-level integration, including the transition between stretchable, flexible, and rigid components, the electrical interconnects, and degradation of the temporary substrate/adhesive used for skin attachment. In addition, because the epidermis continuously renews over time, periodic electrode replacement or reapplication may be necessary to maintain consistent skin contact. Based on our extended-wear results, we now recommend a practical replacement interval on the order of two weeks for the current device configuration.

6. The biocompatibility assessment after subcutaneous implantation, which only used CD3 and CD68 markers, is insufficient. Given that CNTs have the potential to be phagocytosed by macrophages and retained in organs like the liver and spleen, a more comprehensive evaluation is necessary. It is suggested to include the detection of inflammatory factors such as TNF-α, IL-1β, and IL-6, quantify the CNT content in various organs, and compare the structural and morphological changes of the CNT thin films before and after implantation.

Author Response: The biocompatibility of CNT material has long been an active area of research because their interactions with cells and tissues depend sensitively on size, morphology, exposure route, surface chemistry, and dispersion state[2]. We agree that macrophage uptake, migration, and systemic retention are important considerations for CNT-based devices, particularly when CNTs are present as free fragments, dispersed nanotubes, or implantable materials.

In this work, the CNT electrodes are intended for noninvasive epidermal use on intact skin without skin abrasion and phagocytosis does not typically occur under normal skin conditions. The primary goal of biocompatibility assessment in this work is to evaluate whether the specific properties of our synthesized CNT film introduces apparent local cytotoxicity, skin irritation, or tissue immune response under relevant exposure conditions, rather than to re-evaluate the systemic toxicity of CNTs as a nanomaterial class. We have revised the manuscript to clarify this scope and to avoid overgeneralizing the biocompatibility results.

Accordingly, the subcutaneous implantation experiment was included as a conservative local tissue-response stress test to reveal any unexpected tissue reaction uniquely associated with our synthesized CNT films. The films used in this study are pristine, unmodified, undispersed, and mechanically interconnected during FCCVD synthesis. One concern specific to our CNT materials is residual catalyst contamination, because metal impurities such as Fe, Ni, and Co have been associated with inflammatory responses. In our CNT films, the residual Fe catalyst particles are encapsulated within graphitic carbon shells, as shown in Fig. 2c, which reduces direct catalyst–tissue interaction. The implantation experiment serves to test whether the CNT film and the encapsulated residual catalyst produce an abnormal local immune response under direct tissue contact.

A second concern specific to our CNT electrodes is the possibility of CNT residue or fragment release during repeated mechanical deformation or removal from skin. To address this point, we added structural-stability analysis after cyclic mechanical strain. Specifically, we performed tacky-transfer tests on 3-layer and 5-layer CNT films after 1000 stretch–release cycles at 35% strain, where the 5-layer film corresponds to the optimized thickness used in the human electrophysiology studies. SEM images of the transfer surface showed no observable CNT fragments or CNT residues after mechanical cycling, supporting that the interconnected CNT network maintains structural integrity and does not readily shed detectable nanotube debris under the tested conditions.

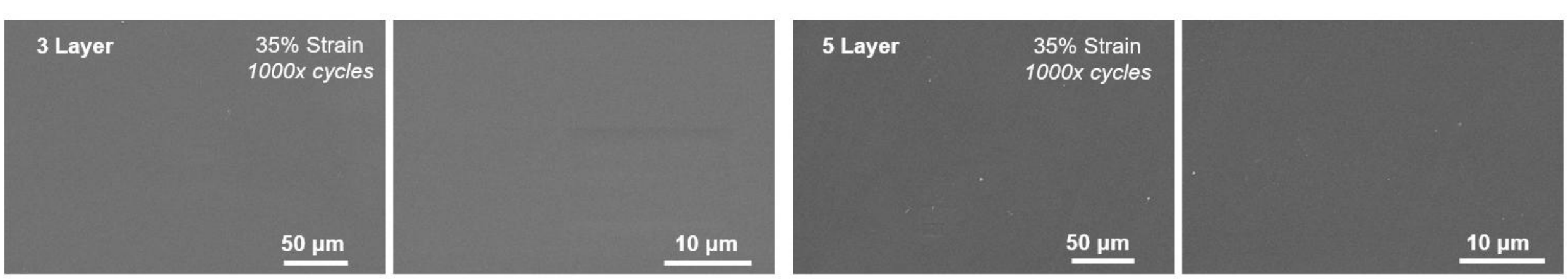

7. Several issues exist in the figures and statistical analysis:

(1) Fig. 1b: The specific type of skin replica used is not specified, and the SEM image lacks detailed information. It is recommended to provide SEM images of the skin replica with half the area covered by the CNT thin film and the other half uncovered, including magnified images of the edge between the two areas. This would more clearly demonstrate the conformability of the CNT film to the skin.

Author Response: We have revised the Methods section to specify how the skin replicas were prepared. We have also added new SEM images of CNT films with ~60% T (a) and ~30% T (b) on skin replica in the Supplementary Information. The skin replica is only partially covered by the CNT film. The red arrows indicate the transition between CNT-covered and uncovered regions. Because the CNT films are nanometer thin and very porous and placed on polymers (skin replica), they are sensitive to charging and high bean energy. We sputtered 5nm of Au/Pt prior to SEM imaging to minimize disturbance to the fine structures and improve image quality.

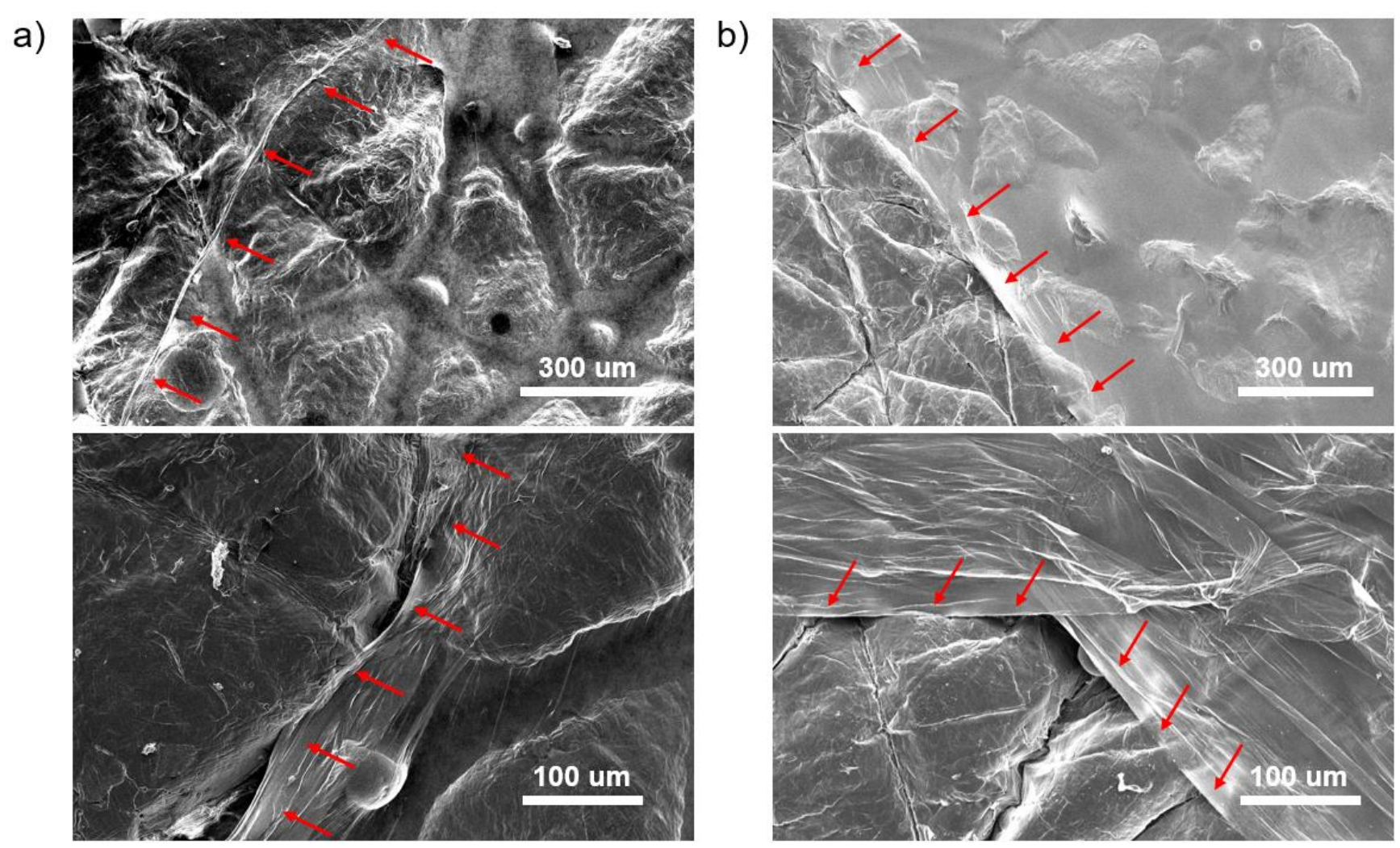

(2) Fig. 2e: Please add units to the color gradient scale.

Author Response: We have added units for equivalent sheet resistance to the color gradient scale.

(3) Fig. 3: The text describes Figure 3b before Figure 3a, which is inconsistent with the figure's sequence. Please reorder the text or the figure panels accordingly.

Author Response: We have revised the main text to describe these figures in order.

(4) Fig. 3d: The SEM image quality is poor. The specific type of skin surface used for the SEM test is not indicated, and the image fails to clearly illustrate why the CNT film with 30% transmittance cannot conform well to the skin's micro-valleys.

Author Response: We have discussed suggestions regarding the SEM images in our response to Comment 7(1). In addition, all CNT films studied here are nanometer-thick and highly conformal; the thickest films still showed lower contact impedance than wet electrodes (Fig 3a-b). The 30% transmittance film shows a reduced conformability relative to thinner CNT films, rather than being non-conformal. We have detailed quantitative analysis of the conformality in the supplemental information, including experimental results and modeling.

(5) Statistics: The statistical analysis methods and sample sizes (n values) are not clearly stated for some experimental data, which diminishes the rigor of the results.

Author Response: We have revised the main text, figure captions, and methods to more clearly state the statistical analysis methods and sample sizes for the relevant experiments.

Reviewer #4 (Remarks to the Author):

This manuscript presents a comprehensive engineering effort to develop carbon nanotube (CNT) thin-film-based dry electrodes for long-term electrophysiological monitoring.The work is technically sound, the experimental data are robust, and the demonstration of a 48-hour, recalibration-free myoelectric interface is a notable and practically relevant result. The systematic approach from scalable material synthesis and optimization of the electrode-skin interface to final system-level validation is a strength. However, as the editor and reviewer have highlighted, the degree of conceptual novelty requires careful scrutiny to meet the high bar of Nature Communications.While the individual components (CNT films, dry electrodes, myoelectric interfaces) are not new,the integration and the specific demonstration of long-term signal stability for ML inference without retraining could constitute an advance.Therefore, my major critiques center on clarifying and strengthening this claim of novelty, ensuring robust comparisons, and bolstering the mechanistic insights.

Major comments:

1.The manuscript demonstrates continuous electrode wear for three days and interprets this as evidence of long-term stability. However, several previously reported dry electrode materials, such as PEDOT:PSS and silver nanowire networks, have also demonstrated stable electrophysiological recordings over multiple days. Therefore, it is unclear whether a three-day test is sufficient to support the claim of "long-term stability."Could the authors clarify the expected performance limits of the proposed electrode? If the system requires retraining after only three days of operation, the practical advantage over existing dry electrodes may be limited. Additional validation over longer time scales (for example, several weeks) would strengthen the claim of long-term stability.

Author Response: We have extended the study duration to two weeks and revised the manuscript to more clearly define the stability metric evaluated in this work and to distinguish conventional electrophysiological signal stability from ML-relevant decoder stability.

For the long-term stability characterization, a key distinction of this work is that we evaluate electrode stability using task-level metrics that are more representative of real-world ML-driven interfaces and more stringent than conventional electrophysiological benchmarks. Prior studies commonly define long-term stability by the continued ability to record ExG signals, maintain low impedance or SNR, or preserve visually similar waveforms. While important, these metrics do not

necessarily ensure stable downstream performance in multichannel ML systems. In high-channel-count systems, each channel must remain stable not only individually, but also relative to each other. Small channel-specific changes in electrode–skin impedance, signal amplitude, baseline noise, or motion artifacts can shift the multichannel feature distribution and substantially degrade task-level performance, even when individual waveforms remain distinguishable. A recent HDEMG study[1] similarly showed that despite modest average impedance changes and stable SNR, statistically significant electrode-specific impedance variability was observed. Although these changes did not significantly affect the overall SNR, they decreased the accuracy of gesture recognition for classifiers that rely on consistent patterns of sensed myoelectric activity over time. Recalibration after approximately 1 h restored much of the lost accuracy. This supports our use of ML task performance as a more stringent metric of electrode–interface stability than impedance or SNR alone. In addition, the probability of system failure increases significantly with the number of electrodes. Our myoelectric interface has 10 unipolar channels (5 bipolar pairs) targeting five distinct muscle groups. Achieving stable task performance across this multi-channel array for 14 days without retraining is a significantly higher bar than single-channel SNR stability.

This distinction in ML-level stability metrics is especially important for continuous myoelectric decoding. Although some studies have demonstrated multi-day gesture classification, this task maps EMG activity to a finite set of discrete labels and can therefore tolerate some signal drift, as long as the class boundaries remain separable. On the other hand, continuous decoding directly maps EMG features to real-time cursor kinematics such as direction and velocity. As a regression task performed in closed loop, it is more sensitive to small changes in signal amplitude, timing, and inter-channel relationships. Users also adapt their muscle activation in real time based on decoder output. Therefore, electrode drift can disrupt not only the input feature distribution, but also the user–decoder control strategy established during training. In this study, the Kalman filter decoder was trained using closed-loop decoder adaptation, making decoder stability without recalibration a particularly stringent benchmark. We fixed the decoder after initial training, so that later performance reflected electrode stability rather than ongoing algorithmic adaptation. Using a light-weight, train-once decoder also better matches practical wearable use, where computational complexity adds constraints on processing and power. The ability of the CNT electrodes to preserve decoder performance over 14 days without retraining therefore demonstrates a level of long-term stability beyond conventional signal-quality metrics or discrete classification accuracy.

We extended the myoelectric interface study to a two-week wear period and evaluated higher-dimensional user-involved performance metrics, including time to target, lateral deviation from the optimal path, and cursor trajectories (Fig. 7). Across this extended period, CNT electrodes showed minimal degradation in task-level performance. We also analyzed direction-dependent EMG patterns from each electrode pair and found that CNT electrodes maintained stable, high-fidelity activation patterns over extended wear, whereas wet electrodes failed to preserve comparable EMG patterns over 48 h.

We have discussed potential failure mechanisms in the revised manuscript. Based on our observations, the CNT film itself is not the primary failure point: instead, possible long-term failure modes are more likely to arise from device-level integration, including the transition between stretchable, flexible, and rigid components, the electrical interconnects, and degradation of the temporary substrate/adhesive used for skin attachment. In addition, because the epidermis continuously renews every 14 to 28 days, periodic electrode replacement or reapplication may be necessary to maintain consistent skin contact and allow skin turnover. Based on our extended-wear results, we recommend replacing the electrodes every two weeks.

2.The authors should provide a more nuanced discussion in the Introduction and/or Discussion that directly compares their work with the state-of-the-art in "stable" dry electrodes. For instance, while PEDOT and other nanostructured dry electrodes are mentioned, what is the current best record for stable, recalibration-free operation in similar myoelectric or other electrophysiology tasks? Although table S2 and S3 provide a comparison, it is not clear enough. A dedicated table or expanded narrative comparing key metrics (duration, performance decay, required retraining frequency) with prior art would solidify the manuscript's contribution, especially the current best record in this field. Without this, the work risks being perceived as an incremental improvement in material performance rather than a paradigm shift in interface stability.

Author Response: We have revised the Introduction and included multi-dimensional comparison across several classes of flexible and dry electrodes in the Supplementary Information (Supplementary Table S2-4). The compared metrics are form factor, contact impedance, signal quality, mechanical stability, motion noise, breathability, wear duration and ML-related performance or stability when available. The comparisons are divided into three categories: nanomaterial-based dry electrodes (Supplementary Table S2), organic and metal thin-film electrodes (Supplementary Table S3), and other emerging electrode technologies

(Supplementary Table S4) such as conductive hydrogels, liquid metals, and micromachined electrodes. We have also revised the introduction to summarize the key findings from these expanded comparisons.

To the best of our knowledge, existing dry-electrode benchmarks do not combine continuous uninterrupted wear, without doffing and re-donning, with multi-day tracking of continuous ML decoder performance, to demonstrate electrode stability. The closest reported ML benchmarks in wearable EMG studies focus on gesture classification, rather than continuous regression-based decoding (Supplementary Table S2-S4), and their wear time is often limited to one session. We did not identify prior electrode studies demonstrating multi-day stability of a continuous myoelectric decoder without retraining under continuous wear conditions and could not report the proposed metrics (duration, performance decay, required retraining frequency).

For epidermal electrodes, continuous uninterrupted wear is ultimately constrained by biology, specifically skin turnover time (14-28 days). Therefore, achieving month-long operation very likely will require more invasive methods such as microneedle electrodes, if the device must remain continuously attached. For longer-term use, a more practical strategy is to use algorithmic compensation (such as adaptive models[5]) or zero-calibration generic models (such as the Reality Labs wristband[6]), so that the user may take the device off to shower or sleep. These algorithmic compensation techniques are beyond the scope of this work, as they can always be implemented on an already-stable electrode interface.

3.The manuscript reports stable electrical performance under 30% strain. Is 30% the maximum tensile strain that the CNT film can sustain? During the stretching process, the relative resistance change appears to decrease. Could this behavior arise from CNT fracture followed by re-alignment or densification of the network, which may create additional conductive pathways? If nanotube fracture does occur, it raises a potential concern regarding the release of nanoscale fragments. In the absence of a protective encapsulation layer, could residual CNT fragments remain on the skin surface after repeated deformation? If so, this may have implications for the long-term biocompatibility and safety of the electrode.

Author Response: The reviewer raised a great observation on decrease in resistance during the stretching process. We have indeed previously observed a training-like behavior in CNT films after repeated mechanical deformation, which we attribute to CNT network realignment, junction sliding, and local buckling.

30% strain was not the maximum tensile strain of the CNT film. This strain level was selected as a physiologically relevant testing condition for epidermal electrodes under skin deformation. There is a valid concern that these CNTs will break during strain, as is common with thin films of mismatched mechanical properties to the substrate. However, there does not appear to be any clear fracture planes which span the film, as shown in the SEM analysis of strained CNT films. Instead, the deformation resulted in localized buckling delamination, which is commonly reported in such percolative nanomaterial-based films. CNT networks are composed of a significant number of CNT-CNT van der Waals junctions, which are significantly weaker than the CNTs themselves. While this demonstration was with single-layer film, we consider the delamination to follow a similar trend with multilayer films. There are a significant number of CNT-CNT junctions that may slide or separate when redistributing the stresses in the network, as well as some separation from the substrate.

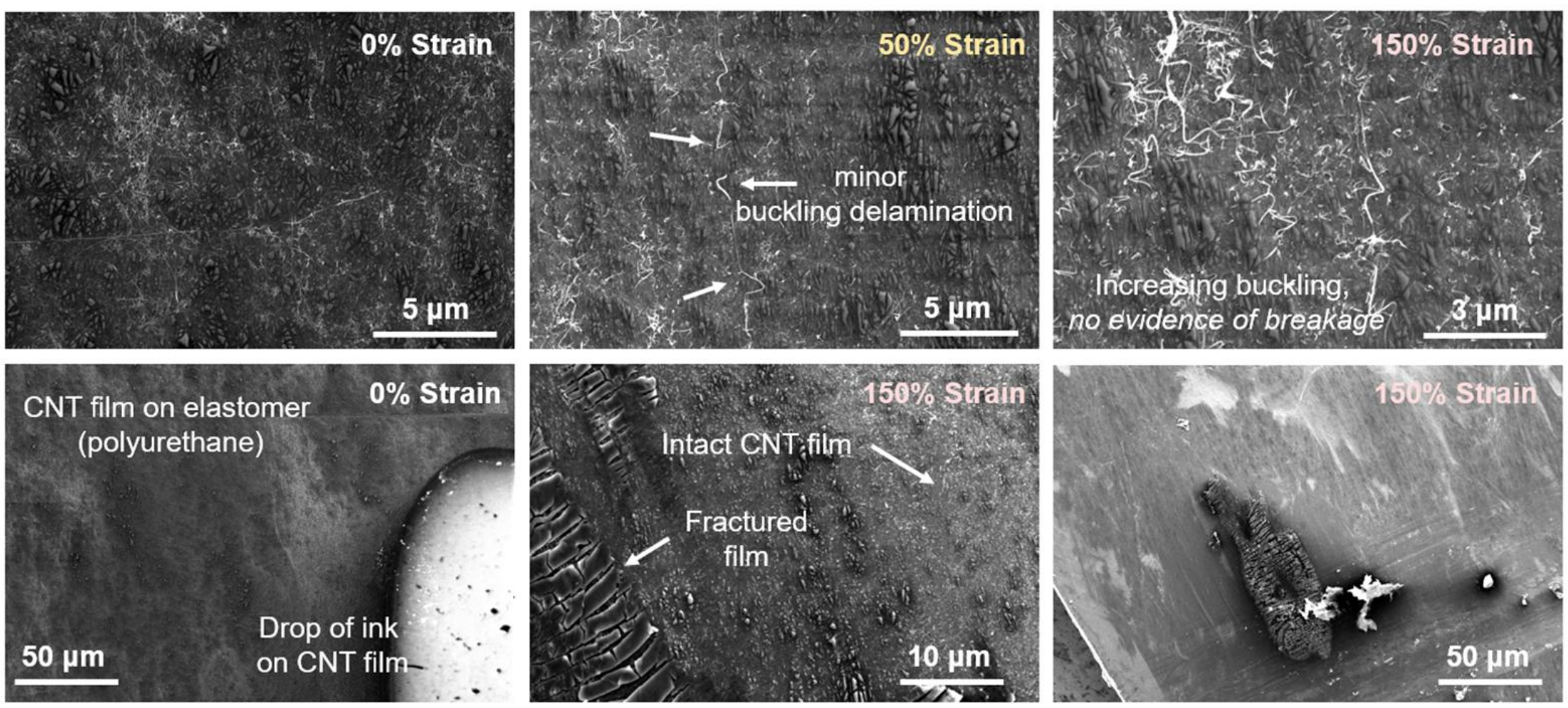

Supplementary Figure S8 | SEM images of CNT films on polyurethane elastomer under various tensile strains.

Possible CNT fracture or fragment release is a valid safety consideration, since the CNT films make direct contact with the skin. To address this point, we added structural-stability analysis after cyclic mechanical strain. Specifically, we performed tacky-transfer tests on 3-layer and 5-layer CNT films after 1000 stretch–release cycles at 35% strain, where the 5-layer film corresponds to the optimized thickness used in the human electrophysiology studies. SEM images of the transfer surface showed no observable CNT fragments or CNT residues after

mechanical cycling, supporting that the interconnected CNT network maintains structural integrity and does not readily shed detectable nanotube debris under the tested conditions.

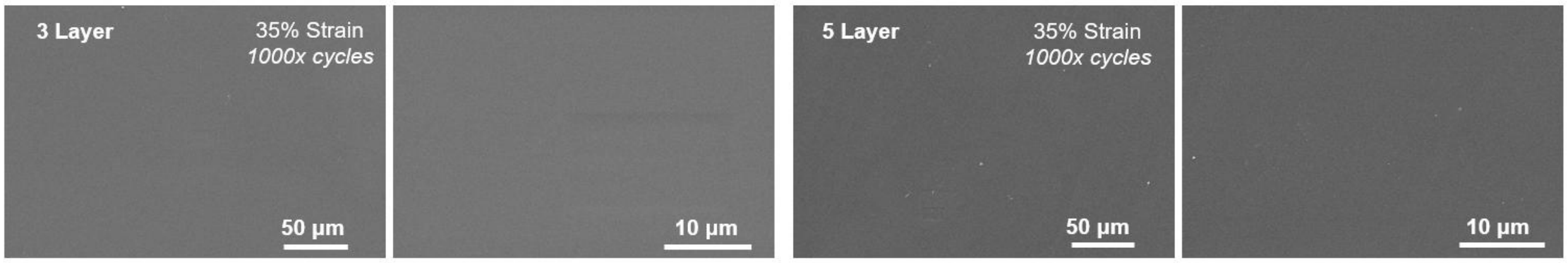

4.The manuscript reports mechanical stability under 30% strain, but it is unclear which film thickness (or optical transmittance) corresponds to this performance. Could the authors specify the transmittance (or thickness) of the CNT film that achieves this level of stretchability? Furthermore, when the film thickness is increased to reduce contact resistance, the mechanical properties (e.g., flexibility, stretchability, and conformability) may change accordingly. Have the authors systematically evaluated how film thickness affects these mechanical properties? Such variations may influence the electrode's ability to maintain conformal contact with the skin and may ultimately affect its long-term stability during practical use.

Author Response: We have clarified in the revised manuscript that the mechanical stability data reported were obtained using CNT films with 90% optical transmittance.

Among the mechanical properties noted by the reviewer (flexibility, stretchability, and conformability), we consider conformability to be the dominant factor affecting electrode performance in this study. The CNT films examined here are in the ultrathin regime, with optical transmittance ranging from approximately 30% to 90%. Within this thickness range, both thin and thick CNT films remain electrically stable under cyclic deformation. Below is a comparison of resistance variation for 90%T and 30%T CNT films after 1000 deformation cycles. Both films showed stable electrical performance. The 30%T film showed smaller resistance variation than the 90%T film, likely because the denser CNT network has lower initial sheet resistance and a larger number of conductive junctions, providing greater pathway redundancy and reducing the effect of local strain on individual junctions.

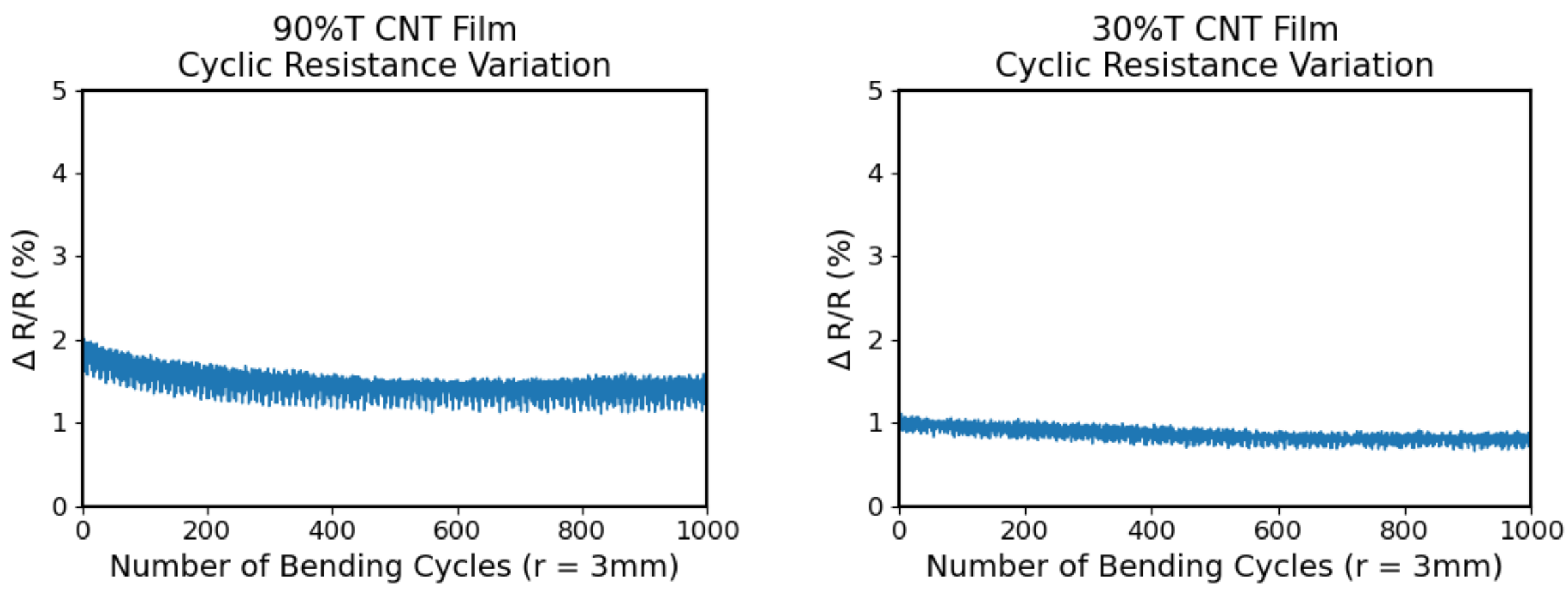

Supplementary Figure S7 | Cyclic resistance response for CNT films of different optical transmittances.

As of conformability, increasing film thickness can influence the electrode's ability to maintain conformal contact with the skin. In the electrode–skin impedance optimization study (Fig. 3c), CNT films showed different degrees of conformity to the skin-replica topography. SEM images showed that thinner films conformed more closely to microscale ridges and valleys, whereas thicker films showed reduced ability to follow the surface topography. This thickness-dependent conformability explains the non-monotonic impedance behavior observed in the manuscript: increasing CNT thickness initially improves electrode–skin impedance due to lower sheet resistance, but beyond the optimized thickness, reduced conformability limits further improvement and can increase the effective contact impedance.

5.Human skin is generally considered a low surface-energy interface, which may make the direct and damage-free transfer of ultrathin CNT films challenging. Could the authors clarify the specific method used to transfer the CNT electrodes onto the skin? If a transfer medium such as temporary tattoo paper is used, it would be helpful to understand whether this intermediate layer affects key electrode properties, such as breathability, mechanical compliance, or electrical contact with the skin.

Author Response: We agree that the transfer process is an important practical point. Although human skin is often described as a low-surface-energy interface, we found the free-standing CNT films readily conform and adhere to most surfaces through van der Waals interactions.

The surface energy of human skin has been reported to be approximately 21–28 mN/m, largely due to the lipid- and sebum-rich outer surface[7]. This range is comparable to that of PDMS[8], which

is also a hydrophobic, low-surface-energy material with reported surface energies of approximately 19–21 mN/m. Prior work[9] has shown that SWCNT thin films can be readily dry-transferred onto PDMS under ambient conditions and the surface adhesion force density is 97.7 nN/Å^2. This is consistent with our own observation that CNT films transfer within seconds without observable breakage to the skin. In practice, we also found that the interconnected nature of the CNT aerogel films also provide better mechanical properties compared to CNT aerosol films and is another contributing factor to easy manipulation and transfer.

The transfer medium was therefore not used to overcome poor CNT–skin adhesion, but rather to enable scalable handling, patterning, alignment, and assembly of the ultrathin CNT electrodes. Free-standing CNT films are difficult to manipulate reproducibly at device scale because they are lightweight, like to stick to most surfaces, and can easily collapse into CNT fibres. The transfer medium provides mechanical support during CNT patterning, conductive-interconnect printing, and device assembly. Bare CNT films are also sensitive to abrasion, so another role of the transfer medium is to protect the CNT film during extended wear.

We have tested several transfer media in prior device iterations, including PU, PDMS, and waterproof adhesive films. In this study, temporary tattoo paper was selected because it is thin, breathable, and mechanically compliant, and shows reliable transfer of the CNT film onto skin. We found that when the adhesive release layer is gently dissolved from the substrate liner during the water-assisted transfer process, it helps the electrodes conform closely to the skin surface. The contact impedance, ExG recording, and cursor-control results reported in the manuscript were all measured using this final electrode configuration and therefore already account for the effect of the transfer medium on breathability, mechanical compliance, and electrical contact.

6.As for the depth of multi-day demonstration,the performance decay of the wet electrodes is attributed to gel dehydration/signal drift. Could the authors provide direct, simultaneous measurements of the impedance or a simple signal quality metric (e.g., SNR, baseline noise) for both CNT and wet electrodes over the same long-term period, such as several weeks? This would directly correlate the observed performance stability/degradation with the hypothesized interfacial stability/drift. Figure 3h shows impedance variation under disturbance, but not long-term drift.

Author Response: We added longitudinal plots of skin–electrode impedance (Fig. 5L) and showed EMG modulation variation (Fig. S20) and signal drift (Fig. S21) for CNT and wet electrodes. The CNT electrodes maintained relatively stable impedance and SNR over the

extended wear period. The wet electrodes could not be evaluated for longer than 48 h duration because prolonged wear posed a significant risk of skin irritation to the participant, that we have observed in the past (Fig. 4). For the 48 h wet-electrode comparison, the wet electrodes were covered with a waterproof layer to preserve the hydrogel from swelling and losing adhesion during showering. As a result of this occlusive condition, we observed a decrease in wet-electrode impedance over time, likely due to increased hydration of the skin. However, such reduction in impedance did not translate into stable myoelectric decoding performance because of the large channel specific impedance variation over time. The wet-electrode experiments showed substantial instability within 48 h, including increased failure rate in completing the task and degraded performance metrics. This result highlights that impedance alone is not sufficient to predict ML-relevant interface stability. Stable myoelectric decoding requires preservation of the multichannel EMG feature distribution, including relative channel amplitudes, timing, noise level, and electrode-specific activation patterns. These results are consistent with a recent HDEMG study[1] showing that modest average impedance changes and stable SNR can coincide with statistically significant electrode-specific impedance variability, which significantly decreased the accuracy of gesture recognition for classifiers that rely on consistent patterns of sensed myoelectric activity over time.

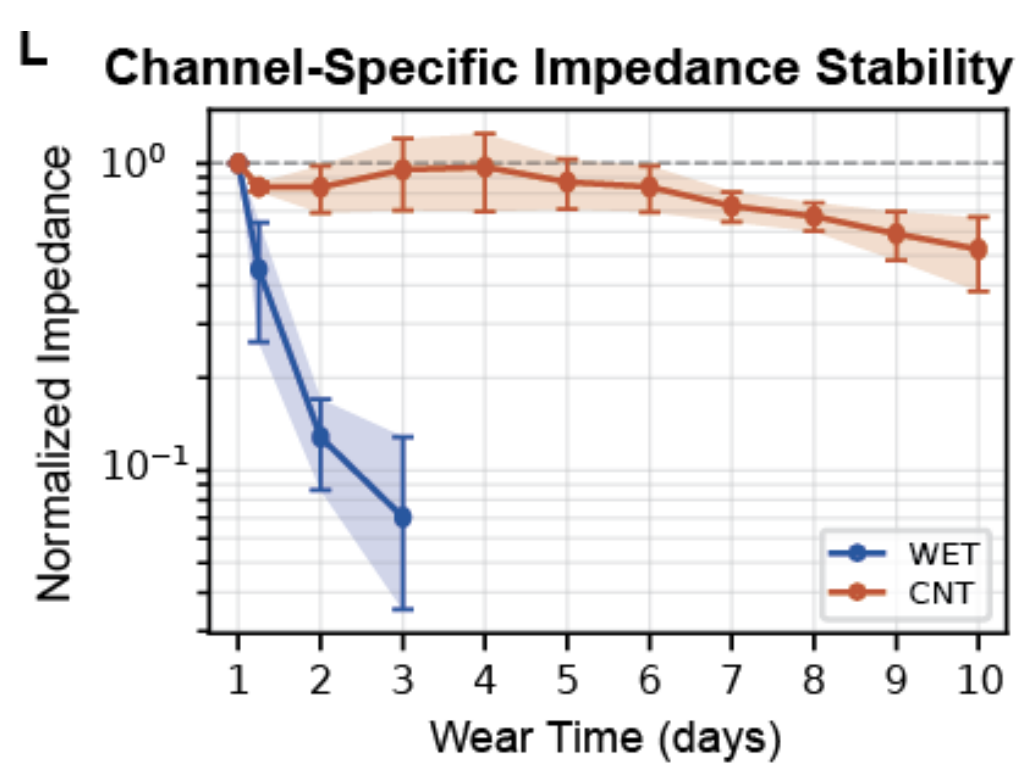

Figure 5L | Normalized channel-specific impedance variation over 10-day wear for wet (n = 8) and CNT electrodes (n = 10).

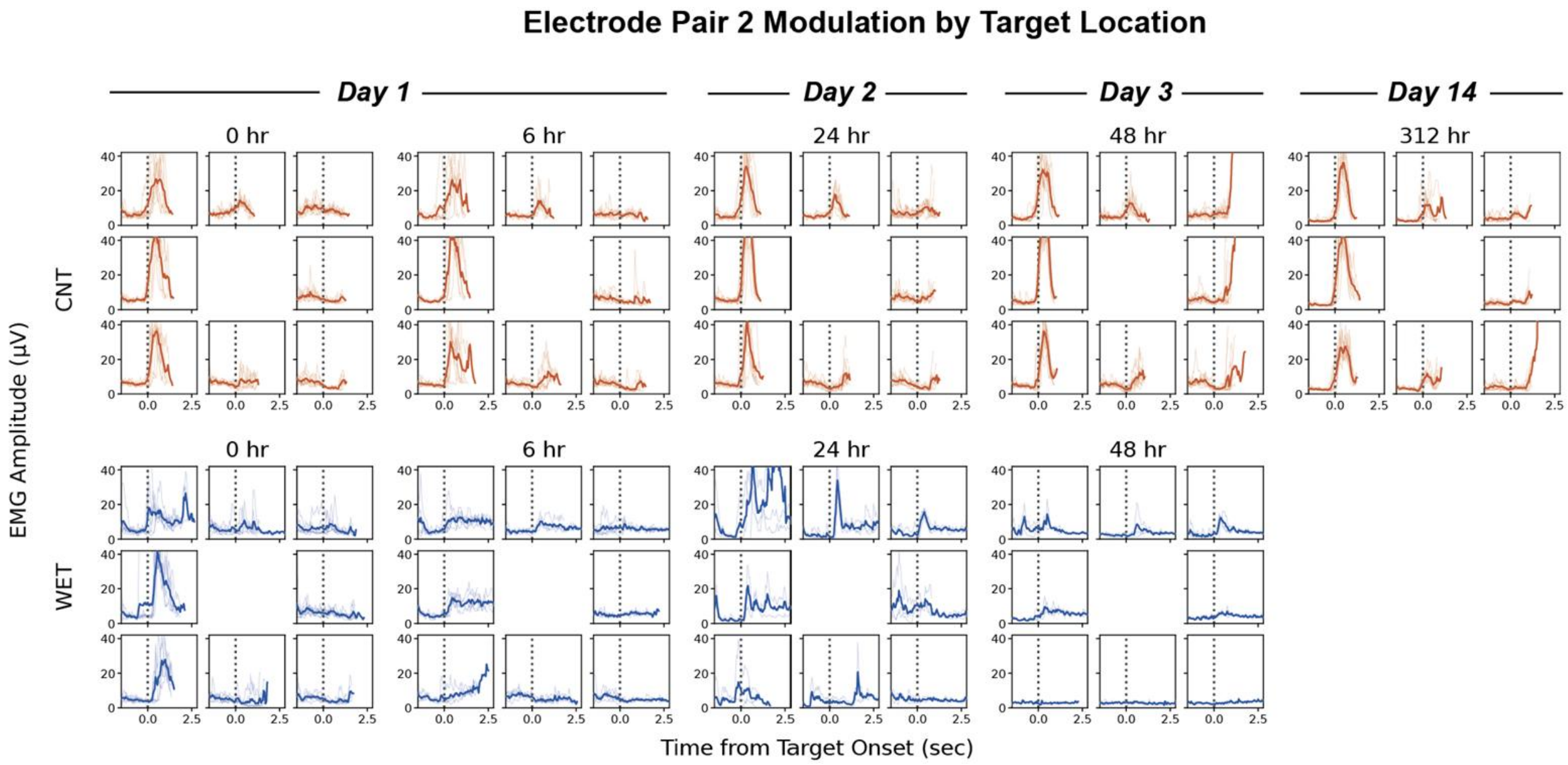

Supplementary Figure S19 | EMG modulation from electrode pair 2 over extended wear, grouped by target location. EMG RMS traces from electrode pair 2 are shown for CNT dry electrodes and wet gel electrodes across cursor control sessions at 0, 6, 24, 48, and 312 h after initial electrode placement. Wet electrodes failed after 48 hours. Each small panel corresponds to one target location, arranged according to the center-out target layout. CNT electrodes preserved clear target-dependent modulation from Day 1 to Day 14, whereas wet electrodes showed significant variation in modulation patterns from Day 1 to Day 3. Dark traces represent the average values, and lighter traces represent individual trials. The vertical dashed line marks target onset.

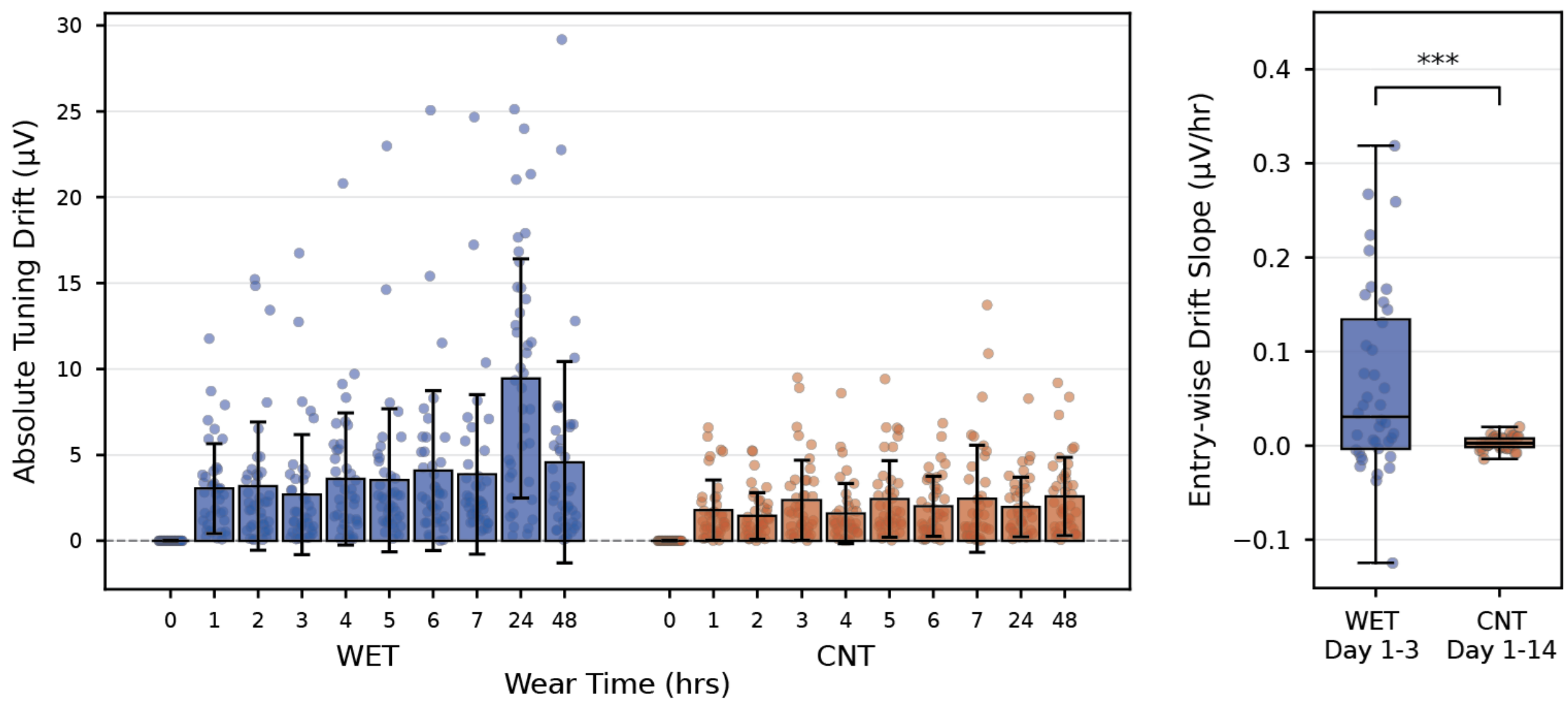

Supplementary Figure S20 | EMG tuning drift during extended wear. Left, absolute EMG tuning drift was quantified over time for wet gel electrodes and CNT dry electrodes. Points represent individual measurements. Wet electrodes showed larger and more variable tuning drift over the first 48 h, whereas CNT electrodes maintained lower drift. Right, tuning drift slopes were compared for wet gel electrodes and CNT dry electrodes. CNT electrodes were calculated across 2 weeks of wear whereas the wet electrodes were measured across 2 days and failed afterwards. CNT electrodes exhibited significantly smaller drift slopes over Day 1–14 than wet electrodes over Day 1–3, indicating improved stability of target-dependent EMG tuning during prolonged wear.

7.The manuscript attributes stability to the material properties (porosity and Van der Waals adhesion). While plausible, the analysis is somewhat phenomenological.A more mechanistic investigation is needed. For example: The role of Van der Waals adhesion in maintaining conformal contact is emphasized. Are there quantitative data on adhesion energy or measurements of contact conformity (e.g., via optical coherence tomography) after several days of wearing, including during/after activities like hand washing or sweating?

Author Response: We agree that quantitative adhesion-energy measurements or OCT-based contact mapping would provide a more complete mechanistic description of the CNT–skin interface. In this study, we did not perform adhesion energy measurements or OCT-based contact mapping. Prior studies have quantitatively investigated the adhesion and interfacial strength of CNT thin films and arrays on various substrates[9–11], showing that CNT films can adhere to diverse

substrates, including a-Si:H, Si, SiO2, PDMS, and glass through Van der Waals force. We have cited relevant literature in the revised manuscript.

Within the present study, we provide two types of evidence supporting maintained electrode–skin contact after prolonged wear. First, longitudinal skin–electrode impedance measurements showed that CNT electrodes maintained stable contact impedance over time, providing electrical evidence that the electrode–skin interface remained stable. Second, we included photographs of CNT films on skin after three days of continuous wear as visual verification. The close-up post-wear image shows several CNT electrodes viewed from the side and from the top. The side view shows no visible edge lifting, peeling, or delamination. The front view shows that microscopic skin texture remains visible through the CNT film, consistent with close contact of the ultrathin film with the skin surface.

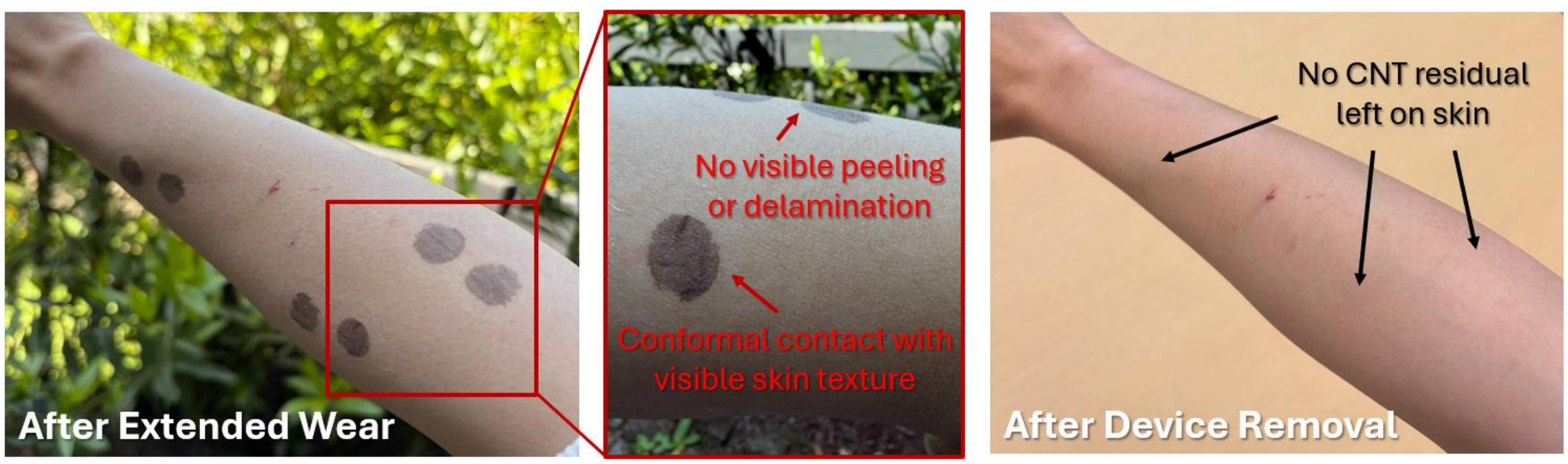

8.The claim that CNTs are electrochemically stable in sweat/body heat is not supported by new data. Please provide potentiostatic electrochemical impedance spectroscopy (EIS) data for the CNT film in an artificial sweat solution at 37°C over several days, comparing it to a benchmark material like PEDOT:PSS, and some other commonly used nanomaterials.

Author Response: We added potentiostatic electrochemical impedance spectroscopy (EIS) measurements of CNT films in artificial sweat (PBS) at 37 °C and included PEDOT as a benchmark electrode material for comparison. The CNT films remained stable for up to 4 weeks, supporting that the CNT electrode material itself does not undergo substantial electrochemical degradation in artificial sweat at body-relevant temperature. PEDOT films showed substantial changes after 2 days under the same conditions, consistent with electrochemical and hydration-induced changes in the conductive polymer film.

We have added these new EIS data to the Supplementary Information to support the statement that CNT films are electrochemically stable under sweat- and body-heat-relevant conditions.

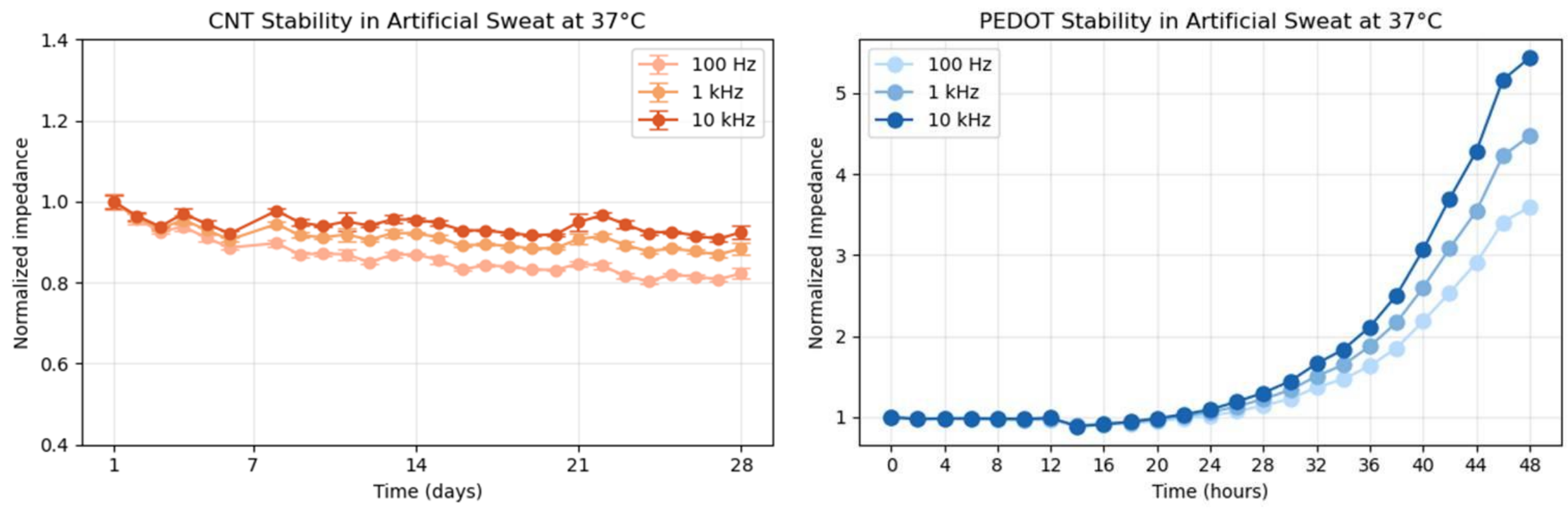

Supplementary Figure S9 | Electrochemical stability of CNT electrodes during continuous immersion in artificial sweat at 37°C compared with PEDOT electrodes. CNT electrodes maintained stable impedance over 28 days. PEDOT electrodes showed a rapid increase in normalized impedance within 48 h, indicating degradation of electrochemical stability in artificial sweat at elevated temperature.

9.The description of decoders D1-D3 is confusing. It states they were trained "for comparison" (Line 347). The conclusion that retraining yields no improvement for CNT electrodes is vital. This should be shown directly in the main figure, perhaps by adding a panel comparing the performance of D0 vs. D1/D2/D3 at the 6h, 24h, 48h time points for CNT and wet electrodes side-by-side.

Author Response: We have revised the manuscript to clarify the decoder definitions. Decoder D0 refers to the initial decoder trained at the beginning of the experiment. Decoders D1–D3 refer to decoders retrained at later time points, corresponding to the 6 h, 24 h, and 48 h sessions, respectively. Fig. 6f shows the direct comparison between D0 and the retrained decoders D1–D3 at the corresponding 6 h, 24 h, and 48 h time points for both CNT and wet electrodes.

10.As for the task complexity,the center-out task is standard. To better showcase the stability's importance, could the authors include a more dexterous task (e.g, a virtual keyboard typing task requiring discrete selections) at the beginning and end of the 48-hour period using the sameinitial decoder (D0)? This would powerfully demonstrate the real-world utility of a "set-and-forget" interface.

Author Response: We added an obstacle-avoidance cursor-control task in the revised manuscript (Fig. 7). In this task, an obstacle was placed between the home position and the target, requiring the user to guide the cursor around the obstacle rather than moving directly along the shortest path. This task preserves the continuous-control nature of the original center-out task while adding greater demands on trajectory planning and fine control of cursor direction and velocity.

In addition, the obstacle-avoidance task was performed using the same initially trained decoder, without additional retraining or recalibration. Therefore, the task directly tests whether the original decoder remains usable for a more demanding control after prolonged electrode wear. As shown in the revised manuscript, the user slowed down and navigated the cursor more deliberately during the obstacle-avoidance task, as reflected by increased time to target and altered cursor trajectories compared with the standard center-out task. Despite this increased task difficulty, CNT electrodes maintained stable task performance over two weeks, supporting the potential utility of the interface for real-world applications that require sustained, fine closed-loop control, such as neurorehabilitation and assistive interfaces.

Minor Comments:

1.Abstract/Title: The title and abstract emphasize "Multi-day Recalibration-free." The data only supports 48 hours (2 days). "The authors should be precise in the text (e.g., "two days").

Author Response: We have extended the myoelectric-interface study to 14 days, as suggested by previous comments.

2.Fig.2k:The radar plot is useful. Please ensure the axes are clearly defined in the caption or figure. What is the source of the data for PEDOT and metal nanowire benchmarks? Cite appropriately.

Author Response: We have revised Fig. 2k, its caption, and the main text to define the axes and included sources of the benchmarks.

References:

1. Yang, J., Shibata, K., Weber, D. & Erickson, Z. High-density electromyography for effective gesture-based control of physically assistive mobile manipulators. *Npj Robot.* **3**, 2 (2025).
2. Chetyrkina, M. R., Fedorov, F. S. & Nasibulin, A. G. In vitro toxicity of carbon nanotubes: a systematic review. *RSC Adv.* **12**, 16235–16256.
3. Dewald, H. A. *et al.* Stable, three degree-of-freedom myoelectric prosthetic control via chronic bipolar intramuscular electrodes: a case study. *J. NeuroEngineering Rehabil.* **16**, 147 (2019).
4. Kim, J. *et al.* Ultrathin Soft Wearable Sensor Materials and Structures: A Review of Current Trends and Prospectives. *ACS Appl. Mater. Interfaces* **17**, 59994–60027 (2025).
5. Abdallah, I. B., Bouteraa, Y. & Alotaibi, A. A hybrid EMG–EEG interface for robust intention detection and fatigue-adaptive control of an elbow rehabilitation robot. *Sci. Rep.* **15**, 40895 (2025).
6. Kaifosh, P. & Reardon, T. R. A generic non-invasive neuromotor interface for human-computer interaction. *Nature* **645**, 702–711 (2025).
7. Khyat, A. E., Mavon, A., Leduc, M., Agache, P. & Humbert, P. Skin critical surface tension: A way to assess the skin wettability quantitatively. *Skin Res. Technol. Off. J. Int. Soc. Bioeng. Skin ISBS Int. Soc. Digit. Imaging Skin ISDIS Int. Soc. Skin Imaging* **2**, 91–96 (1996).
8. Vudayagiri, S., Junker, M. D. & Skov, A. L. Factors affecting the surface and release properties of thin polydimethylsiloxane films. *Polym. J.* **45**, 871–878 (2013).
9. M Rajanna, P. *et al.* Adhesion of Single-Walled Carbon Nanotube Thin Films with Different Materials. *J. Phys. Chem. Lett.* **11**, 504–509 (2020).
10. Xu, M., Du, F., Ganguli, S., Roy, A. & Dai, L. Carbon nanotube dry adhesives with temperature-enhanced adhesion over a large temperature range. *Nat. Commun.* **7**, 13450 (2016).
11. Zhao, Y. *et al.* Interfacial energy and strength of multiwalled-carbon-nanotube-based dry adhesive. *J. Vac. Sci. Technol. B Microelectron. Nanometer Struct. Process. Meas. Phenom.* **24**, 331–335 (2006).
